# Hybrid approach to structure modeling of the histamine H3 receptor: Multi-level assessment as a tool for model verification

**DOI:** 10.1371/journal.pone.0186108

**Published:** 2017-10-05

**Authors:** Jakub Jończyk, Barbara Malawska, Marek Bajda

**Affiliations:** Department of Physicochemical Drug Analysis, Faculty of Pharmacy, Jagiellonian University Medical College, Krakow, Poland; University of Parma, ITALY

## Abstract

The crucial role of G-protein coupled receptors and the significant achievements associated with a better understanding of the spatial structure of known receptors in this family encouraged us to undertake a study on the histamine H3 receptor, whose crystal structure is still unresolved. The latest literature data and availability of different software enabled us to build homology models of higher accuracy than previously published ones. The new models are expected to be closer to crystal structures; and therefore, they are much more helpful in the design of potential ligands. In this article, we describe the generation of homology models with the use of diverse tools and a hybrid assessment. Our study incorporates a hybrid assessment connecting knowledge-based scoring algorithms with a two-step ligand-based docking procedure. Knowledge-based scoring employs probability theory for global energy minimum determination based on information about native amino acid conformation from a dataset of experimentally determined protein structures. For a two-step docking procedure two programs were applied: GOLD was used in the first step and Glide in the second. Hybrid approaches offer advantages by combining various theoretical methods in one modeling algorithm. The biggest advantage of hybrid methods is their intrinsic ability to self-update and self-refine when additional structural data are acquired. Moreover, the diversity of computational methods and structural data used in hybrid approaches for structure prediction limit inaccuracies resulting from theoretical approximations or fuzziness of experimental data. The results of docking to the new H3 receptor model allowed us to analyze ligand—receptor interactions for reference compounds.

## Introduction

G-protein coupled receptors (GPCRs) constitute one of the largest and most important groups of human receptor superfamilies[1]. They represent a very important focus for studies on bioactive substances and the search for new drugs. It is estimated that more than 50% of all discovered drugs interact with the GPCR receptors[2]. The Nobel Prize in Chemistry awarded in 2012 to Robert J. Lefkowitz and Brian K. Kobilka "for the study of G-protein coupled receptors" highlights the importance of research which leads to understanding the mechanisms of action of active substances toward these receptors. The histamine H3 receptor (H3R) belongs to the family of receptors coupled to G-proteins. It occurs widely in the central nervous system (CNS)[1], but recent studies have also reported its presence in peripheral tissues[3]. H3R is linked to G_α_ subunit type G_i_/G_0_ which, after receptor activation, inhibits adenylyl cyclases and Na^+^/H^+^ exchangers[4]. However, the greatest impact on signaling pathways has released G_βγ_ subunit complex which inter alia activates phospholipases C and A_2_, and kinases PI3 and MAP and inhibits N and P/Q type voltage gated Ca^2+^ channels[4–7]. Blockage of that last signaling pathway is associated with the inhibition of neurotransmitter release upon activation of the histamine H3 receptor[8]. As an autoreceptor, it inhibits the release of histamine from histaminergic nerve terminals[9]. As a heteroreceptor, the histamine H3 receptor modulates the release of other neurotransmitters, including acetylcholine, serotonin, noradrenalin, dopamine, glutamate and GABA[10,11]. The histamine H3 receptor is characterized by a high constitutive activity[12]. Due to the wide range of functions of the H3 receptor, its deployment and the positive results of pharmacological studies on animals, many academic research groups and leading pharmaceutical companies have chosen agonists and antagonists of H3R as their targets in the search for new effective agents in multiple diseases connected with neurotransmission dysfunctions[13–16]. The ligands of H3R belong to different chemical classes of compounds. Studies on antagonists and inverse agonists of the H3 receptor have been developed most effectively. Thioperamide, the first selective agent for H3R, and clobenpropit, a commonly used reference ligand in research on the H3 receptor, are representatives of a large group of imidazole containing compounds[17,18]. The second group of ligands, which contains the non-imidazole derivatives, has been developed because of the sensitivity of the imidazole compounds to cytochrome P450[19]. To date, the use of H3R antagonists and inverse agonists has been proposed in the treatment of obesity, narcolepsy, epilepsy, ADHD or Alzheimer's disease[20–23]. Three non-imidazole compounds with inverse agonist / antagonist activity are in an advanced stage of human testing. Clinical trials of GSK239512 have demonstrated its efficiency and safety among patients with mild to moderate forms of Alzheimer's disease[24]. MK-7288 has demonstrated efficiency among patients with excessive daytime sleepiness[25]. In 2016 pitolisant, marketed under the trade name Wakix, has obtained authorisation throughout the European Union (EU) for the treatment of narcolepsy with or without cataplexy in adult patients. The main evidence of efficacy of pitolisant was based on two, successfully, Phase III clinical trials[26]. Despite the passage of more than 30 years since the discovery of the histamine H3 receptor and the 15 years since its sequencing, it has not been crystalized[27,28]. The three-dimensional structures of the protein—ligand complexes provide very important information about the ligand interactions with the biological target. This knowledge is essential in the design of new bioactive substances. Significant development of genomic sequence analysis has led to the discovery of hundreds of thousands of proteins and it has been assumed that at least a thousand of them are GPCRs[29]. Unfortunately, due to the limitations of the experimental methods such as X-ray crystallography or NMR spectroscopy, only a small part of the known GPCRs have so far been studied using these methods. Homology modeling is an opportunity to achieve faster insights into the structure of proteins. Recent studies have shown that approximately 33% of all known proteins present sequence similarity to proteins with known structures[30]. This type of sequence similarity is directly related to the structural similarity of the proteins and forms the basis for homology modeling. Homology modeling enables the creation of a model of the test protein based on the evolutionary similarity to the structure of the protein-template employed. This technique has already been applied for the creation of the variety of protein models, among others in Critical Assessment of protein Structure Prediction (CASP) experiments[31,32,33]. In the case of the histamine H3 receptor, older models are mostly created from the template of the crystal structure of bovine rhodopsin[34]. Recently generated models have mainly been produced using the template of the histamine H1 receptor[35]. Unfortunately, there are no crystallographic data that would allow for verification of these models. However, recently published data and the improvement of available tools for modeling allow us to propose new, more accurate models of H3R as well as a novel approach for the verification of models already created. We used different homology modeling tools to generate a diversified model library. The prepared library was assessed using the hybrid evaluation approach involving both knowledge-based and ligand-based methods. This allowed us to combine the benefits of using each method: improvement of the overall fold of a protein with selection of models based on specific knowledge-based potentials; and a detailed analysis of the local arrangement of amino acids inside the binding site by ligand-based selection. Arrangement of amino acids within the active site was particularly important for us because of the further use of models in the study of ligand—receptor interactions and design of novel ligands.

## Results and discussion

### 1. Sequence alignment analysis

The sequence alignment of the human histamine H3 receptor (hH3R) and templates was carried out based on two algorithms. Clustal Omega is a multiple sequence alignment program that uses seeded guide trees and HMM profile-profile techniques to generate alignments[36]. MSAProbs is based on a combination of pair hidden Markov models, partition functions calculating posterior probabilities, weighted probabilistic consistency transformations and weighted profile-profile alignments[37]. Twelve alignments were generated: *i*.*e*. alignments for each template (hH1R and rM3R) and alignments by each program based on the two matrices (S1 Table). Comparison of received alignments revealed a very high similarity between the results from both programs. The transmembrane helices are characterized by the highest conservation degree in the GPCR family; however, the analysis allowed us to show some differences in similarity between the matrices we used. Transmembrane helices (TMH) 1–4 and 7 of hH3R are closer sequentially to rM3R. On the other hand, helix 5, which is most diversified within aminergic GPCRs[38], and helix 6 show higher similarity to hH1R. This is reflected in the participation of these fragments in the binding of ligands specific for each receptor. The greatest variations between alignments were observed within loops. Alignments built on the histamine H1 receptor template had a 6–7 amino acid gap within ECL2, alignments built on the muscarinic M3 receptor had single amino acid gap in ECL3. Mixed template alignments had 2 or 3 single amino acid gaps in both ECL2 and ECL3. Observed differences were the results of the different sequence similarities of individual fragments of loops. They had the biggest impact on the shape of ECL2. Although there is a very large diversity in this fragment among all GPCRs, the patterns used do not reproduce this fragment of the receptor in an appropriate way. The loop derived from histamine H1 receptor crystals is broken and cannot provide a good representation for future models. On the other hand, this loop in rM3R from 4U15 is complete, but it is a fragment of the lowest homology to the target protein.

### 2. Energetic and qualitative assessment of models

Assessment of model quality is the critical step in homology modeling. During the verification process, all models were passed through BCL::Score and QMEAN. BCL was developed to predict the global fold of a membrane protein. It is a customized, knowledge-based energy function that provides the most accurate evaluation of the arrangement of secondary structure elements. This enriches native-like topologies in diverse sets of protein models by calculating the overall energy potential as a linearly weighted consensus scoring function[39]. QMEAN, *i*.*e*. Qualitative Model Energy Analysis, is a composite scoring function describing the major geometrical aspects of protein structures[40]. Criteria for initial selection of models were established based on the cutoffs for BCL::Score and QMEAN scores, which were equal to −2200 and 0.475, respectively. Cutoffs were determined based on assessments of templates, where the model of hH1R was scored as −2145.76 and 0.460, and the model of rM3R as −2092.23 and 0.512. This led to the identification, among 1969 models, of the 259 top-scored models with the highest probability to reproduce the spatial structure of the receptor. Clear differences between the models produced on different templates and generated by different programs were observed. For all models, structures generated on a multi-template alignment (automodel/slow refinement) were assessed by BCL::Score with a top value of -2351.97, and a model generated on an rM3R template (MyLoop/ very slow refinement) obtained the highest QMEAN score value of 0.535. A total of 160 of the remaining models were generated on an rM3R template and 91 on a mixed template. Only one model built on a single hH1R template (MyLoop/slow refinement) was included for further studies. This information shows that even a small difference in the quality of a pattern, such as that between hH1R and rM3R, causes a marked difference in the very first stage of model verification. In the case of program differences, none of models obtained using the nest protocol from the Jackal program obtained the appropriate score values. Likewise, most of the models generated by SwissModel and I-TASSER services were eliminated. Of the 259 models, 252 were generated by Modeller. A thorough analysis of models prepared by Modeller showed that the largest group of models which passed a preliminary assessment were generated with the help of automodel or loopmodel protocols. Also, clearly an intermediate degree of refinement was more frequent among the highly rated models. We also found that the best protocols available in the Modeller for structure refinement are slow and fast options, and these were used for more than 90% of models approved for further study. The stereochemical quality of 259 models was evaluated using PSVS suite[41]. PSVS integrates analyzes from several widely-used structure quality evaluation tools, including RPF, PROCHECK, MolProbity, Verify3D and Prosa II. Global quality measures are reported as Z-scores based on calibration with a set of high-resolution X-ray crystal structures. In order to obtain a homology model of high quality as close as possible to the quality of crystal structures, it was decided to compare the PSVS scores of selected models with the scores of templates employing PSVS Z-score values. Built models were characterized by Verify3D and Prosa II, and such scores are very similar to the score values of crystal structures used as the templates. Both scoring algorithms concentrate on an assessment of the correctness of the 3D model structure based on an analysis of the fragments and relationships of the primary structure of proteins to the secondary and tertiary structure (Verify3D) or energy of models (Prosa II), in combination with information from the solved protein structures database. On the other hand, models had low values for MolProbity Clashscore and Procheck Z-scores. MolProbity Clash analysis considers steric overlaps of 0.4Å or greater between non-bonded atoms. The high degree of amino acid packing in homology models is probably related to the optimization processes used in the modeling tools which omit the presence of water and have much less restrictive limits on impact-type van der Waals interactions. Procheck assesses the degree of deviation of geometry of each of the residues in a given model. Information about how common or, alternatively, how unusual the assessed protein geometry is can be determined from a comparison with stereochemical parameters derived from high-resolution structures. Highlighted regions do not only signify wrong fragments, but may be reasonably explained by conformational changes due to ligand-binding processes. This is supported by the highly rated by Prosa II models that were eliminated during the second stage of assessment associated with the docking of ligands due to the closing of the active site by amino acid residues which change their position during ligand binding. All models whose assessment indicated significant divergences with the structure of known proteins were excluded from further study. Visual inspection of models showed that transmembrane helices were very similar across all highly-rated models. There were no significant differences in the lengths of these fragments, whether resulting from the application of different templates or various programs. All models were characterized by kinks in helices at, respectively, amino acids A2.55, L4.55, I4.61, E5.46, C6.47 and S7.46. Significant diversification was observed for loops. Modeled ICLs had mostly helical conformation that varied in both the length and folding degree of the helices. All models top-rated by BCL::Score and QMEAN presented were helically shaped fragments in ICL1 and ICL2. Significant changes applied to ECL2. The ECL2 loop itself is a very conformationally diversified fragment of the receptor with a relatively large freedom. This results in the generation of many different intermediate conformations during the homology modeling process. In the case of the tested receptor, the main variable feature was the degree to which ECL2 adopts the alpha helix conformation. Of all the generated structures, models with a strongly marked helix and all stages leading to the development of a fully disordered structure were present. Surprisingly, based on scoring program rating, only models without a helical motif within ECL2 were qualified to the next stage of research. In the following step of the study, the differences in the positioning of amino acid residues within ECL2 proved to be essential for proper ligand binding. Among the important aspects of the aminergic GPCR extracellular loops are disulfide bridges. The structure of crystallized receptors closely related to the histamine H3 receptor (histamine, muscarinic and adenosine receptors) indicates the presence of at least two disulfide bridges. The first disulfide bridge stabilizes ECL2, combining it with the extracellular part of TMH3. The second disulfide bridge connects the two cysteine residues present in ECL3. During the manual assessment, models which did not have both of the previously described disulfide bridges were discarded. Visual analysis also took into account the state of the modeled receptor and its agreement with GPCR inactive conformation. Preparation of the homology model should be performed with particular attention being paid to those ligands with which the developed model will interact in the subsequent experiment. Models used for investigation of antagonists / inverse agonists should accurately reproduce the inactive state of the receptor preferred by this type of ligand. The literature outlines a number of conserved aliphatic and polar amino acids within the class A of GPCR receptors involved in the stabilization of the receptor in inactive state[42,43,44]. Among the rhodopsin family of G protein coupled receptors, a number of "switches" have been described: groups of amino acids where conformational changes determine the activation of the receptor. These include: Ionic Lock Switch associated with the motife DR(Y/F); 3–7 Lock Switch; Transmission Switch on TMH6 containing the motif CWxP; and Tyr Toggle Switch on TMH7 connected with the NPxxY motif. A research group led by Prof. Filipek examined the GPCR “molecular switches” based on the available crystal structures, indicating the preferred conformation from among these motifs of amino acids in inactive and active receptor states[42]. During visual selection of our top scored models, we were focused on the analysis within these important regions and for further investigation we chose only models with amino acid interaction corresponding with that described in the literature. The first analyzed motif was DRF and the so-called Ionic Lock. In selected models, residues within DRF were well reproduced. The presence of D6.30 instead of E6.30 prevents the development of ionic bonding characteristic for that region. The absence of an ionic lock in the crystal structure was observed in many GPCR receptors, including human β2AR, CXCR4 and human H1R[42]. However, in these cases Ionic Lock residues participate in other polar interactions, such as the hydrogen bond between D3.49 and Y3.60 and interaction between R3.50 and S6.36 (analogous to R3.50 and Q6.36 in hH1R)[45]. A similar situation in our models was observed during evaluation of interactions within a 3–7 lock. For the first time, the 3–7 lock has been described in the rhodopsin receptor as the interaction between E3.28 and K7.43. The same mechanism was also proposed for aminergic receptors where TMH3 and TMH7 interact with conserved residues D3.32 and Y7.43. The literature suggests breaking of those polar bonds as the first step that promotes the activation of the receptor. In the case of our modeled H3 receptor, the 3–7 lock can be considered interaction between D3.32 and W7.43. Among the best models, we identified a specific arrangement of those highly variable residues in which a nitrogen atom in the heterocyclic ring of the tryptophan can form a hydrogen bond with the carboxyl group D3.32. Tryptophan is also an essential amino acid in another switch located within the conserved motif CWxP in TMH6. This switch is called a transmission switch and is involved in the reorganization of 3–5–6 transmembrane helices during activation[46]. Among models selected for the docking stage, we observed two dominant positions of W6.48, parallel and perpendicular with respect to the axis of helices. The first was similar to W6.48 in inactive conformation of human adenosine A_2A_ receptors, the second identical to W6.48 in inactive human muscarinic receptors M1, M2 and rat M3 when used as a template. Comparing crystal structures, we observed that inactive conformation of tryptophan in CWxP motif in the analyzed receptors is associated with the type of amino acid at position 7.42. In β2 adrenergic receptors and histamine H1, there is a G7.42 which allows W6.48 to reach full parallel position. A_2A_ receptors with A7.42 prevent such conformational freedom and allow only “almost” parallel arrangement. In the case of S7.42 from muscarinic receptors, longer sidechains cause large steric hindrance, and most arrangements in crystal structures are perpendicular. The modeled H3 receptor has leucine in position 7.42 which, similar to serine, forces a perpendicular arrangement of the W6.48 rings. Results of further studies described in a subsequent part of this manuscript indicate that this is the position favored by H3 ligands in docking studies. This may be significant for differences in the recognition of specific ligands. The last of the described switches is associated with the existence of a hydrophobic barrier in A class GPCRs[47]. In hH3R this is made up by five residues: L2.43, L2.46, I3.43, I3.46, and I6.40. In the inactive state, these residues move towards to the center of the receptor and this creates a hydrophobic separator between the existing network of hydrogen bonds and the DRF motif. During receptor activation, progressive helix reorganization causes dispersal of the residues forming the barrier. Further changes in TMH5 position allow progression of the hydrogen bond network involving DRF through Y7.53 from conserved motif NPxxY, which in active state is directed to the inside of the receptor. In the generated models, we observed a situation identical to that in the other crystals of aminergic GPCR in non-active conformation. The hydrophobic barrier is maintained and the Y7.53 is isolated from the hydrogen bond network. Conformation of Y7.53 is the same as in the crystal structure of histamine H1, muscarinic M1–M4 or adenosine A2A in inactive state. Differences in the recurring scheme of hydrogen bonds between the active and inactive states of receptors were another element on which we focused our attention during the visual assessment of models. Ron O. Dror and Brian Kobilka highlighted polar interactions between 1.50, 2.50, 3.32, 3.39, 5.46, 6.40, 6.44, 6.48, 7.41, 7.45, 7.46, 7.49, and 7.53 in δ opioid, β2 adrenergic and the M2 muscarinic receptor. Some of these are present in the rhodopsin receptor (1.50, 2.50, 5.46, 6.40, 6.44, 7.41, 7.49, and 7.53). In the modeled receptor, most of the described residues involved in hydrogen bond network are well preserved. For most of these, interaction occurs in the presence of water in the inner part of the receptor and this cannot be reflected only by anhydrate models. Direct hydrogen bonds, however, were confirmed between N1.50, D2.50, N7.45, S7.46 and N7.49. In M. Babu *et al*. and based on the example of five receptors whose structures had been crystallized in the active and inactive form, researchers found repeated pairs of amino acids whose interactions were specific for each state of activation[43]. In inactive conformation the described interaction occurred between 3.46 and 6.37, 1.53 and 7.53, 7.53 and 8.50, and 7.54 and 8.51. These amino acids are located close to the amino acids forming a hydrophobic barrier and from the NPxxY motif both are connected to the Tyr toggle switch. Conformational changes associated with receptor activation entails the breaking of the above-mentioned effects and the formation of connections between 3.46 and 7.53 and 5.25 and 6.37. This observed phenomenon is supported by the results of mutagenesis studies[48,49]. During the visual analysis of selected models, we confirmed hydrophobic interactions between I3.46 and L3.37 and Y7.53, V1.53 and F8.50. An analogous arrangement was observed in both used templates. Upon visual inspection of models, the top 21 structures were selected for the second phase of assessment. All of these were generated by the Modeller program. 11 were built based on the rM3R template, while 10 others used mixed hH1R/rM3R templates. At this stage, this approach offers no obvious advantage over different algorithms used in the homology modeling program. Among the 21 selected models for further study there remained a similar number of structures generated by the same algorithm, i.e. 6 from automodel, 7 from MyLoop and 8 from loopmodel. A clear difference can be seen in the influence of the degree of refinement models on their final quality. Surprisingly, the greatest degree of refinement was the least favorable. Only one model with a slow large refinement option passed the first stage of selection. A clear improvement was observed using slow refinement. 12 models were developed in the next stage of the evaluation using a setting which gave a better result than the very fast or no refinement options. BCL::Score and QMEAN scores for selected models are presented in supplementary data S2 Fig.

### 3. Two-step docking assessment of models

**Fig 1:**
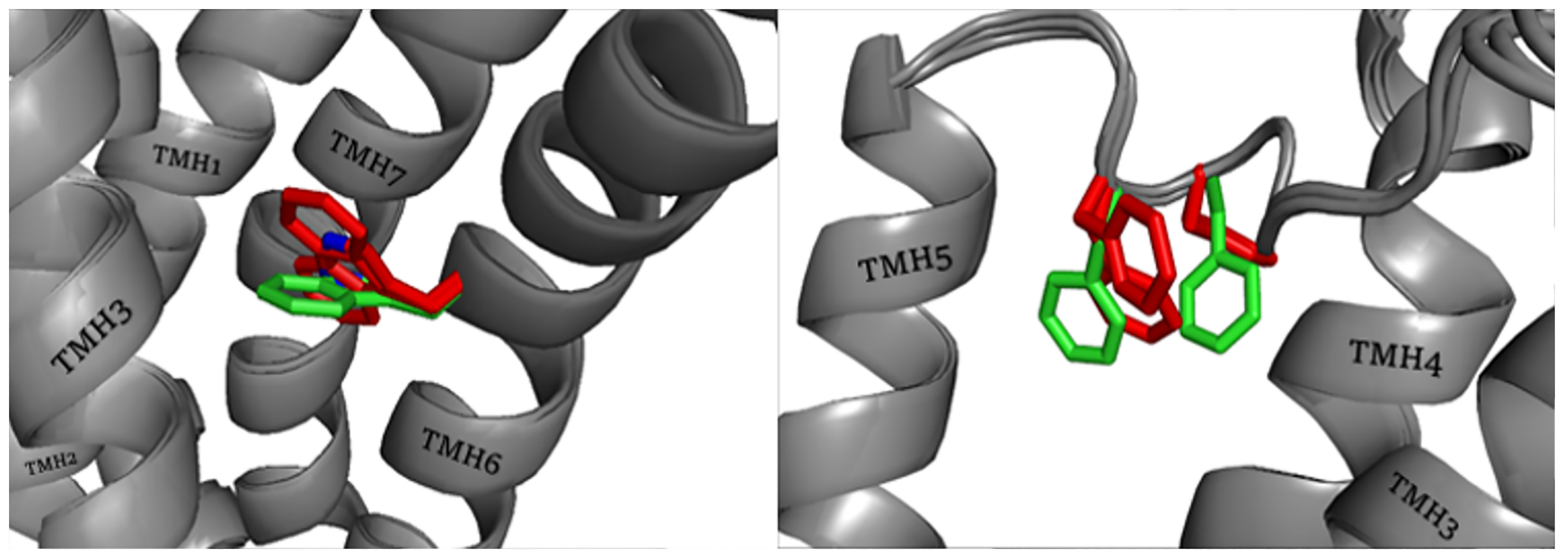
Position of W6.48 and ECL2 amino acids F45.54(F192) and F45.55(F193). Green color represents the conformation preferred by the ligands in docking with GOLD. Red represents the conformation from models where docking poses reach high RMSD values.

Docking procedure can be a good method for evaluation of the quality of homology models, in particular the quality of the ligand binding site[50]. In our studies, we use a two-step docking process. According to our previous studies, GOLD is characterized by high sensitivity to steric hindrance in the binding site, but it does not produce satisfactory results in reproducing the correct pose of the GPCR ligand compared to Glide. Consequently, GOLD was used in the initial verification and rapid selection of models with poor active site structure. Seven reference compounds were docked into active sites of modeled receptors. Selection of models was based on value of the ChemPLP scoring function, coherence of generated poses and visual inspection of proper pose orientation enabling binding to H3 receptor. A cutoff equal to 60 for ChemPLP function was established (supplementary data S3 Fig). At this point, problems in the ligand binding were noticeable for some of the models generated from multi-alignment with the help of loopmodel and MyLoop algorithms (Model code: BL00010001_10_5, BL00010001_10_6, BL00010001_10_9, BL00010001_12_5, BL00010001_12_6). In these models, the active site is severely limited by inwardly directed amino acid side chains. Ligands underwent wedging in the only free space between the TMH2, TMH3 and TMH7, below D3.32, although evaluation function results (reaching even negative values) indicate that this interaction should not be preferred by ligands. The ligand—receptor complexes were also analyzed for the convergence of docking runs. It was expected that if a receptor model possessed a good conformation then the ligand would adopt a preferable conformation easily, and docking runs would converge. On this basis, models that could not provide coherent results were rejected. Average root-mean-square deviation (RMSD) values between all poses of each docked compound are presented in supplementary data S4 Fig. There are no clear differences in the modeling parameters used, the chosen templates or BCL::Score and QMEAN values. Only the close view in the position of the individual residues led to the conclusion that the main difference in RMSD values between docking poses in various models corresponded with the position of W6.48 and two aromatic residues in ECL2, F45.54(F192) and F45.55(F193). In models B99990010_7_2, BL00010001_5_7, BL00010001_5_11, BL00020001_5_7, BL00020001_5_11, BL00030001_5_7 and BL00030001_5_11 in which docking poses reached the lowest value of RMSD in relation to the highest scored ligand conformation, these residues are very consistent. Differences between preferred and unpreferred conformation are presented in Fig 1.

**Fig 2:**
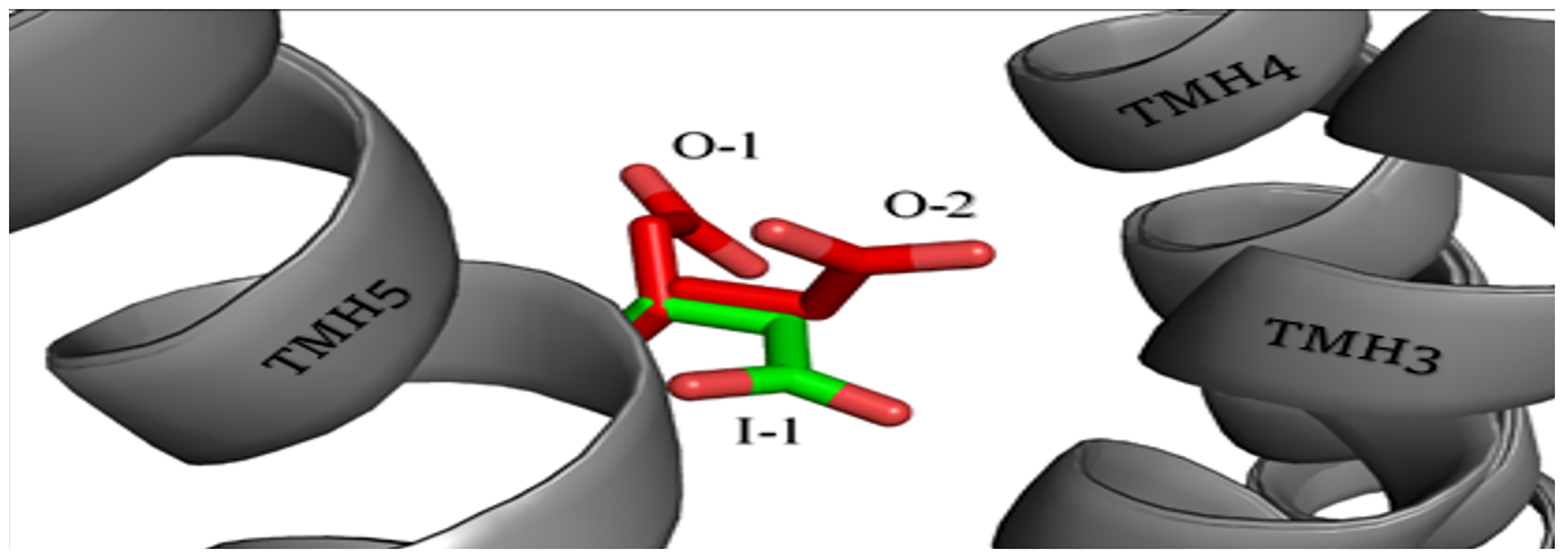
Three main conformations of the E5.46 observed during docking. Green color represents the conformation preferred by the ligands in docking. Red represents conformation from models where docking poses reach high RMSD values.

The final step in the evaluation of the docking results from GOLD was a visual inspection of the ligand-receptor binding mode. The most important element of the visual inspection was the selection of the common arrangement of ligand molecules inside the binding pocket and the analysis of hydrogen bonding and salt bridge formation with two binding amino acids important for histamine: D3.32 and E5.46. During the visual inspection of the binding mode, ligands docked outside the binding site, between transmembrane helices or in intracellular parts of receptor models were termed “unsuccessfully” docked. Models with mostly “unsuccessfully” docked ligands were rejected. Ligands placed within the orthosteric and/or allosteric sites are termed “successfully” docked. The number of “successfully” docked poses for each ligand for each receptor model are presented in supplementary data S5 Fig. During comparison of models we observed two kinds of docking artifacts. First, some ligand poses reached very high ChemPLP values, despite being located outside the binding pocket. Second, the earlier mentioned docking runs with all ligand poses located in the deep, intracellular region of the receptor were assessed as being very low according to ChemPLP scoring function. Both artifacts were most likely caused by an excessively tight binding pocket in some receptor models. Their occurrence shows how important it is to analyze the binding site precisely, even in models high-rated by other verification tools. The visual analysis of ligand poses was supported by literature data. In the A family of GPCRs the most important component of the monoaminergic ligand binding is a salt bridge between the protonated amine group of ligands and D3.32. D3.32 marks the border of the orthosteric active site. For most small molecules, the orthosteric active site is located deep between TMH3, TMH4, TMH5, TMH6 and TMH7. Some more complex ligands also interact with allosteric sites located in the extracellular part of the receptor. Site-directed mutagenesis experiments have demonstrated that amino acid residues in ECL2 are also important for binding of ligands to monoaminergic GPCRs[51,52]. The histamine H3 receptor has been the subject of only few mutagenesis studies. A. Uveges *et al*. carried out alanine scanning in TMH5 and described the effects of point mutation upon agonist binding[53]. They observed that mutation of residue D3.32 to either asparagine or glutamate resulted in a lack of mutant receptor activity. Alanine-scanning mutagenesis of 13 residues in helix 5 starting from W5.36 to T5.49, with the exception of A5.42 which mutated to glutamine. A 5-fold increase in potency was observed for W5.36A and T5.44A mutants, while the point mutations L5.39A, A5.42Q, E5.46A and F5.47A significantly reduced histamine potency (A5.42Q revealed 72% inhibition). For other agonists, similar effects were observed on the mutated receptors. Point mutation E5.46A had the greatest effect on the affinity of histamine and (R)-α-methylhistamine. This may indicate the importance of E5.46 blocked by antagonists or inverse agonists in the inhibition of receptor function. M.L. Jacobsen *et al*. described the mutation of three acidic amino acids to their amide derivatives: D3.32N, E4.65Q and E5.46Q[54]. Point mutation D3.32N led to the inhibition of activation of the receptor by the agonist [125I]-iodoproxyfan. Mutation E4.65Q was reported to be free of significant changes in receptor activity. The most interesting mutation, E5.46Q, resulted in a constitutively active receptor with a lack of capacity to bind (R)-α-methylhistamine and a 10-fold decreased binding of iodoproxyfan. Ciproxifan caused inhibition of the constitutive activity of the mutated receptor. B. Yao *et al*. mutated the amino acids T3.37 and A3.40 in human H3R to mimic corresponding residues in rat H3R[55]. Rat H3R exhibits greater sensitivity to some inhibitors, including the compounds A349821 and A304121. In the case of mutations T3.37A and A3.40V, receptors restore the high binding affinity of A304121 present in native rat receptors. It can be assumed that mutated amino acids participate in the binding of antagonists or affect conformation of other amino acids involved in ligand binding[55,56]. The results of directed-mutagenesis studies on the human histamine H4 receptor are also very helpful. This receptor has 48% amino acid identity with the human H3 histamine receptor. Identical amino acids within the binding site of both receptors, including E5.46, Y3.33 and Y6.51, are especially important due to their major impact on ligand binding. These amino acids increase the resemblance between binding sites hH3R and hH4R forcing similar conformations of ligands. This explains the number of ligands which are inhibitors of both of these targets. Research conducted by E. Istyastono *et al*. showed that the exchange E5.46Q weakened the binding strength of clobenpropit and its derivatives in both receptors[57]. S. Schultes *et al*. described mutations L5.39V and E5.46Q which caused a decrease in the activity of the tested ligands against histamine H4 receptors[58]. As a result of our analysis, we can confirm the importance of E5.46 in interactions with the antagonists we used. Among the models used in the GOLD docking, we observed three main conformations of the E5.46 side chain, presented in Fig 2 as O-1, O-2 and I-1. The location of the carboxyl group had a severe impact on the position of the imidazole fragment in the final results of the docking. The two outermost positions, directed "inward" (I-1 on Fig 2) or "outward" (O-1 and O-2 on Fig 2) to the center of receptor, gave the most consistent results. The "outward" positions seem to be inappropriate in terms of the hydrophobic region of the cell membrane in the direction of which polar residue of the E5.46 was directed. The biggest impact on the binding mode in the modeled receptor was shown by ECL2. ECL2 is the largest and the most diverse extracellular loop in class A GPCRs. ECL2 seems to be involved in ligand binding, selectivity and activation of GPCRs[59]. ECL2 is considered a stabilizer of the inactive state of the receptor[60]. However, ECL2 has been reported to participate in the constitutive activation of hM4R and hH4R[61,62]. In another mutagenesis study, D. Wifling *et al*. demonstrates a significant influence of the FF motif for ligand-receptor interaction and interconversion between inactive and active conformations of the receptor[52]. In our study, the position and arrangement of the loop was able to close access to the binding site. The arrangement of the FF motif in different model either creates an open space between the TMH4 and TMH5 or closes this region with aromatic residues. Each conformation of the FF motif had a serious impact on the final pose of the ligand. Only models in which F45.54(F192) and F45.55(F193) residues filled the space between the helices allowed us to obtain a consistent binding mode in the predicted binding space (green conformation in Fig 1). The next important amino acid which allows ligands to bind to the active space of the receptor is the previously mentioned W6.48. W6.48 appeared in two positions: parallel or perpendicular to the axis of helix arrangement. Parallel position led to a significant reduction in the volume of the binding pocket, in particular affecting the position of thioperamide and clobenpropit. Perpendicular position improved the interaction of the protonated amine group with E5.46. This is also the preferred conformation for imidazole ring recognition by this region.

**Fig 3:**
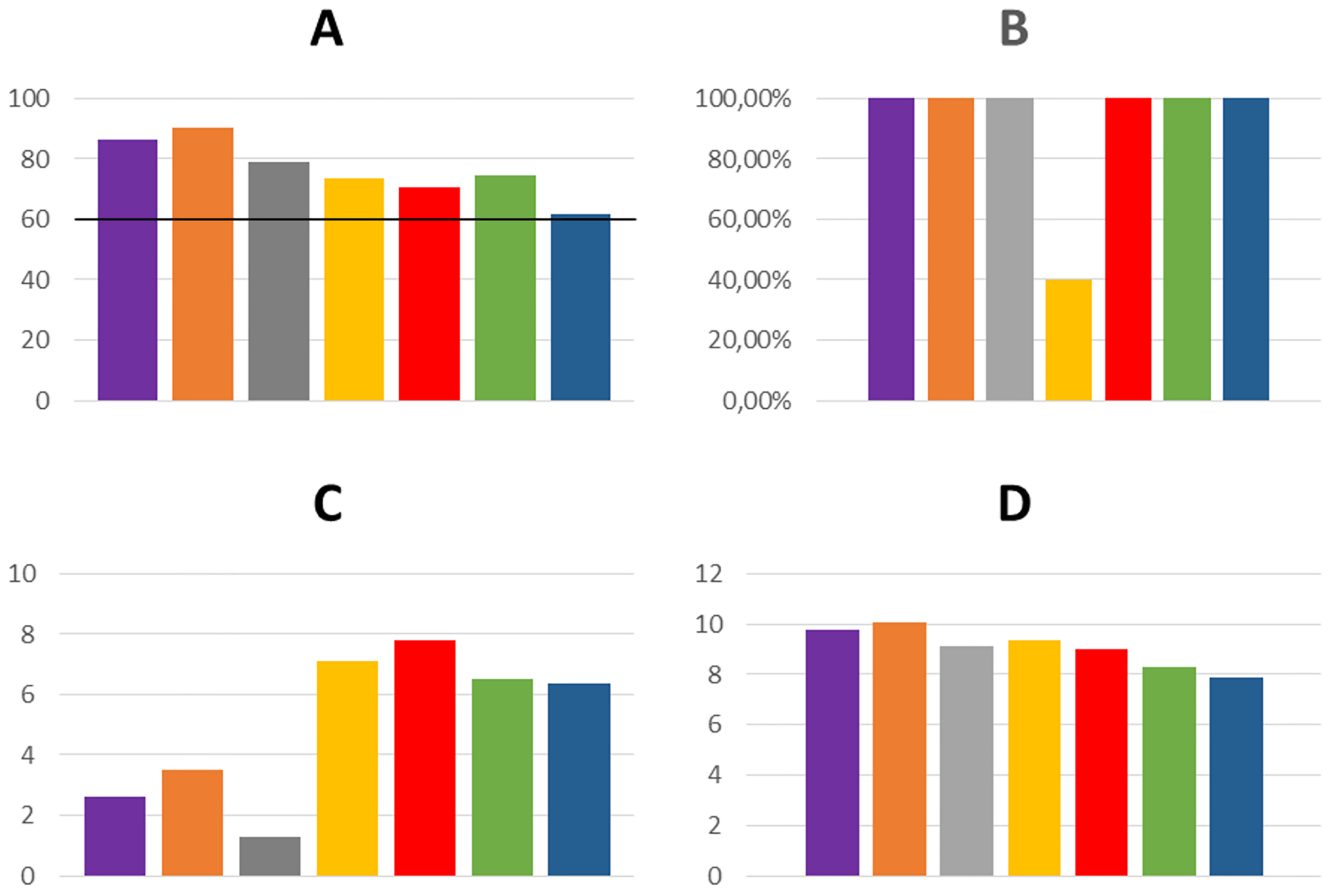
Results of ligand-based docking assessment for the best generated model. Docking with GOLD, presented as a ChemPLP score for the best pose of each ligand (A) with a cutoff score line, the percentage of ligand pose located in the active site of the H3 receptor after docking with GOLD (B). Average RMSD values across all poses of each ligand in each docking. Ligands compared to best scored pose (C). Absolute GlideScore values for the best ligand pose (D). Colors represent the individual ligands: A331440 –purple, A349821 –orange, ABT 239 –gray, Ciproxifan—yellow, Clobenpropit—red, JNJ520785 –green, Thioperamide—blue.

Five models of the H3 receptor that passed the ligand-based selection with GOLD were chosen for the second step of docking, which involved extra-precision docking in Glide. The assessment was based on two criteria: GlideScore value; and analysis of ligand binding mode. Values of the GlideScore function for the best conformation of each ligand in all models participating in the second step of docking are shown in supplementary data S6 Fig. In only one receptor model, B99990010_7_2, did ligands have GlideScore absolute values mostly higher than 8. Visual analysis of ligand binding mode proceeded according to the same criteria as in the case of docking with the GOLD program. The key element of the analysis was the assessment of imidazole and protonated amine position. The first possible acidic grip point is D3.32, which can bind the protonated amine and is essential for interaction between agonists, including histamine, and histamine receptors[63,64]. The second acidic grip point is created by E5.46 and the accompanying tyrosines Y3.33 and Y6.51. According to literature reports describing histamine H1 receptors, these amino acids are equivalent to N5.46, Y3.33 and Y6.51, which are responsible for binding with the imidazole ring of histamine[63,64]. The vast majority of obtained poses, especially those receiving high GlideScore values, have a grip point for protonated amines or imidazole in the region near E5.46, Y3.33 and Y6.51. Again, an important role in the arrangement of the ligands is played by the position of the free carboxyl group from E5.46 which can adopt a position directed inward or outward to the center of the receptor (Fig 2). Among the models used in Glide docking, the inward conformation of E5.46 occurred only on B9990010_7_2, while in four other receptors there was an outward arrangement. For ligands ABT239, A331440, A349821 and JNJ5207852, the arrangement of ligands was the same in all tested models. Protonated amines of those ligands consequently bind to E5.46, Y3.33 and Y6.51. There was a clear enhancement in binding scores in the case of the inward directed position of E5.46 in the B9990010_7_2 model. In comparison to all the inhibitors used in the study, clobenpropit with its imidazole ring and isothiourea fragments, both able to accept protons, use both acidic grip points in the ligand binding space. This causes a unique arrangement of clobenpropit in the receptor. In B9990010_7_2, isothiourea interacts with E5.46 which forces the imidazole ring to create an arrangement near D3.32. When, in other tested models, the carboxyl group of E5.46 is directed outwardly, the imidazole ring reaches D2.50 which is located under D3.32. GlideScores assessed both conformations similarly, but a second arrangement of the ligands occurred during the first stage of docking with GOLD; however, it was rated extremely low. This mismatch between the scoring results and the literature data of E. Istyastono *et al*. and S. Schultes *et al*. on the histamine H4 receptor, where clobenpropit presents a conformation close to that established in the B9990010_7_2 model, casts doubt on the possibility of this kind of ligand arrangement in the active site[57,58]. On this basis, we chose the interaction with B9990010_7_2 as being more reliable. The most diverse conformations were obtained for thioperamid and ciproxifan, which are relatively small ligands. Ciproxifan obtained a vertical conformation. This was alongside TMH5, which interacted with the ECL2 loop and E5.46, Y3.33, Y6.51 residues only in the B9990010_7_2 model. In the other four models, a planar arrangement with an imidazole fragment interacting with Y3.33 and Y6.51 was more common. The cyclopropane fragments were close to D3.32. The best horizontal poses were still scored lower than vertical ones obtained in the first model. On the other hand, in the case of thioperamid two coherent binding modes also occurred. Here, the planar conformation where protonated imidazole interacted with D3.32 and thiourea was near E5.46, and this was rated higher by GlideScore. In the case of this ligand, the external position of the E5.46 carboxyl group in the BL00010001_5_7, BL00010001_5_11, BL00020001_5_7, BL00030001_5_7 models allows a better fit of the ligand due to the larger share of hydrophobic interaction. Analysis of the ligand binding mode in conjunction with a clear diversification of the GlideScore values shows that the best model, allowing reliable examination of ligand receptor interactions, is the B9990010_7_2 model and therefore, this was used in subsequent steps of the studies. Score values for each of the steps of the B9990010_7_2 are shown in Fig 3. The alignment used to prepare the best model is shown in S1 Fig in the supplementary data.

### 4. Interactions between inverse agonists / antagonists and the human histamine H3 receptor

**Fig 4:**
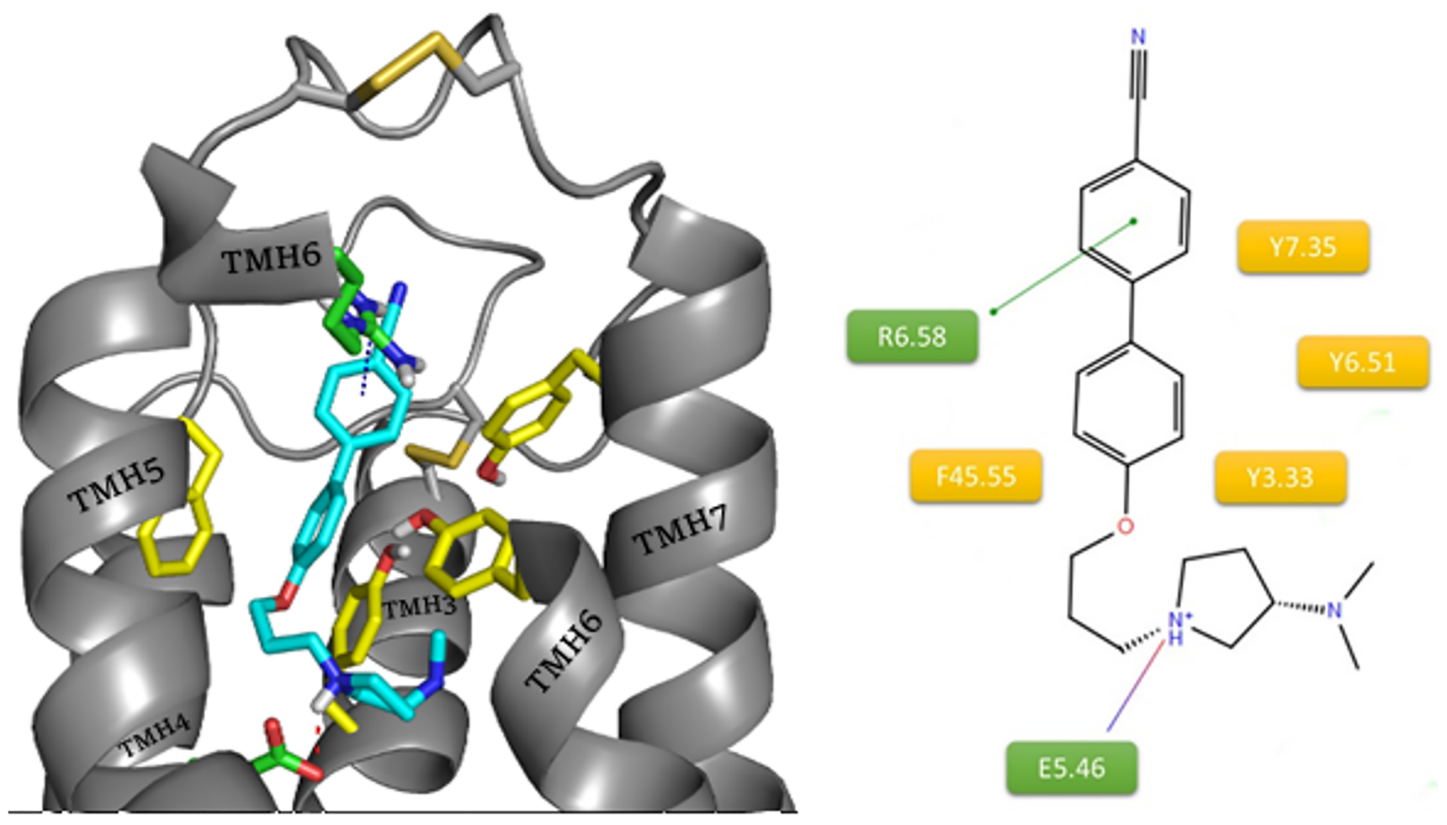
Binding mode of compound A331440 (blue) within the histamine H3 receptor. The amino acids involved in the non-polar interactions are highlighted in yellow. Amino acids involved in polar interactions (green line: cation– π, purple dash: salt bridge) are marked in green.

The final model of the histamine H3 receptor and results from XP Glide docking were used to analyze the binding mode for the whole set of reference ligands. Among the 7 ligands used in the docking study, A331440, A349821 and ABT 239 formed a coherent group of structurally related ligands with the same binding mode. The consistent and a repetitive interaction motif they exhibited was determined by three structural elements: a protonated amine in the pyrrolidine ring; an oxygen atom in the linker; and a biphenyl fragment. The first two elements interacted within the orthosteric binding site. The protonated amine was involved in the salt bridge with E5.46. The position of the linker oxygen seemed significant as the acceptor of a potential hydrogen bond with the Y3.33 and Y6.51 in the presence of water. The last element, the aromatic ring connected to the oxygen atom, created hydrophobic interactions with F45.55(F193) on the edge of the allosteric binding site of the receptor. The second aromatic ring formed cation– π interactions with R6.58 and CH– π with Y7.35. The described binding mode of A331440 is presented in Fig 4.

**Fig 5:**
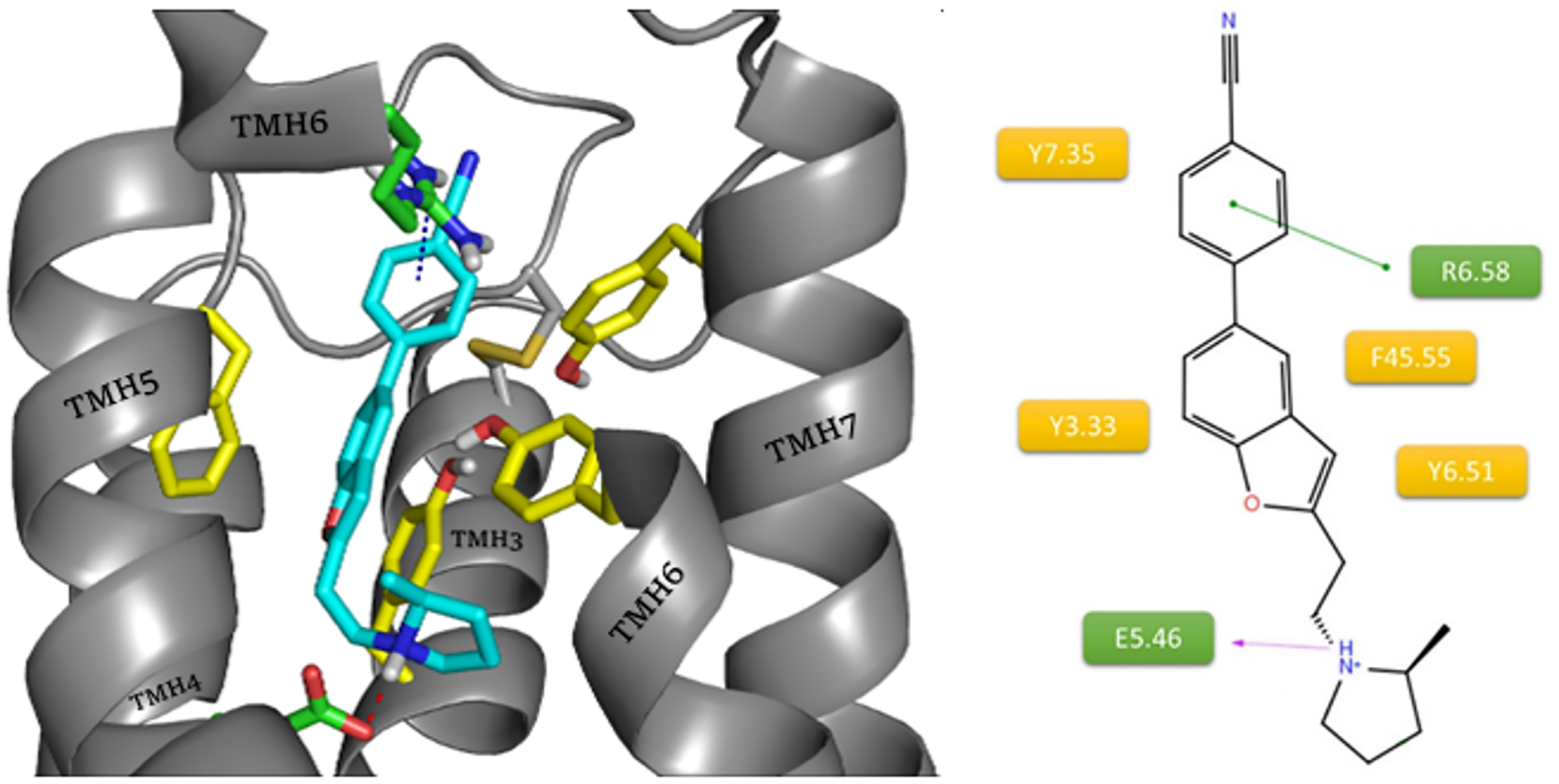
Binding mode of compound ABT 239 (blue) within the histamine H3 receptor. The amino acids involved in the non-polar interactions are highlighted in yellow. Amino acids involved in polar interactions (green line: cation– π, purple arrow: salt bridge) are marked in green.

Cyclization of linkers leading to the creation of benzofuran stiffens the ABT 239 molecule (Fig 5). The larger aromatic system additionally enhances the interaction with ECL2 amino acid F45.55(F193). In binding modes of both A331440 and ABT 239 the distance between protonated amine and oxygen remains constant.

**Fig 6:**
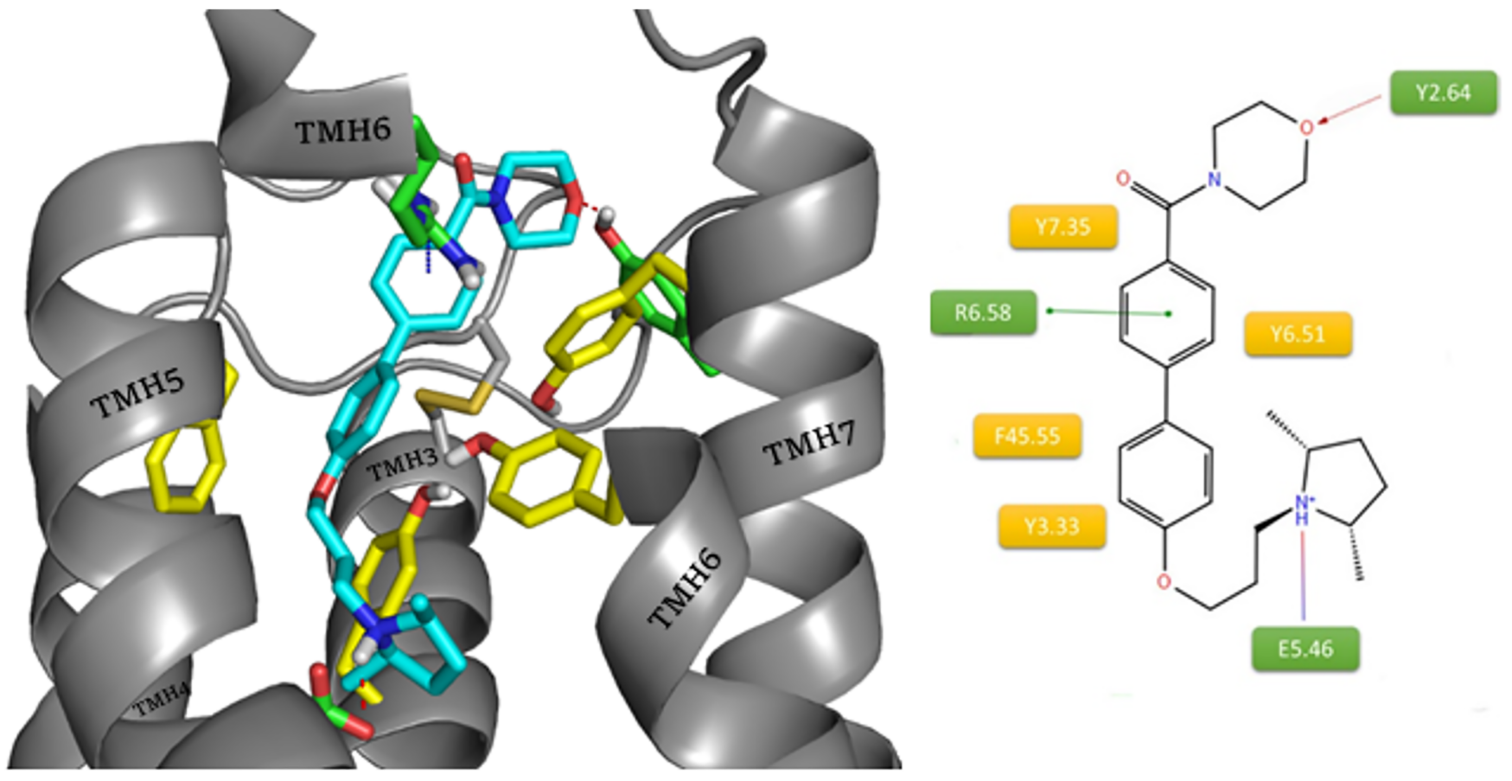
Binding mode of compound A349821 (blue) within the histamine H3 receptor. The amino acids involved in the non-polar interactions are highlighted in yellow. Amino acids involved in polar interactions (red arrow: hydrogen bond, green line: cation– π, purple dash: salt bridge) are marked in green.

In the case of A349821 (Fig 6), the nitrile substituent was changed to morpholine. Use of the amide connection between the aromatic ring and heterocycle enhanced the binding of the ligand to the allosteric binding site *via* an additional hydrogen bond with Y2.64.

**Fig 7:**
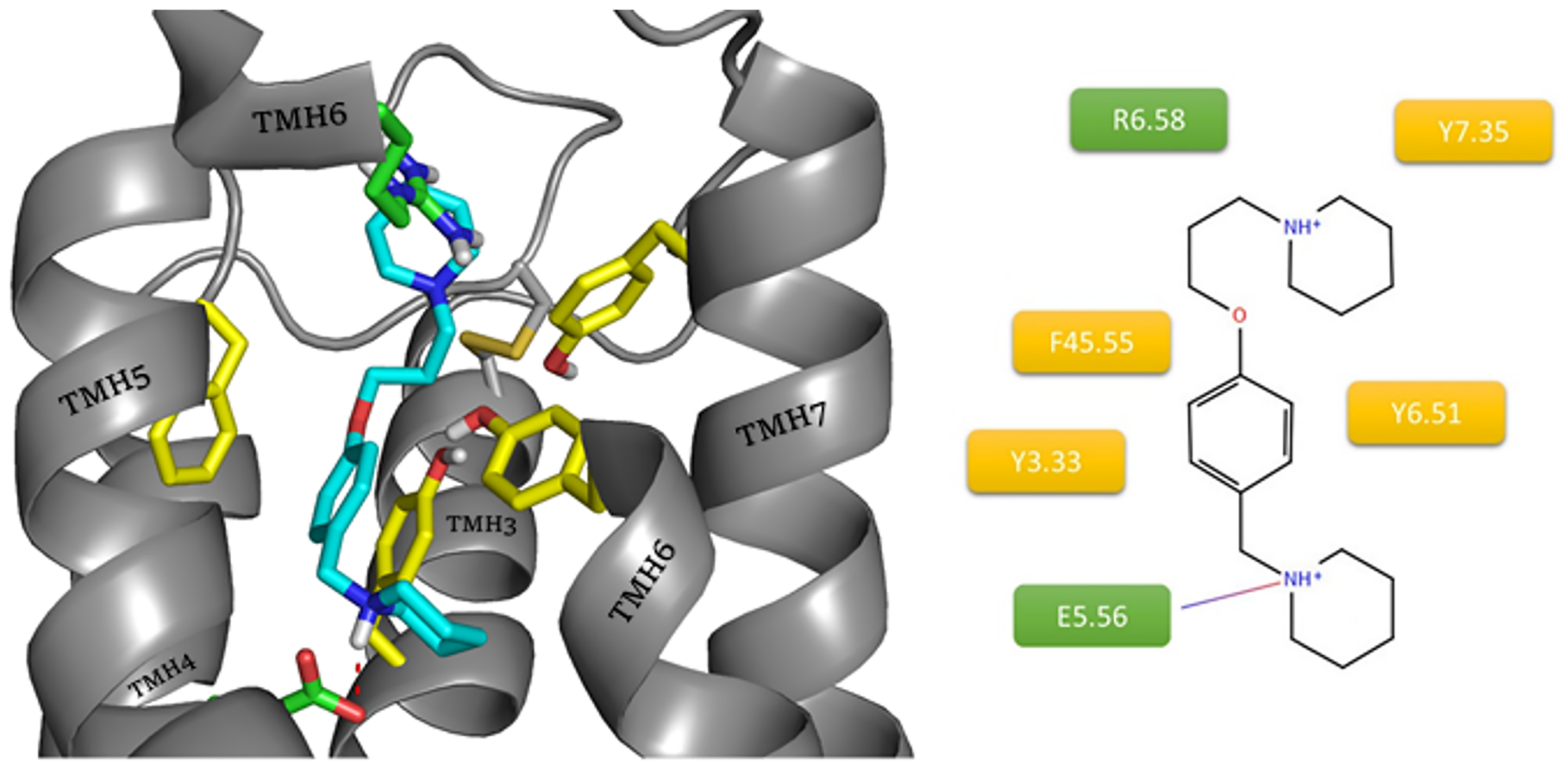
Binding mode of compound JNJ5207852 within the histamine H3 receptor. The amino acids involved in the non-polar interactions are highlighted in yellow. Amino acids involved in polar interactions (red arrow: hydrogen bond, green line: cation– π, purple dashes: salt bridge) are marked in green.

JNJ5207852 presented a similar interaction motif, but in this case there are two charged nitrogen atoms in piperidine rings (Fig 7). The presence of the protonated amine and an oxygen atom in the linker maintained the interactions with E5.46, Y3.33 and Y6.51 in the orthosteric site. The second nitrogen atom could be involved in hydrogen bonding with the ECL2 main chain (Y45.51(Y189), A45.52(A190)) and other amino acids in the allosteric site (R6.58). The presence of a motif known from previous ligands, an oxygen atom in the linker 3 carbon atoms away from the protonated amine, argues for a reverse arrangement of the ligand. The absence of the second aromatic ring responsible for interactions within the allosteric site which was present in the earlier described ligands, as well as the presence of two amines with equal protonation chances, made the described conformation much more common and more highly scored within docking results. This suggests that such an aromatic ring position can be more beneficial for the interactions with the receptor.

**Fig 8:**
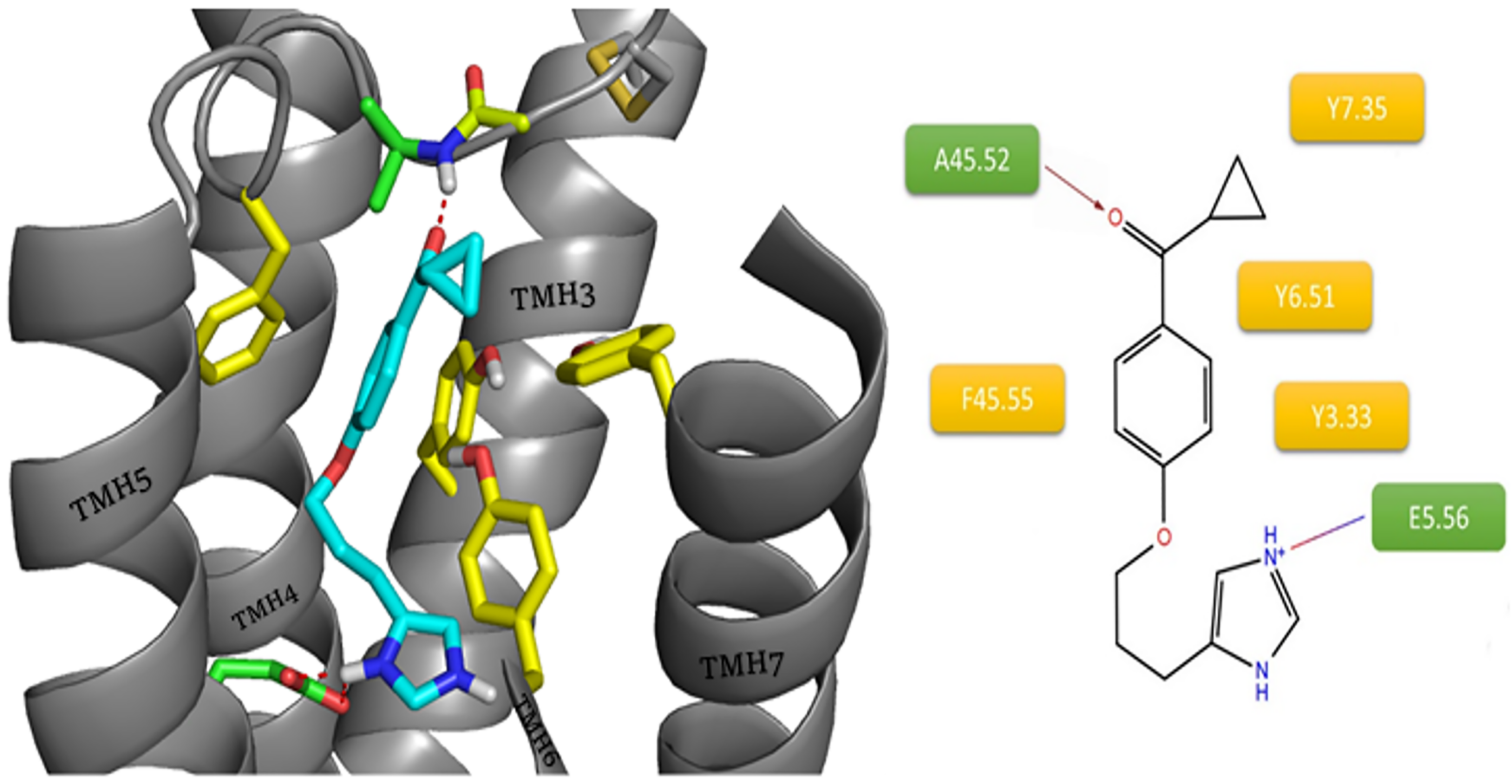
Binding mode of ciproxifan within the histamine H3 receptor. The amino acids involved in the non-polar interactions are highlighted in yellow. Amino acids involved in polar interactions (red arrow: hydrogen bond, green line: cation– π, purple dashes: salt bridge) are marked in green.

Different situations were observed among inverse agonists containing imidazole rings in their structure: ciproxifan, clobenpropit and thioperamide. The first, ciproxifan, preserved the orientation characteristic for compounds with protonated amines. Imidazole rings of inverse agonists occupied regions indicated as interacting with heterocyclic rings of the natural agonist, histamine. As before, the oxygen atom from the linker was close to Y3.33 and Y6.51, enabling hydrogen bonds with those amino acids, most likely with the participation of water molecules. The carbonyl group present in the molecule allowed for the creation of a hydrogen bond with the ECL2 main chain (Y45.51(Y189), A45.52(F190)). Aromatic rings, as in previous ligands, interact with Y3.33 and Y6.51 and F45.55(F193) from ECL2 (Fig 8).

**Fig 9:**
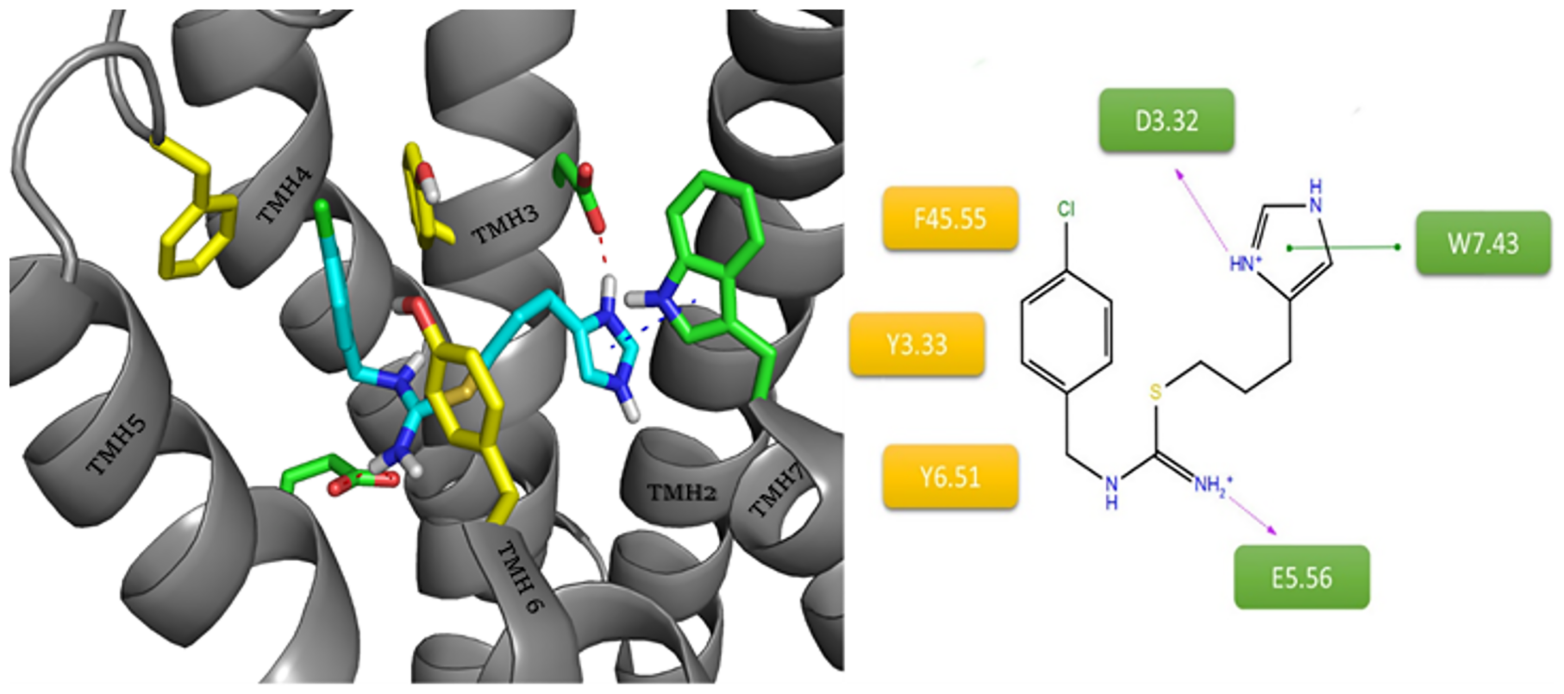
Binding mode of clobenpropit within the histamine H3 receptor. The amino acids involved in the non-polar interactions are highlighted in yellow. Amino acids involved in polar interactions (green line: cation– π, purple dashes: salt bridge) are marked in green.

Another imidazole ligand, clobenpropit, revealed different binding mode with histamine H3 receptor in comparison with ciproxifan and a ligands with a cyclic amine (Fig 9). A protonated thiourea fragment provided ionic interaction with E5.46. This salt bridge promoted arrangement of imidazole near D3.32 with the creation of a second salt bridge and cation– π interactions with aromatic W7.43. These polar interactions were complemented by a chlorophenyl substituent interacting with the aromatic rings of F45.55, Y3.33 and Y6.51. This arrangement strongly binds ligand within the orthosteric binding site, limiting interaction with the allosteric site of the receptor.

**Fig 10:**
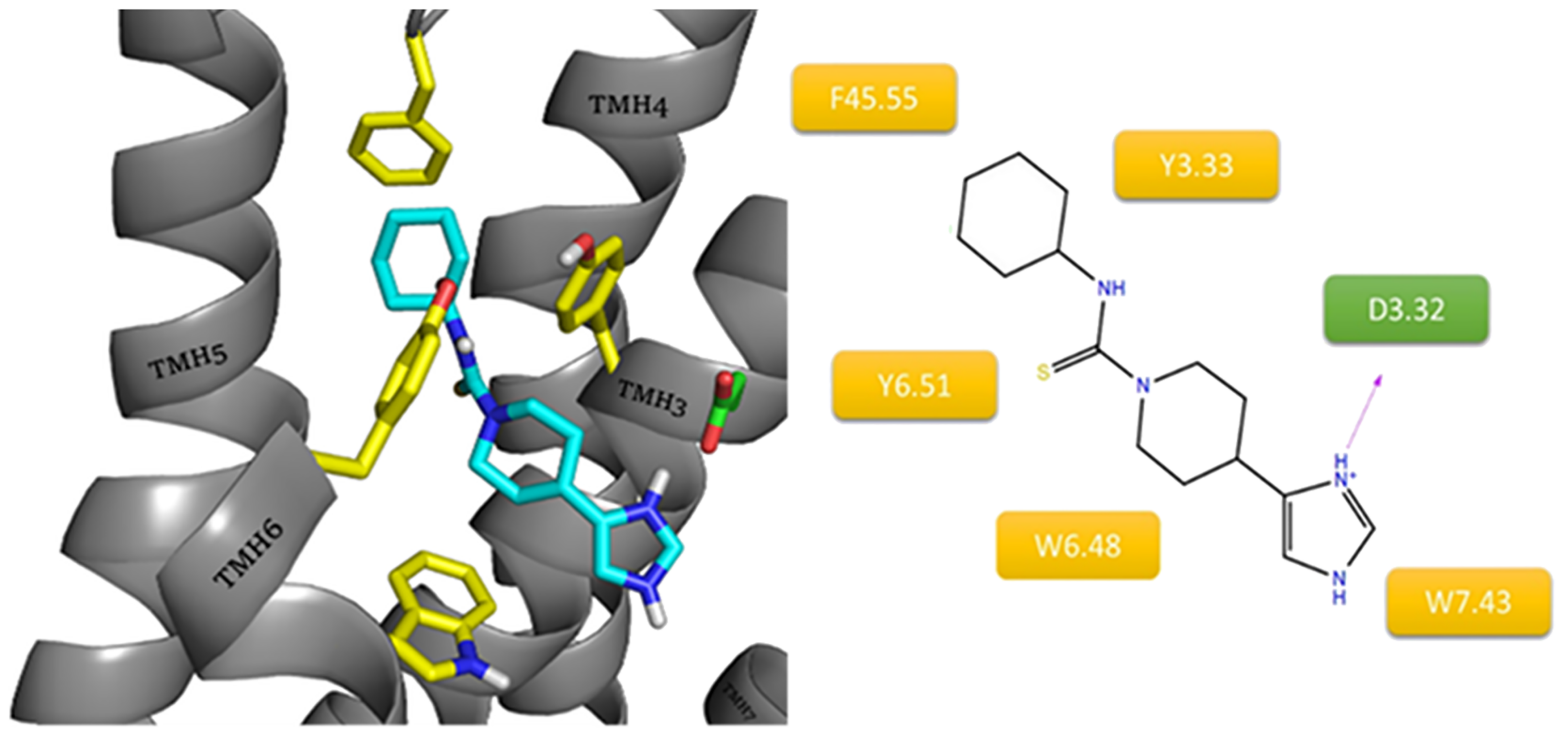
Binding mode of thioperamide within the histamine H3 receptor. The amino acids involved in the non-polar interactions are highlighted in yellow. Amino acids involved in polar interactions (purple dashes: salt bridge) are marked in green.

Similarly to ciproxifan, the last inverse agonist, thioperamide, is devoid of amine groups able to easily accept protons, other than that at the imidazole ring. The main difference between these two compounds is their conformational freedom. In contrast to ciproxifan, thioperamide is a compound with a relatively rigid structure. During the docking, this “rigidity” was translated into large differences in the ligand position (Fig 10). This small ligand was able to arrange along the orthosteric binding site. As with clobenpropit, the imidazole ring creates a salt bridge with D3.32. The different conformation of thiourea in thioperamide blocks protonation and thus prevents interaction with E5.46. This was reflected in the higher docking scores for similar thioperamide conformations in other H3 models in which the carboxyl group is directed outwardly. The receptor-ligand system was stabilized via a series of hydrophobic interactions between cyclohexane and aromatic residues F45.55(F193), Y6.51 and Y3.33, which are usually involved in the binding of the aromatic fragments of ligands.

### 5. Comparison of the results with literature data

As indicated above, many scientific groups have attempted to create a homology model for the histamine H3 receptor to explain how ligands bind or for drug design and virtual screening[34,65–69]. We decided to compare the results obtained in our study with those of previously published studies. The position of the putative binding site identified by the analyzed studies was always very similar. B. Rai *et al*. used the G model protocol with profile-based alignment and experimental restraints for model refinement. Just as in our study, the authors used the well-known inverse agonists of H3 receptor: ciproxifan, clobenpropit and thioperamide[65]. In their presented binding mode, compounds were associated with D3.32 by protonated imidazole system. The tail parts of the compounds cyclopropane, cyclohexane or chlorobenzyl interacted with T3.37 and/or A3.40. In this way, B. Yao *et al* tried to explain the effect of mutations in these positions on H3 inhibitor affinity[55]. In the case of clobenpropit, in addition to the imidazole ring, the compound has a second nitrogen in the fragment of the isothiourea that can protonate. In the described model, isothiourea is involved in the formation of a hydrogen bond with T6.52. Another interesting feature of the proposed binding mode for clobenpropit was the formation of a hydrogen bond between the benzyl aromatic ring and hydrogen atom of the hydroxyl group at T3.37. The binding mode presented in our research is more consistent with the studies of A. Uveges *et al*. and M. L. Jacobsen *et al*. in which E5.46 shows a very large impact on ligand binding efficiency[53,54]. Ligands docked to our model do not have a direct way to interact with T3.37 and A3.40. At this point, we assumed from the obtained mutation results that T3.37A and A3.40V affect the binding of ligands by E5.46, by improving its adjustment to fragments interacting with that residue. E5.46 is an amino acid which has a relatively high conformational flexibility. During our analysis of homology models, we observed a preferred position of the free carboxyl group of E5.46 which can be easily achieved by the receptors with mutated amino acids T3.37A and A3.40V. In the model presented by B. Rai *et al*., a E5.46 carboxyl group appears to be located between TMH3 and TMH5; and thus, its interaction with protonated amines is limited. A clear difference between proposed models is also the position of ECL2. In our study, ECL2 appears to have a significant impact on the position of the ligand as well as on access to the binding site. The model presented by the research group from Wyeth Research has a loop which clearly closes the orthosteric binding site of H3 receptor. Moreover, B. Rai *et al*. indicate that aromatic residues such as Y45.51(Y189) are directed toward the binding sites which causes a further limitation to the space available for the ligands. In our model, ECL2 leaves a lot of space within orthosteric binding sites. Y45.51(Y189) is directed to the outside, enabling large and rigid fragments, such as biphenyl in A331440 and A349821, to fit in. Also very important in our study is the fact that F45.54(F192) and F45.55(F193) are crucial for the correct positioning of the ligand. F45.55(F193) with Y3.33 and Y6.51 play a special role in the binding of H3 ligand aromatic fragments, among others chlorophenyl in clobenpropit. Another model was proposed by F. Ax *et al*[34]. They presented an hH3R model built on bovine rhodopsin (PDB: 1F88) as a template. To generate the model, an earlier version of the Modeller program was used. The generated models were optimized in the presence of a membrane with the CHARMM program. The common element of both studies is the use of JNJ5207852 for the analysis of ligand receptor interactions. In this rhodopsin based model, ligands are arranged horizontally along the allosteric binding site. The protonated amine in the benzylpiperidine fragment creates a salt bridge with E5.46. A second positively charged nitrogen of piperidine interacts in the same way with D3.32. These interactions are complemented by aromatic ring interactions with residues Y3.33 and W6.48. For such a ligand arrangement, the conformation of the indole ring W6.48 should be parallel to the axis of the TMH6. This gives adequate space for the horizontal position of the ligand. In our model, L7.42 does not allow such conformational freedom to W6.48. Consequently, compound JNJ5207852 adopted a vertical arrangement during docking to our model and bound to both an allosteric site (within the E5.46) and an orthosteric one (R6.58 and Y7.35). In both modes, you can see some common features. In both receptor models, that proposed by F. Ax *et al*. and ours, a free carboxyl group of E5.46 is facing toward the center of the receptor. Whatever the final arrangement of the benzylpiperidine fragment, it interacts with this particular amino acid. Another similarity can be found in the interaction between the aromatic ring and Y3.33; however, in our model this directs the ligand toward the orthosteric site of hH3R. S.K. Kim *et al*. proposed a model of the histamine H3 receptor based on the human β2 adrenergic receptor (PDB: 2RH1) built using the GEnSeMBLE (GPCR Ensemble of Membrane Structures in bi-Environment) method[67]. An interaction study was conducted for a variety of antagonists and inverse agonists of the H3 receptor by using the Darwin Dock protocol. Among these clobenpropit, ABT 239, ciproxifan and thioperamide were also used in the analysis of the model presented here. Clobenpropit bound to the described model hH3R with the contribution of Y3.33, W7.43 and D3.32. The isothiourea group created a polar interaction with D3.32 and Y6.51. The imidazole ring formed an additional H-bond with the E5.46. Predicted interactions of the clobenpropit benzyl group take place within hydrophobic residues L7.42 and W7.43. This is the opposite arrangement to that provided by our model, in which imidazole interacts with D3.32. The isothiourea group, as a second protonated system, created a salt bridge with E5.46. The benzyl fragment enters into interaction with aromatic Y3.33 and F45.55(F193). In the case of ciproxifan, the model built on human β2 adrenergic receptor provides a similar arrangement to clobenpropit. Imidazole interacts with E5.46; however, the aromatic fragment is directed toward the F7.39, and the cyclopropyl substituent is directed up to Y2.61. In our model, ciproxifan shows a vertical orientation wherein imidazole binds to E5.46, but the aromatic moiety binds to Y3.33 F45.55(F193). The carbonyl group forms a hydrogen bond with the main chain of ECL2 (A45.52). The most significant differences between the models seem to be related to the conformation of residues E5.46, W6.48 and Y3.33. For example, Y3.33 and E5.46, as presented by the SK. Kim *et al*. model, often assume conformations in which their side chains are directed into the space between the TMH4 and TMH5. In crystals of receptors more closely related to hH3H (hH1R, rM3R, hM4R) than the hβ2AR, hydroxyl groups of Y3.33 and Y6.51 are directed toward each other in the central part of the receptor. The differences in W7.43 conformation between models may be another cause of the different positioning of clobenpropit. Other compounds with a 4-(cyclopropanecarbonyl)phenoxy fragment presented by SK. Kim *et al*. were arranged in a different manner than that shown by ciproxifan. This different position is much closer to the arrangement of this fragment in our model. Another compound described by SK. Kim *et al*. is thioperamide, which is arranged in a very similar manner to that described in this study. In both cases, the ionized imidazole binds to D3.32 *via* the salt bridge. The molecule itself is arranged alongside the allosteric site. The terminal cyclohexane ring interacts with an aliphatic L5.39 and aromatic ring of F45.55(F193). The thiourea motif in both cases is located between Y3.33 and Y6.51 side chains. ABT 239 was the last ligand tested in both models. SK. Kim *et al*. described a binding mode wherein the protonated amine of pyrrolidine creates a salt bridge with D3.32. Further, aromatic fragments interact with the hydrophilic or lipophilic groups within lipophilic pockets created by amino acids from TMH 3–5–6. The binding mode presented in this way does not show many specific interactions, and only slightly explains the selectivity of the ligand against H3R. In our model, ABT 239 as well as other ligands with similar structure (A331440, A349821) arranged vertically along the TMH5. A protonated amine, as a substitute for the imidazole ring, interacts with E5.46. Benzofuran interacts with F45.55(F193), Y3.33 and Y6.51. The terminal aromatic system interacts within the orthostatic binding site, and creates interactions: aromatic with Y7.35 and cation– π with R6.58. The issue of the correct prediction of ligand binding mode in H3 receptor models has been also investigated by N. Levoin *et al*. based on two hH3R models[68,69]. First, where the human H3 receptor sequence (Q9Y5N1) was aligned with bovine rhodopsin (PDB: 1L9H) as a template. Second, where the same sequence was aligned with hH1R (PDB: 3RZE). In both cases N. Levoin *et al*. observed diversification in the obtained binding modes for a diverse group of described ligands. However, in both cases the most frequent dominant binding mode was proposed. N. Levoin *et al*. foresees the creation of the salt bridge between the protonated amine in ligand and E5.64. Further, ligands are in contact with Y3.33, Y6.51, F5.31, mostly by aromatic fragments and oxygen atoms in the linker. In the case of dibasic ligands similar to JNJ5207852, E45.37(E175) or E45.53(F185) were indicated as being involved in the creation of a second salt bridge. These observations are very consistent with the dominant binding mode presented by A331440, A349821, ABT 239 and JNJ5207852 during our study. Despite the many similarities between the binding mode presented in this article and the binding mode described by N. Levoin *et al*., there are several significant differences. In the binding modes presented here, F5.31 does not participate in ligand receptor interaction. The position of F5.31 on the TMH5 determines the position of the side-chain beyond the binding site of the receptor. Also, E45.37(E175) and E45.53(F185) were not indicated as amino acids participating in the binding due to their very limited contact with the ligands in the active space. These limitations result from our proposed conformation of ECL2. The position of E45.37(E175) and E45.53(F185) were strongly determined by the positions of equivalent amino acids, i.e. Q4.65 or Q45.53, in the template. However, great freedom within ECL2 in the GPCR receptor family is well known. This could lead to conformational changes which allow the formation of interactions between one of the mentioned glutamic acid residues and the ligand. In a recent study conducted by K. Kuder *et al*., compounds based on the structure of pitolisant were presented[66]. Binding modes of those ligands were analyzed using a homology model of the H3 receptor prepared with the internet service GPCRM based on the model of M3 muscarinic receptor (PDB: 4DAJ) as a template. The described compounds have two marginally situated fragments connected with an alkyl linker responsible for binding to the receptor. The first fragment was heterocyclic system: piperidine, methyl piperidine or azepane; the second one was aromatic ring of phenol derivative substituted with chlorine. Again, the binding mode presented for these antagonists involves the creation of a salt bridge between E5.46 and the protonated amine from the heterocyclic system. Further, compounds were arranged vertically toward the orthosteric binding site where the aromatic system interacted with Y45.51 in ECL2. Mono-protonated compounds with a similar structure, such as ciproxifan or ABT 239 also in our model adopt a very similar binding mode. The protonated system was associated with E5.46 and the same compound arranges an aromatic system toward ECL2. In our model, however, there was far more substantial interaction with F45.55(F193) which, as described previously, corresponds to the research by D. Wifling *et al*. indicating the importance of this amino acid in the ligand binding[52].

### 6. Virtual screening performance test

Considering the potential use of the described model in virtual screening, we performed preliminary tests to check the model's performance in search of active compounds among non-active decoys. For this purpose, from GLL database we downloaded a library of H3 antagonists with proved activity against the histamine H3 receptor and a library of specific H3 decoys from GDD[70]. The library used for virtual screening included 3000 compounds and was prepared in KNIME[71] by which 300 active ligands and 2700 decoys were randomly selected and tagged with “Active” and “Decoy” marks. Virtual screening has been conducted in an analogous way to the selection process of the best homology model. In the first stage of screening, we docked compounds with GOLD program to the binding site of the histamine H3 receptor [72]. Docking parameters and the size of binding site were unchanged. Potentially active compounds have been identified as ligands that have been docked in the inner part of the receptor, within the described active site and have received the ChemPLP function value at least 60. Such ChemPLP score was a cutoff value at an earlier stage of the study. After this stage 1438 compounds were rejected. Further, the remaining 1562 ligands were docked with Glide using XP algorithm[73]. The final selection was based on GlideScore value. Ligands with absolute GlideScore values 7.9 or more have been identified as potentially active. Finally, over 489 ligands passed the screening. Among them 95 active and 394 decoys were found. Most of the active compounds were at the top of ranking list. Such procedure seemed to work partially, therefore, we tried to rescore the ligand poses received after glide docking with other evaluation functions. We have selected two: ASP from GOLD program and the available online DrugScoreX (DSX)[74]. After that we received 673 ligands with ASP score above 43.66 (this is ASP score for Tioperamid in conformation described earlier), 138 active and 535 decoys. After DSX rescoring 1505 ligands remained with absolute score 80 or more (80 is DSX score of Tioperamid in conformation described earlier). Among them 209 active and 1295 decoys were found. These results are still not satisfactory, but they show how the evaluation function influence the effectiveness of virtual screening. In future research we’ll try to choose the most effective assessment method using described here model to improve screening accuracy.

## Material and methods

For GPCR residue identification, the Ballesteros-Weinstein nomenclature was used[75]. All visualizations were performed using PyMol 0.99rc6[76] and Maestro 10.6[77] tools.

### 1. Sequence alignment

The sequence of the human histamine H3 receptor was obtained from the Universal Protein Resource (UniProt Entry: Q9Y5N1)[78]. Information about receptor structures, sequence similarity and mutagenesis information collected in the GPCRdb database were used to choose the best template[79]. The sequences of crystallized receptors available in the Protein Data Bank (PDB) database were compared within all the helices and the second extracellular loop (ECL2). These structures are mainly responsible for ligand binding to the GPCR A family receptors. Matrices with the largest number of identical (I%) and the similar (S%) amino acids were selected for sequence alignment with the human H3 receptor. These were the human histamine H1 receptor (hH1R) (I% = 30%, S% = 56%) and rat M3 muscarinic receptor (rM3R) (I% = 31%, S% = 54%). The sequences and 3D structures of templates, *i*.*e*. the rat muscarinic M3 and human histamine H1 receptors, were obtained from the PDB[80]. Both of the reference receptors were bounded in complexes with antagonists, preserving inactive spatial conformations. The T4-lysozyme insertions in templates, as well as the first 34 from the N-terminus and 20 amino acids from the C-terminus were removed. The third intracellular loop (ICL3) was not modeled. For sequence alignment, Clustal Omega[36] and MSAProbs[37] programs were used. The study used a direct sequence alignment of the hH3R sequence with template sequences and multiple alignment with sequences of other human histamine receptors (H1–H4) and human muscarinic (M1–M5). All sequences were obtained from UniProt catalog[78]. Generated structure alignments were manually verified to remove gaps in helices.

### 2. Homology modeling

Models were built using the rat muscarinic M3 (PDB code: 4U15) and human histamine H1 (PDB code: 3RZE) receptor crystal structures as templates. We used individual and joined templates based on both crystal structures at the same time to build H3R models. The programs MODELER 9.14[81] and Jackal—nest[82] as well as modeling services Swiss-Model[83] and I-TASSER[84] were applied for homology modeling. Modeling parameters are summarized in S1 Table in the supplementary data. A library of 1968 models was generated.

### 3. Energetic and qualitative assessment of models

The prepared library of homology models was subjected to multi-step assessment with the use of various tools. Step one involved an assessment of models with the BCL::Score[39] and QMEAN[40,85] functions. BCL::Score is a knowledge-based energy function using the Bayes’ theorem and the inverted Boltzmann relation developed from a larger number of membrane protein experimental structures. BCL::Score employs linearly weighted amino acid pairs and environmental potentials, a contact order potential, a beta-strand pairing potential and SSE formation and packing potentials. The compactness of a protein structure is assessed by the radius of gyration and the loop length potentials. QMEAN, *i*.*e*. Qualitative Model Energy Analysis, is a composite scoring function focused on geometrical aspects of protein structures. This function consists of five different descriptors: local geometry analyzed by a torsion angle potential; long-range interactions function using secondary structure-specific distance-dependent pairwise residue-level potentials; solvation potentials; agreement of secondary structure predicted by a protein secondary structure prediction *algorithm* called PSIPRED with the calculated secondary structure of the model using the dictionary of protein secondary structure (DSSP) database of secondary structure assignments; and solvent accessibility. The preselected models were further assessed with the multiple assessment tool PSVS[41]. PSVS combines several widely-used structure quality evaluation tools, such as PROCHECK, MolProbity, Verify3D and Prosa II. PSVS provides standard knowledge-based structure quality scores and global quality measures reported as Z-scores, based on high-resolution X-ray crystal structures. Based on the obtained report, the 21 top-ranked models were visually inspected and selected for further study.

### 4. Ligand preparation

**Fig 11:**
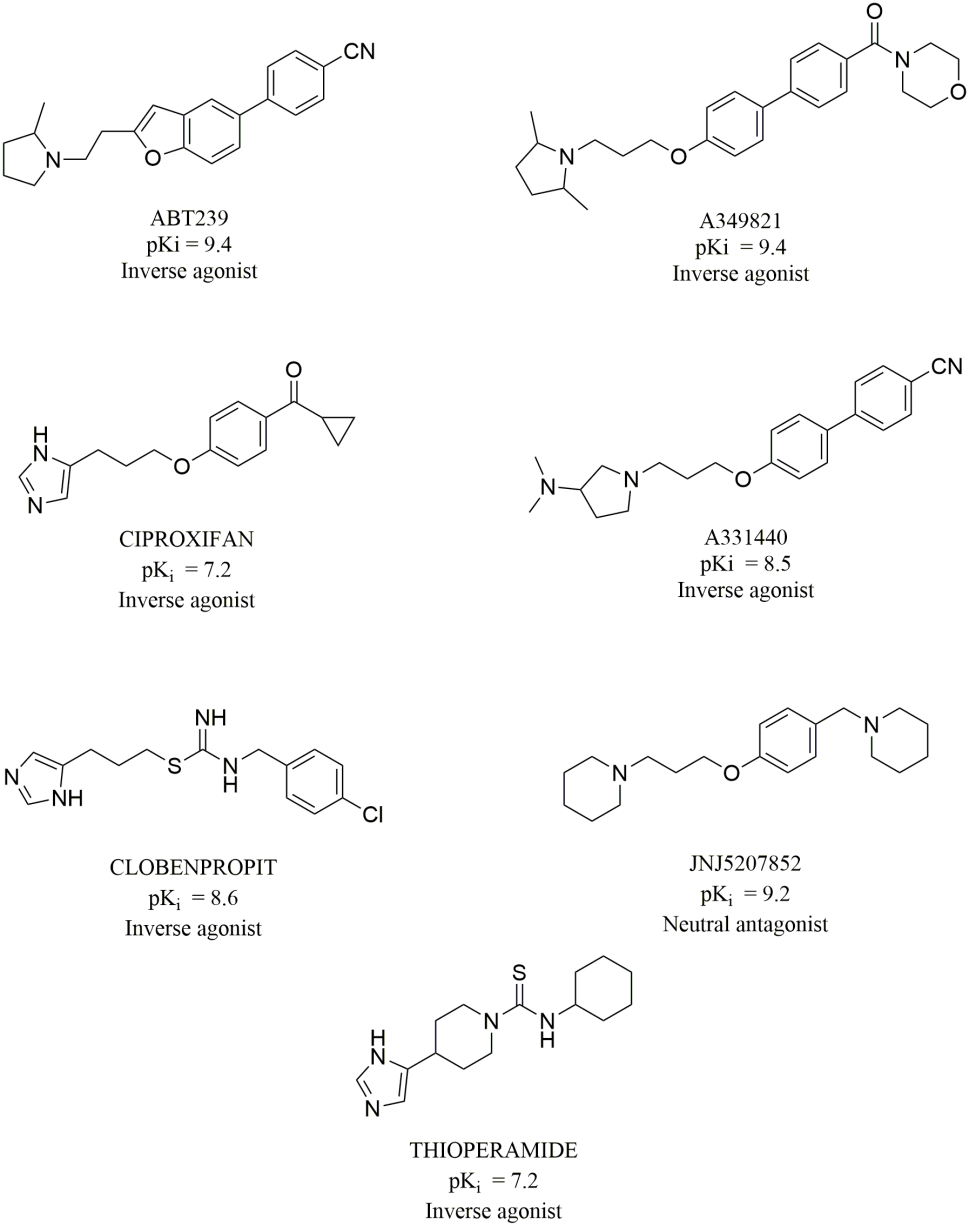
Structure of the histamine H3 receptor ligands used in the docking studies. pK_i_ values for ligand binding with the human H3 receptor [87].

In order to perform the docking studies, a set of compounds containing inverse agonists and antagonists was prepared. The 3D structures of 7 reference ligands (Fig 11) were created using Corina Online Demo[86]. Subsequently, Sybyl X 1.2 program was used to check the correctness of atom types and assign formal and partial charges.

### 5. Docking assessment of models

Docking studies were performed in two steps. Preliminary screening was conducted with GOLD 5.3 tool [72]. The tested receptor models were prepared in Hermes 1.7.0 tool. Protonation of all histidine residues were set to Nε, and all hydrogens were added. All atom and bond types were automatically set by Hermes 1.7.0. Reference compounds were docked into each receptor model using automatic settings of the genetic algorithm (GA) for very flexible docking and ranked with ChemPLP fitness scores. The binding site was defined as all residues within 22 Å of the carbon alpha (CA) atom of Asp 3.32. Ten poses per ligand were collected and analyzed. Five models with the best docked ligands were used for the second step of assessment with Glide 2016–3 from Schrӧdinger Suite[73]. As the best docked ligands were assumed to be ligands with the highest value of the scoring function and the most coherent binding mode with the lowest number of ligand poses out of the H3 receptor binding sites. Models were prepared with Protein Preparation Wizard. All hydrogens were added, and the order of bonds and disulfide bonds checked. H-bonds were assigned with PROPKA at pH 7.4. Docking was performed in extra precision (XP). The binding site was defined as a box of dimensions 22 × 22 × 22 Å, where the center was identified by the CA atom of Asp 3.32. The best model was chosen after both visual inspection of the obtained results and comparison of both docking studies.

### 6. Virtual screening performance test

In virtual screening we used models of antagonists and specific H3 decoys from GLL and GDD databases[70]. Ligands were randomly selected and separated to virtual screening library included 3000 compounds (300 Active and 2700 Decoys) with KNIME[71] List of used ligands is in the S2 Table in supplementary data. In the first stage of selection, we docked compounds with GOLD docking program and scored with ChemPLP Score [72]. Docking parameters and size of binding site were unchanged. In the second stage of screening ligands were docked with Glide using XP algorithm[73]. The final selection was based on GlideScore value. In rescoring studies the ligand poses received after glide docking were scored by two other scoring functions: ASP from GOLD program [72] and the available online DrugScoreX (DSX)[74].

## Conclusion

In this manuscript, we have presented homology modeling of the human histamine H3 receptor, including verification of model with hybrid assessment approaches. The molecular modeling experiments allowed us to create a homology model based on the template of the rat muscarinic M3 receptor bound with the antagonist, tiotropium in an inactive receptor conformation. This model proved to be the best among all those generated during the study. We compared the functions of several popular programs and services for homology modeling and many different matrices. The results confirm a significant impact of the templates used on the results of homology modeling and are in accordance with a correlation of high sequence homology with an increased accuracy of models. Among the programs and web sites used during the study, Modeller with the automodel algorithm turned out to be the best homology modeling program. Other algorithms, loopmodel and MyLoop did not improve the quality of the generated models. An excessive degree of refinement used in the program gave a negative effect in comparison with the medium refinement degree. Assessment of models that combined the protein evaluation functions BCL::Score and QMEAN, structural assessment tool PSVS and a two-step docking process supported by an analysis of the received results supported by literature data helped to generate a new model of higher accuracy. The simplicity of this procedure and relatively short computational time accelerated the process of protein structure determination, maintaining the accuracy of time-consuming molecular dynamics experiments. The two-step docking procedure allowed us to achieve efficient selection of models in terms of binding site reproduction. Detailed verification of the binding site is incredibly important when a homology model is prepared to study interactions with between receptor and ligands. Our model accurately reproduces conformations of all structural motifs found in aminergic GPCRs. The results of reference compound docking to the developed homology model correlated with the results of mutagenesis which assures us of their accuracy. After comparing them with other, previously proposed binding modes for the H3 receptor, we have come to the conclusion that the latest reports are relatively consistent with ours and receptor fragments indicated during our research have a significant effect on the binding of H3 receptor antagonists/inverse agonists. The modeling experiments, supported by literature data and visual inspection, were essential for accurate preparation of the model and prediction of the ligand binding mode. The knowledge acquired in this study, and the obtained model will be further used for design of novel ligands. We hope that its accuracy will be confirmed by the results of crystallographic studies. Preliminary results of virtual screening are not fully satisfactory. However, they enabled to select some ligand of confirmed activity. They also show how the evaluation function influence the effectiveness of virtual screening.

## Supporting information

S1 Fig Alignment of H3 receptor sequence from UniProt (UniProt Entry: Q9Y5N1) with rM3R template sequence (PDB: 4U15) used to prepare of the best model. (TIF)

S2 Fig BCL::Score and QMEAN score values for 21 models chosen for docking with GOLD. BCL::Score values are represented by green bars, QMEAN score are represented by red lozenge.
(TIF)

S3 Fig ChemPLP score for the best pose of each ligand after docking with GOLD. Colors represent the individual ligands: A331440—purple, A349821—orange, ABT 239—gray, Ciproxifan—yellow, Clobenpropit—red, JNJ520785—green, Thioperamide—blue.
(TIF)

S4 Fig Average RMSD value between all poses of each ligand in each docking. Colors represent the individual ligands: A331440—purple, A349821—orange, ABT 239—gray, Ciproxifan—yellow, Clobenpropit—red, JNJ520785—green, Thioperamide—blue.
(TIF)

S5 Fig Number of “successfully” docked ligand pose after docking with GOLD. Ligands placed within the orthosteric and/or allosteric site are called “successfully” docked. Colors represent the individual ligands: A331440—purple, A349821—orange, ABT 239—gray, Ciproxifan—yellow, Clobenpropit—red, JNJ520785—green, Thioperamide—blue.
(TIF)

S6 Fig Absolute values of GlideScore for each docking. Colors represent the individual ligands: A331440—purple, A349821—orange, ABT 239—gray, Ciproxifan—yellow, Clobenpropit—red, JNJ520785—green, Thioperamide—blue.
(TIF)

S1 Table Detailed parameters of homology modeling process with the programs Modeller, Jackal and the web-services I-Tasser, Swis-Model. # Template and modeling parameters used for the best model. * H—UniProt sequences of human histamine receptors H1-H4, M—UniProt sequences of human muscarinic receptors M1-M5, H3 –UniProt sequence of hH3R, 3RZE—sequence of hH1 histamine receptor model from PDB (PDB: 3RZE), 4U15—sequence of rM3 muscarinic receptor model from PDB (PDB: 4U15).
(DOCX)

S2 Table Detailed list of ligands from GLL and GDD datasets with activity tags. (DOCX)

## Abbreviations

CA: carbon alpha atom
CASP: Critical Assessment of protein Structure Prediction
CNS: central nervous system
GA: genetic algorithm
GPCRs: G-protein coupled receptors
H3R: histamine H3 receptor
hH1R: human histamine H1 receptor
hH3R: human histamine H3 receptor
ICL: intracellular loop
PDB: Protein data bank
RMSD: Root-mean-square deviation
rM3R: rat M3 muscarinic receptor
TMH: Transmembrane helices
XP: extra precision
