# Supplementary figures and images for: Hybrid approach to structure modeling of the histamine H3 receptor: Multi-level assessment as a tool for model verification
Source: PLoS One. 2017 Oct 5;12(10):e0186108. doi: 10.1371/journal.pone.0186108 (PMC5629032; doi:10.1371/journal.pone.0186108)

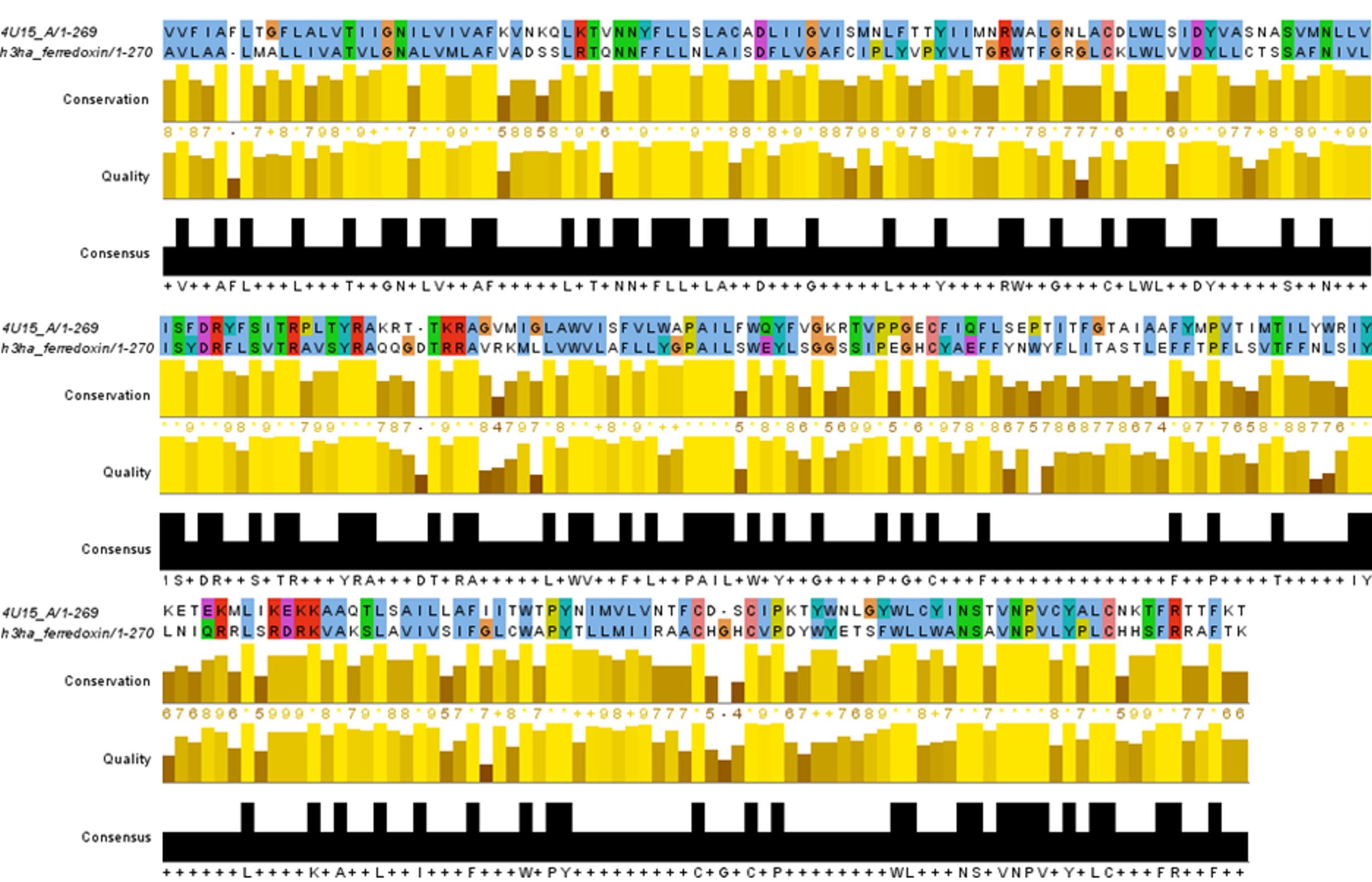

Supplement: S1 Fig — (TIF) [file pone.0186108.s001.tif]

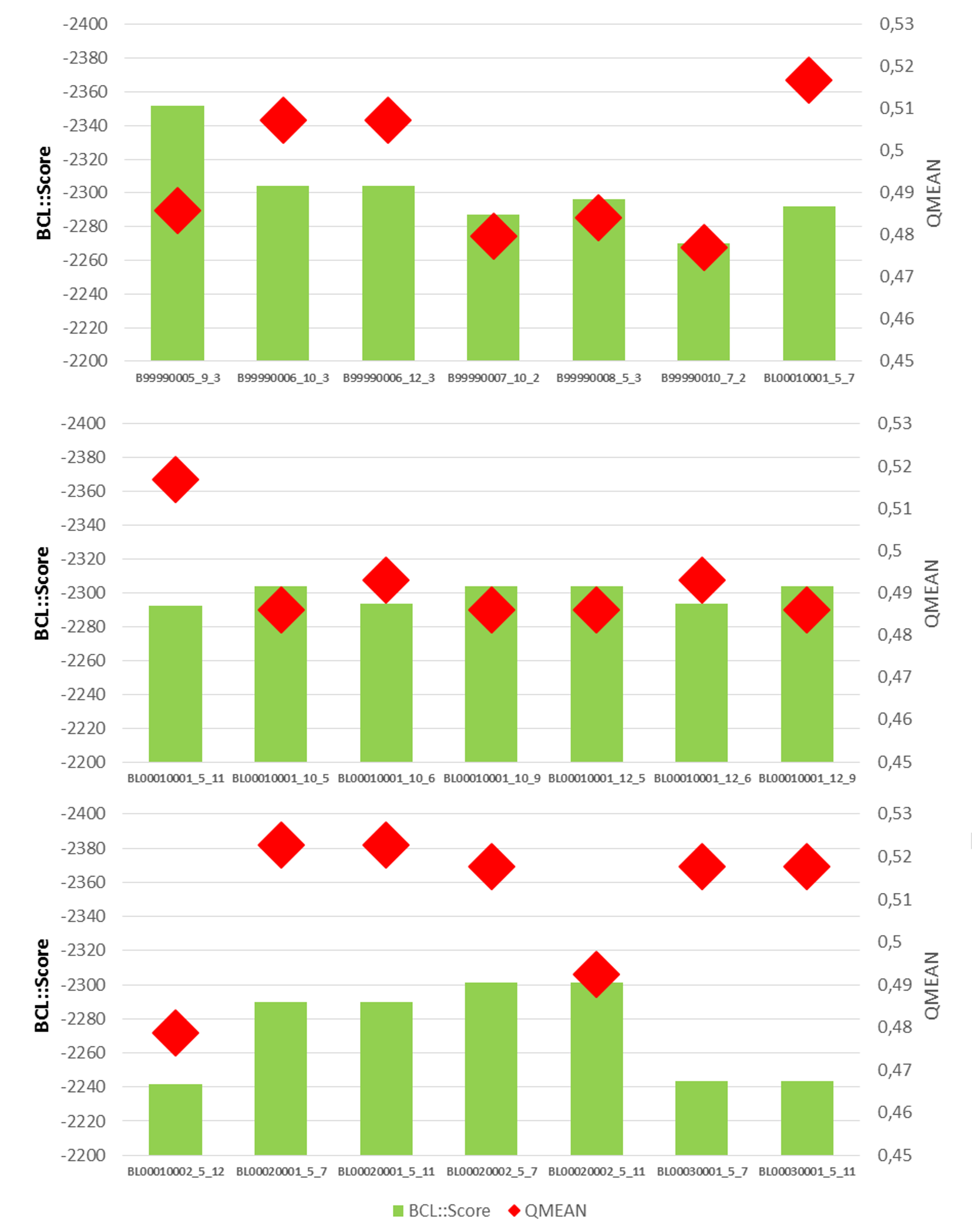

Supplement: S2 Fig — BCL::Score values are represented by green bars, QMEAN score are represented by red lozenge. (TIF) [file pone.0186108.s002.tif]

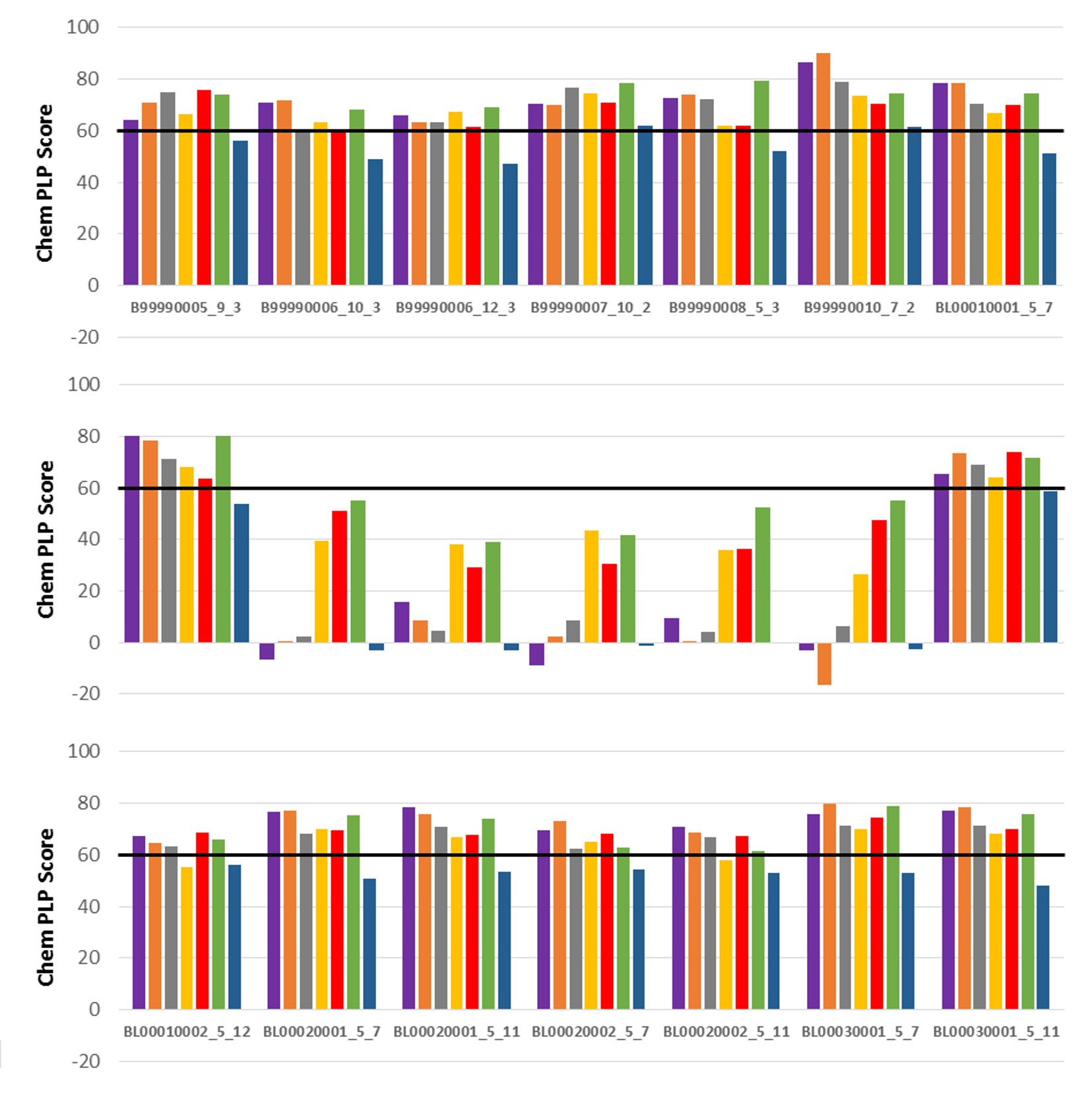

Supplement: S3 Fig — Colors represent the individual ligands: A331440—purple, A349821—orange, ABT 239—gray, Ciproxifan—yellow, Clobenpropit—red, JNJ520785—green, Thioperamide—blue. (TIF) [file pone.0186108.s003.tif]

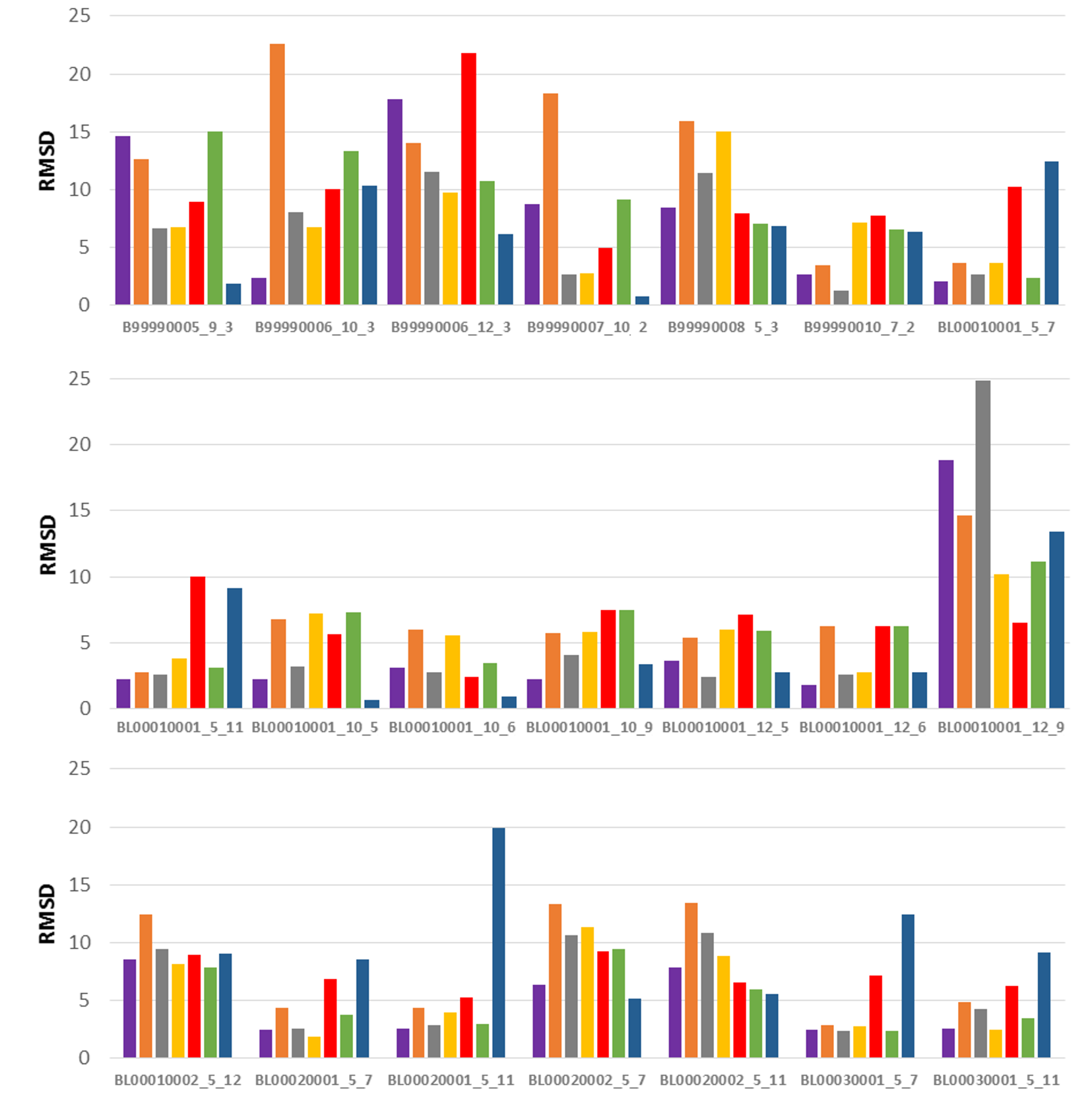

Supplement: S4 Fig — Colors represent the individual ligands: A331440—purple, A349821—orange, ABT 239—gray, Ciproxifan—yellow, Clobenpropit—red, JNJ520785—green, Thioperamide—blue. (TIF) [file pone.0186108.s004.tif]

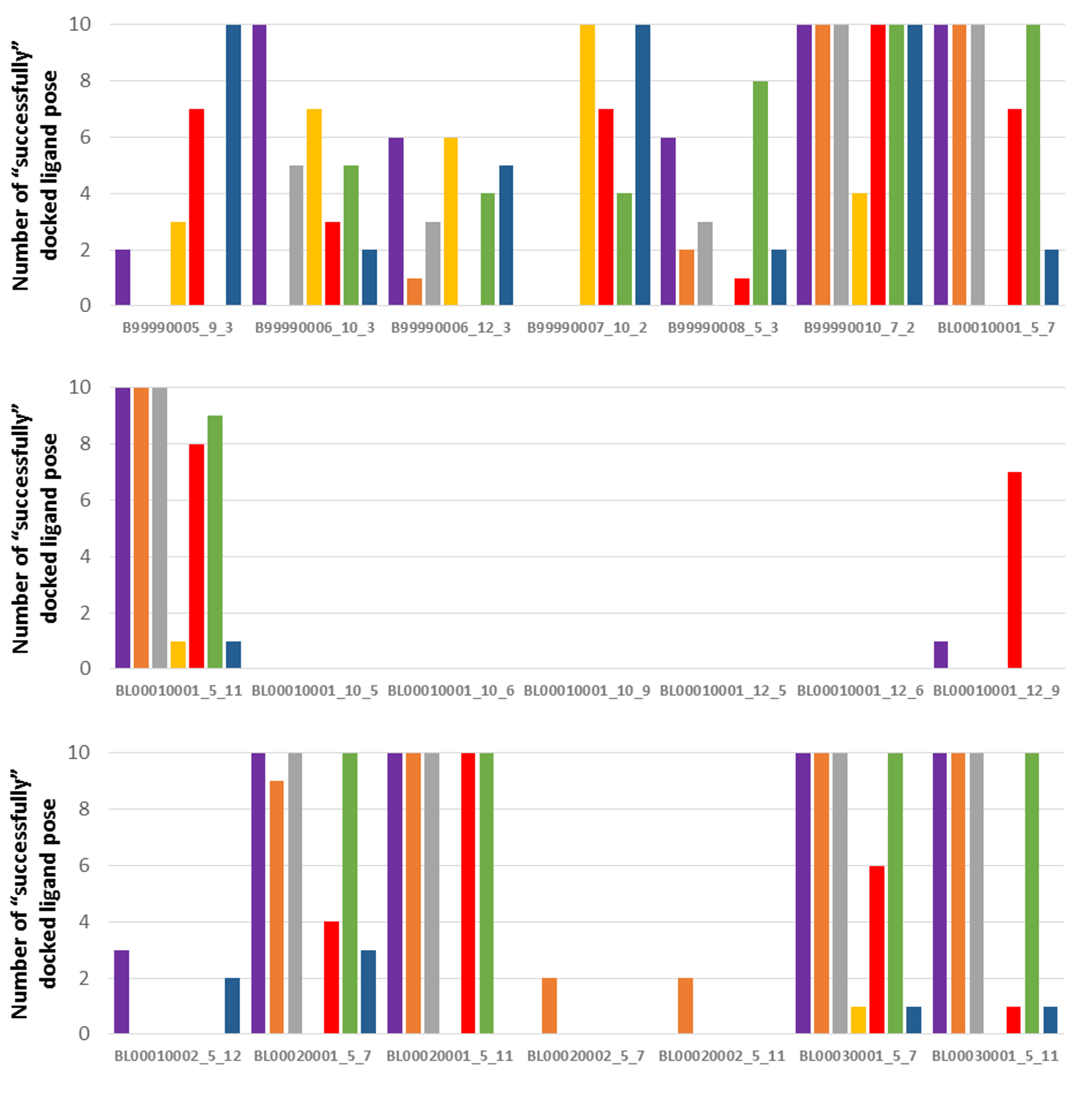

Supplement: S5 Fig — Ligands placed within the orthosteric and/or allosteric site are called “successfully” docked. Colors represent the individual ligands: A331440—purple, A349821—orange, ABT 239—gray, Ciproxifan—yellow, Clobenpropit—red, JNJ520785—green, Thioperamide—blue. (TIF) [file pone.0186108.s005.tif]

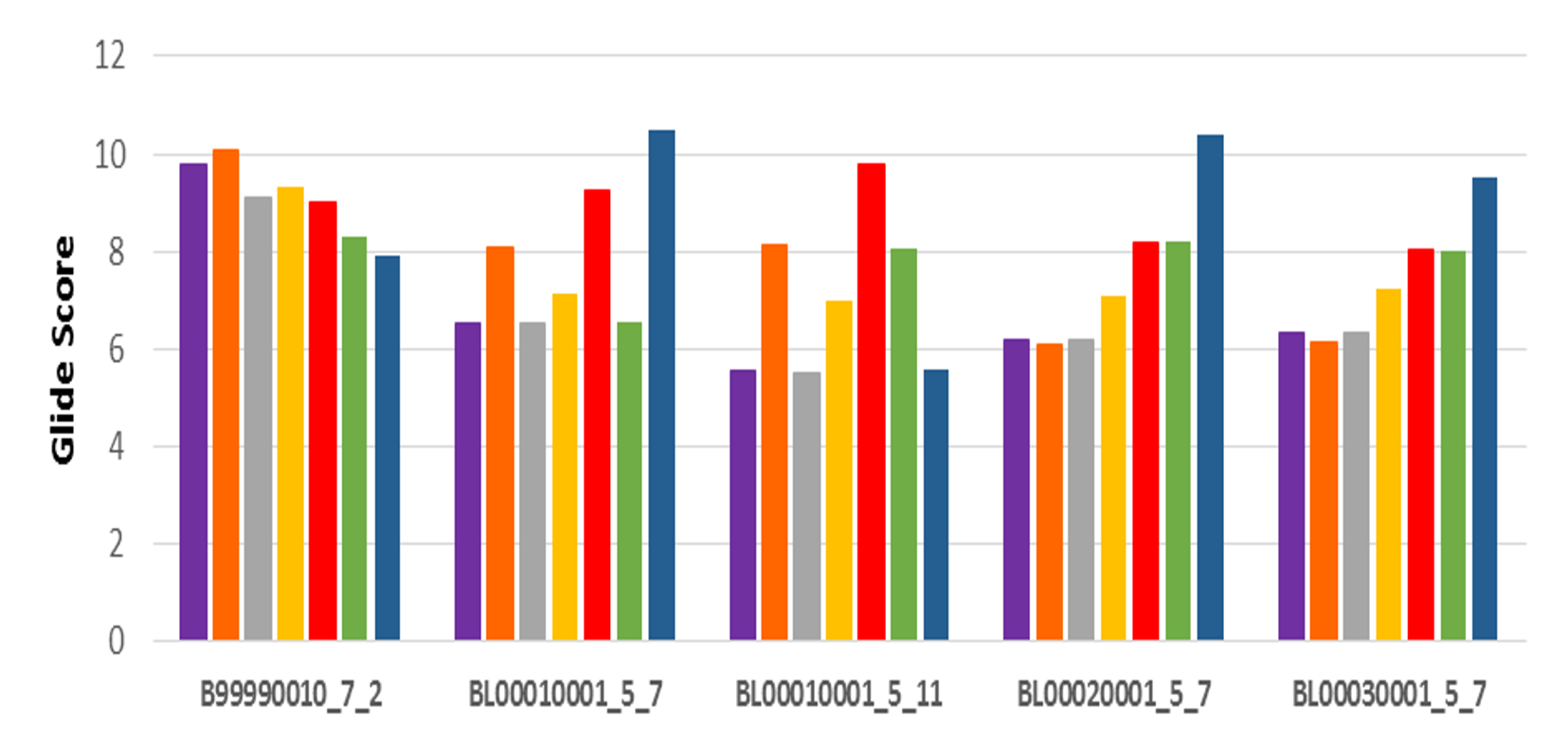

Supplement: S6 Fig — Colors represent the individual ligands: A331440—purple, A349821—orange, ABT 239—gray, Ciproxifan—yellow, Clobenpropit—red, JNJ520785—green, Thioperamide—blue. (TIF) [file pone.0186108.s006.tif]
