# Supplementary material for: Hybrid approach to structure modeling of the histamine H3 receptor: Multi-level assessment as a tool for model verification
Source: PLoS One. 2017 Oct 5;12(10):e0186108. doi: 10.1371/journal.pone.0186108 (PMC5629032; doi:10.1371/journal.pone.0186108)
Supplement: S1 Table — # Template and modeling parameters used for the best model. * H—UniProt sequences of human histamine receptors H1-H4, M—UniProt sequences of human muscarinic receptors M1-M5, H3 –UniProt sequence of hH3R, 3RZE—sequence of hH1 histamine receptor model from PDB (PDB: 3RZE), 4U15—sequence of rM3 muscarinic receptor model from PDB (PDB: 4U15). (DOCX) [file pone.0186108.s007.docx]

| Program/Web service | Alignment No | Alignment tool | Used receptor sequences* | Homology modeling parameters No | | Homology modeling parameters detail |
| --- | --- | --- | --- | --- | --- | --- |
| Modeller | 1 | Clustal Omega | H/H3/3RZE | 1 | | automodel / 9 models / no refinement |
|  | 2 | Clustal Omega | H3/3RZE | 2# | | automodel / 9 models / very fast refinement |
|  | 3 | MSAProbe | H/H3/3RZE | 3 | | automodel / 9 models / slow refinement |
|  | 4 | MSAProbe | H3/3RZE | 4 | | automodel / 9 models / slow large refinement |
|  | 5 | Clustal Omega | H3/4U15 | 5 | | loopmodel / 9 models / no refinement |
|  | 6 | MSAProbe | H3/4U15 | 6 | | loopmodel / 9 models / very fast refinement |
|  | 7# | Clustal Omega | M/H3/4U15 | 7 | | loopmodel / 9 models / slow refinement |
|  | 8 | MSAProbe | M/H3/4U15 | 8 | | loopmodel / 9 models / slow large refinement |
|  | 9 | Clustal Omega | H3/4U15/3RZE | 9 | | MyLoop / 9 models / no refinement |
|  | 10 | MSAProbe | H3/4U15/3RZE | 10 | | MyLoop / 9 models / very fast refinement |
|  | 11 | Clustal Omega | H/M/H3/4U15/3RZE | 11 | | MyLoop / 9 models / slow refinement |
|  | 12 | MSAProbe | H/M/H3/4U15/3RZE | 12 | | MyLoop / 9 models / slow large refinement |
| Jackal - nest | 1 | Clustal Omega | H/H3/3RZE | 1 | | -fast 3 -tune 0  -opt 1 -nopt 1 |
|  |  |  |  | 2 | | -fast 3 -tune 0  -opt 3 -nopt 1 |
|  |  |  |  | 3 | | -fast 3 -tune 0  -opt 4 -nopt 1 |
|  | 2 | Clustal Omega | H3/3RZE | 4 | | -fast 3 -tune 0  -opt 1 -nopt 1 |
|  |  |  |  | 5 | | -fast 3 -tune 0  -opt 3 -nopt 1 |
|  |  |  |  | 6 | | -fast 3 -tune 0  -opt 4 -nopt 1 |
|  | 3 | MSAProbe | H/H3/3RZE | 7 | | -fast 3 -tune 0  -opt 1 -nopt 1 |
|  |  |  |  | 8 | | -fast 3 -tune 0  -opt 3 -nopt 1 |
|  |  |  |  | 9 | | -fast 3 -tune 0  -opt 4 -nopt 1 |
|  | 4 | MSAProbe | H3/3RZE | 10 | | -fast 3 -tune 0  -opt 1 -nopt 1 |
|  |  |  |  | 11 | | -fast 3 -tune 0  -opt 3 -nopt 1 |
|  |  |  |  | 12 | | -fast 3 -tune 0  -opt 4 -nopt 1 |
|  | 5 | Clustal Omega | M/H3/4U15 | 13 | | -fast 3 -tune 0  -opt 1 -nopt 1 |
|  |  |  |  | 14 | | -fast 3 -tune 0  -opt 3 -nopt 1 |
|  |  |  |  | 15 | | -fast 3 -tune 0  -opt 4 -nopt 1 |
|  | 6 | MSAProbe | H3/4U15 | 16 | | -fast 3 -tune 0  -opt 1 -nopt 1 |
|  |  |  |  | 17 | | -fast 3 -tune 0  -opt 3 -nopt 1 |
|  |  |  |  | 18 | | -fast 3 -tune 0  -opt 4 -nopt 1 |
|  | 7 | Clustal Omega | H3/4U15 | 19 | | -fast 3 -tune 0  -opt 1 -nopt 1 |
|  |  |  |  | 20 | | -fast 3 -tune 0  -opt 3 -nopt 1 |
|  |  |  |  | 21 | | -fast 3 -tune 0  -opt 4 -nopt 1 |
|  | 8 | MSAProbe | M/H3/4U15 | 22 | | -fast 3 -tune 0  -opt 1 -nopt 1 |
|  |  |  |  | 23 | | -fast 3 -tune 0  -opt 3 -nopt 1 |
|  |  |  |  | 24 | | -fast 3 -tune 0  -opt 4 -nopt 1 |
|  | 9 | Clustal Omega | H3/4U15/3RZE | 25 | | -fast 3 -tune 0  -opt 1 -nopt 1 |
|  |  |  |  | 26 | -fast 3 -tune 0  -opt 3 -nopt 1 | |
|  |  |  |  | 27 | -fast 3 -tune 0  -opt 4 -nopt 1 | |
|  | 10 | MSAProbe | H3/4U15/3RZE | 28 | -fast 3 -tune 0  -opt 1 -nopt 1 | |
|  |  |  |  | 29 | -fast 3 -tune 0  -opt 3 -nopt 1 | |
|  |  |  |  | 30 | -fast 3 -tune 0  -opt 4 -nopt 1 | |
|  | 11 | Clustal Omega | H/M/H3/4U15/3RZE | 31 | -fast 3 -tune 0  -opt 1 -nopt 1 | |
|  |  |  |  | 32 | -fast 3 -tune 0  -opt 3 -nopt 1 | |
|  |  |  |  | 33 | -fast 3 -tune 0  -opt 4 -nopt 1 | |
|  | 12 | MSAProbe | H/M/H3/4U15/3RZE | 34 | -fast 3 -tune 0  -opt 1 -nopt 1 | |
|  |  |  |  | 35 | -fast 3 -tune 0  -opt 3 -nopt 1 | |
|  |  |  |  | 36 | -fast 3 -tune 0  -opt 4 -nopt 1 | |
| SwissModel | 1 | Clustal Omega | H/H3/3RZE | Standard options | | |
|  | 2 | Clustal Omega | H3/3RZE |  |  |  |
|  | 3 | MSAProbe | H/H3/3RZE |  |  |  |
|  | 4 | MSAProbe | H3/3RZE |  |  |  |
|  | 5 | Clustal Omega | H3/4U15 |  |  |  |
|  | 6 | MSAProbe | H3/4U15 |  |  |  |
|  | 7 | Clustal Omega | M/H3/4U15 |  |  |  |
|  | 8 | MSAProbe | M/H3/4U15 |  |  |  |
|  | 9 | Clustal Omega | H3/4U15/3RZE |  |  |  |
|  | 10 | MSAProbe | H3/4U15/3RZE |  |  |  |
|  | 11 | Clustal Omega | H/M/H3/4U15/3RZE |  |  |  |
|  | 12 | MSAProbe | H/M/H3/4U15/3RZE |  |  |  |
| I-TASSER | 1 | Clustal Omega | H/H3/3RZE | Standard options | | |
|  | 2 | Clustal Omega | H3/3RZE |  |  |  |
|  | 3 | MSAProbe | H/H3/3RZE |  |  |  |
|  | 4 | MSAProbe | H3/3RZE |  |  |  |
|  | 5 | Clustal Omega | H3/4U15 |  |  |  |
|  | 6 | MSAProbe | H3/4U15 |  |  |  |
|  | 7 | Clustal Omega | M/H3/4U15 |  |  |  |
|  | 8 | MSAProbe | M/H3/4U15 |  |  |  |
|  | 9 | Clustal Omega | H3/4U15/3RZE |  |  |  |
|  | 10 | MSAProbe | H3/4U15/3RZE |  |  |  |
|  | 11 | Clustal Omega | H/M/H3/4U15/3RZE |  |  |  |
|  | 12 | MSAProbe | H/M/H3/4U15/3RZE |  |  |  |

**Table 1S. Detailed parameters of homology modeling process with the programs Modeller, Jackal and the web-services I-Tasser, Swis-Model.**

# Template and modeling parameters used for the best model

* H – UniProt sequences of human histamine receptors H1-H4, M – UniProt sequences of human muscarinic receptors M1-M5, H3 – UniProt sequence of hH3R, 3RZE – sequence of hH1 histamine receptor model from PDB (PDB: 3RZE), 4U15 - sequence of rM3 muscarinic receptor model from PDB (PDB: 4U15)
