# Supplementary material for: Hybrid approach to structure modeling of the histamine H3 receptor: Multi-level assessment as a tool for model verification
Source: PLoS One. 2017 Oct 5;12(10):e0186108. doi: 10.1371/journal.pone.0186108 (PMC5629032; doi:10.1371/journal.pone.0186108)
Supplement: S2 Table — (DOCX) [file pone.0186108.s008.docx]

| GLL/GDD Title | Activity Tag | GLL/GDD Title | Activity Tag | GLL/GDD Title | Activity Tag |
| --- | --- | --- | --- | --- | --- |
| 2790 | Active | ZINC11784668 | Decoy | ZINC40742027 | Decoy |
| 132937 | Active | ZINC11788785 | Decoy | ZINC40742418 | Decoy |
| 153971 | Active | ZINC11789080 | Decoy | ZINC40742797 | Decoy |
| 156615 | Active | ZINC11803973 | Decoy | ZINC40744258 | Decoy |
| 2766326 | Active | ZINC11819123 | Decoy | ZINC40835973 | Decoy |
| 2994072 | Active | ZINC11830464 | Decoy | ZINC40845073 | Decoy |
| 2994314 | Active | ZINC11830944 | Decoy | ZINC40847518 | Decoy |
| 2994465 | Active | ZINC11839351 | Decoy | ZINC40863054 | Decoy |
| 3035746 | Active | ZINC11843862 | Decoy | ZINC40864762 | Decoy |
| 3035905 | Active | ZINC11873093 | Decoy | ZINC40889125 | Decoy |
| 5250783 | Active | ZINC11874614 | Decoy | ZINC40892189 | Decoy |
| 5311293 | Active | ZINC11881315 | Decoy | ZINC40915124 | Decoy |
| 6421295 | Active | ZINC11892738 | Decoy | ZINC40915128 | Decoy |
| 9578063 | Active | ZINC11906259 | Decoy | ZINC40916250 | Decoy |
| 9793791 | Active | ZINC11911260 | Decoy | ZINC40918395 | Decoy |
| 9793868 | Active | ZINC11912532 | Decoy | ZINC40938891 | Decoy |
| 9795345 | Active | ZINC11936356 | Decoy | ZINC40943327 | Decoy |
| 9795832 | Active | ZINC11965449 | Decoy | ZINC40950250 | Decoy |
| 9798324 | Active | ZINC11998310 | Decoy | ZINC40972701 | Decoy |
| 9798429 | Active | ZINC12001424 | Decoy | ZINC40973108 | Decoy |
| 9798478 | Active | ZINC12020363 | Decoy | ZINC40976314 | Decoy |
| 9799053 | Active | ZINC12026642 | Decoy | ZINC40985688 | Decoy |
| 9800365 | Active | ZINC12050577 | Decoy | ZINC41113047 | Decoy |
| 9800462 | Active | ZINC12052765 | Decoy | ZINC41113097 | Decoy |
| 9801137 | Active | ZINC12072840 | Decoy | ZINC41130800 | Decoy |
| 9801743 | Active | ZINC12073890 | Decoy | ZINC41158359 | Decoy |
| 9808451 | Active | ZINC12090163 | Decoy | ZINC41185518 | Decoy |
| 9812816 | Active | ZINC12116763 | Decoy | ZINC41215496 | Decoy |
| 9816459 | Active | ZINC12118130 | Decoy | ZINC41222665 | Decoy |
| 9818278 | Active | ZINC12126303 | Decoy | ZINC41258564 | Decoy |
| 9818558 | Active | ZINC12186809 | Decoy | ZINC41277957 | Decoy |
| 9818947 | Active | ZINC12188710 | Decoy | ZINC41278294 | Decoy |
| 9819578 | Active | ZINC12192925 | Decoy | ZINC41293042 | Decoy |
| 9820310 | Active | ZINC12202974 | Decoy | ZINC41318018 | Decoy |
| 9821266 | Active | ZINC12300668 | Decoy | ZINC41318034 | Decoy |
| 9821801 | Active | ZINC12302384 | Decoy | ZINC41319193 | Decoy |
| 9834003 | Active | ZINC12315003 | Decoy | ZINC41332194 | Decoy |
| 9838650 | Active | ZINC12328724 | Decoy | ZINC41334403 | Decoy |
| 9838905 | Active | ZINC12363672 | Decoy | ZINC41335030 | Decoy |
| 9839279 | Active | ZINC12372363 | Decoy | ZINC41336702 | Decoy |
| 9839416 | Active | ZINC12385673 | Decoy | ZINC41336926 | Decoy |
| 9839975 | Active | ZINC12387459 | Decoy | ZINC41341282 | Decoy |
| 9839979 | Active | ZINC12410523 | Decoy | ZINC41344951 | Decoy |
| 9840564 | Active | ZINC12418815 | Decoy | ZINC41369128 | Decoy |
| 9840770 | Active | ZINC12431642 | Decoy | ZINC41372365 | Decoy |
| 9840856 | Active | ZINC12438981 | Decoy | ZINC41372394 | Decoy |
| 9841261 | Active | ZINC12442399 | Decoy | ZINC41376495 | Decoy |
| 9842018 | Active | ZINC12452060 | Decoy | ZINC41391855 | Decoy |
| 9842083 | Active | ZINC12457028 | Decoy | ZINC41391865 | Decoy |
| 9855483 | Active | ZINC12467675 | Decoy | ZINC41392005 | Decoy |
| 9855565 | Active | ZINC12470684 | Decoy | ZINC41392240 | Decoy |
| 9857606 | Active | ZINC12474703 | Decoy | ZINC41392550 | Decoy |
| 9858195 | Active | ZINC12475237 | Decoy | ZINC41417760 | Decoy |
| 9859014 | Active | ZINC12478948 | Decoy | ZINC41420043 | Decoy |
| 9860144 | Active | ZINC12492779 | Decoy | ZINC41426337 | Decoy |
| 9862335 | Active | ZINC12494779 | Decoy | ZINC41449972 | Decoy |
| 9862702 | Active | ZINC12496950 | Decoy | ZINC41452708 | Decoy |
| 9862741 | Active | ZINC12506219 | Decoy | ZINC41465577 | Decoy |
| 9862753 | Active | ZINC12528108 | Decoy | ZINC41468310 | Decoy |
| 9863682 | Active | ZINC12531906 | Decoy | ZINC41473567 | Decoy |
| 9864398 | Active | ZINC12577307 | Decoy | ZINC41474319 | Decoy |
| 9865181 | Active | ZINC12580664 | Decoy | ZINC41474823 | Decoy |
| 9877600 | Active | ZINC12591142 | Decoy | ZINC41491720 | Decoy |
| 9877711 | Active | ZINC12591741 | Decoy | ZINC41495929 | Decoy |
| 9880039 | Active | ZINC12594425 | Decoy | ZINC41497860 | Decoy |
| 9881433 | Active | ZINC12596505 | Decoy | ZINC41507409 | Decoy |
| 9882961 | Active | ZINC12600854 | Decoy | ZINC41510108 | Decoy |
| 9883538 | Active | ZINC12607340 | Decoy | ZINC41531075 | Decoy |
| 9883980 | Active | ZINC12608769 | Decoy | ZINC41535427 | Decoy |
| 9884432 | Active | ZINC12614998 | Decoy | ZINC41538189 | Decoy |
| 9884503 | Active | ZINC12620893 | Decoy | ZINC41569379 | Decoy |
| 9884637 | Active | ZINC12653870 | Decoy | ZINC41571190 | Decoy |
| 9885196 | Active | ZINC12718858 | Decoy | ZINC41590613 | Decoy |
| 9885602 | Active | ZINC12730837 | Decoy | ZINC41596268 | Decoy |
| 9886975 | Active | ZINC12747966 | Decoy | ZINC41601737 | Decoy |
| 9889593 | Active | ZINC12749944 | Decoy | ZINC41603520 | Decoy |
| 9898875 | Active | ZINC12766172 | Decoy | ZINC41603606 | Decoy |
| 9902158 | Active | ZINC12768420 | Decoy | ZINC41603609 | Decoy |
| 9902971 | Active | ZINC12781165 | Decoy | ZINC41605144 | Decoy |
| 9904299 | Active | ZINC12812621 | Decoy | ZINC41605167 | Decoy |
| 9905489 | Active | ZINC12835068 | Decoy | ZINC41613282 | Decoy |
| 9906249 | Active | ZINC12853909 | Decoy | ZINC41626192 | Decoy |
| 9907274 | Active | ZINC12864658 | Decoy | ZINC41627547 | Decoy |
| 9907619 | Active | ZINC12873883 | Decoy | ZINC41701279 | Decoy |
| 9907832 | Active | ZINC12882826 | Decoy | ZINC41732233 | Decoy |
| 9909735 | Active | ZINC12931257 | Decoy | ZINC41733159 | Decoy |
| 9913148 | Active | ZINC12943054 | Decoy | ZINC41773855 | Decoy |
| 9923194 | Active | ZINC12985023 | Decoy | ZINC41778357 | Decoy |
| 9923762 | Active | ZINC12989234 | Decoy | ZINC41790874 | Decoy |
| 9925192 | Active | ZINC12998889 | Decoy | ZINC41817242 | Decoy |
| 9925627 | Active | ZINC13014843 | Decoy | ZINC41828409 | Decoy |
| 9927501 | Active | ZINC13040738 | Decoy | ZINC41835444 | Decoy |
| 9928168 | Active | ZINC13208980 | Decoy | ZINC41835460 | Decoy |
| 9928224 | Active | ZINC13233886 | Decoy | ZINC41840655 | Decoy |
| 9929500 | Active | ZINC13281751 | Decoy | ZINC41868917 | Decoy |
| 9929812 | Active | ZINC13303611 | Decoy | ZINC41873150 | Decoy |
| 9930868 | Active | ZINC13371457 | Decoy | ZINC41893345 | Decoy |
| 9942159 | Active | ZINC13470999 | Decoy | ZINC41895232 | Decoy |
| 9942615 | Active | ZINC13471168 | Decoy | ZINC41898260 | Decoy |
| 9944537 | Active | ZINC13472435 | Decoy | ZINC41928362 | Decoy |
| 9945660 | Active | ZINC13477604 | Decoy | ZINC41928643 | Decoy |
| 9947227 | Active | ZINC13487644 | Decoy | ZINC41936383 | Decoy |
| 9948238 | Active | ZINC13488712 | Decoy | ZINC41958215 | Decoy |
| 9948809 | Active | ZINC13491001 | Decoy | ZINC41968674 | Decoy |
| 9949017 | Active | ZINC13491394 | Decoy | ZINC42007848 | Decoy |
| 9949508 | Active | ZINC13491395 | Decoy | ZINC42012249 | Decoy |
| 9951297 | Active | ZINC13492357 | Decoy | ZINC42012293 | Decoy |
| 9953505 | Active | ZINC13492361 | Decoy | ZINC42017569 | Decoy |
| 9954712 | Active | ZINC13513506 | Decoy | ZINC42025374 | Decoy |
| 9964410 | Active | ZINC13519647 | Decoy | ZINC42032969 | Decoy |
| 9966176 | Active | ZINC13527078 | Decoy | ZINC42065010 | Decoy |
| 9966707 | Active | ZINC13538119 | Decoy | ZINC42096345 | Decoy |
| 9967231 | Active | ZINC13554544 | Decoy | ZINC42096709 | Decoy |
| 9968975 | Active | ZINC13558992 | Decoy | ZINC42096879 | Decoy |
| 9969015 | Active | ZINC13569017 | Decoy | ZINC42097078 | Decoy |
| 9997296 | Active | ZINC13570481 | Decoy | ZINC42099629 | Decoy |
| 10030750 | Active | ZINC13574031 | Decoy | ZINC42102527 | Decoy |
| 10036954 | Active | ZINC13629880 | Decoy | ZINC42103464 | Decoy |
| 10044892 | Active | ZINC13651667 | Decoy | ZINC42104173 | Decoy |
| 10060425 | Active | ZINC13679126 | Decoy | ZINC42104508 | Decoy |
| 10068871 | Active | ZINC13684823 | Decoy | ZINC42105685 | Decoy |
| 10087654 | Active | ZINC13685552 | Decoy | ZINC42106579 | Decoy |
| 10109296 | Active | ZINC13685556 | Decoy | ZINC42109954 | Decoy |
| 10114059 | Active | ZINC13685603 | Decoy | ZINC42117534 | Decoy |
| 10130038 | Active | ZINC13717450 | Decoy | ZINC42117703 | Decoy |
| 10131681 | Active | ZINC13717898 | Decoy | ZINC42122332 | Decoy |
| 10132505 | Active | ZINC13718198 | Decoy | ZINC42127311 | Decoy |
| 10132506 | Active | ZINC13724090 | Decoy | ZINC42140365 | Decoy |
| 10132950 | Active | ZINC13724316 | Decoy | ZINC42148343 | Decoy |
| 10151849 | Active | ZINC13724780 | Decoy | ZINC42148468 | Decoy |
| 10154479 | Active | ZINC13724892 | Decoy | ZINC42186562 | Decoy |
| 10154481 | Active | ZINC13725749 | Decoy | ZINC42197226 | Decoy |
| 10176358 | Active | ZINC13725799 | Decoy | ZINC42200543 | Decoy |
| 10177152 | Active | ZINC13727618 | Decoy | ZINC42207707 | Decoy |
| 10177985 | Active | ZINC13729972 | Decoy | ZINC42207949 | Decoy |
| 10198541 | Active | ZINC13730442 | Decoy | ZINC42208025 | Decoy |
| 10218078 | Active | ZINC13733209 | Decoy | ZINC42211634 | Decoy |
| 10219593 | Active | ZINC13734503 | Decoy | ZINC42212147 | Decoy |
| 10219597 | Active | ZINC13749802 | Decoy | ZINC42218685 | Decoy |
| 10220233 | Active | ZINC13755772 | Decoy | ZINC42229154 | Decoy |
| 10220282 | Active | ZINC13758342 | Decoy | ZINC42236311 | Decoy |
| 10220389 | Active | ZINC13758344 | Decoy | ZINC42236830 | Decoy |
| 10220586 | Active | ZINC13773995 | Decoy | ZINC42289079 | Decoy |
| 10222531 | Active | ZINC13778323 | Decoy | ZINC42309209 | Decoy |
| 10225493 | Active | ZINC13781171 | Decoy | ZINC42320425 | Decoy |
| 10269256 | Active | ZINC13805564 | Decoy | ZINC42326663 | Decoy |
| 10270093 | Active | ZINC13819517 | Decoy | ZINC42328275 | Decoy |
| 10273802 | Active | ZINC13825216 | Decoy | ZINC42349437 | Decoy |
| 10291489 | Active | ZINC13834518 | Decoy | ZINC42353423 | Decoy |
| 10376572 | Active | ZINC13838050 | Decoy | ZINC42354789 | Decoy |
| 10381995 | Active | ZINC13860171 | Decoy | ZINC42366117 | Decoy |
| 10405078 | Active | ZINC13862283 | Decoy | ZINC42383879 | Decoy |
| 10420612 | Active | ZINC13864157 | Decoy | ZINC42389068 | Decoy |
| 10421930 | Active | ZINC13864185 | Decoy | ZINC42391964 | Decoy |
| 10451094 | Active | ZINC13904275 | Decoy | ZINC42409794 | Decoy |
| 10536116 | Active | ZINC13940834 | Decoy | ZINC42470859 | Decoy |
| 10560537 | Active | ZINC13945873 | Decoy | ZINC42482593 | Decoy |
| 10635213 | Active | ZINC13989229 | Decoy | ZINC42501705 | Decoy |
| 10659856 | Active | ZINC13989290 | Decoy | ZINC42509744 | Decoy |
| 10682710 | Active | ZINC13989420 | Decoy | ZINC42510671 | Decoy |
| 10684712 | Active | ZINC13990753 | Decoy | ZINC42556998 | Decoy |
| 10731224 | Active | ZINC13992843 | Decoy | ZINC42560158 | Decoy |
| 10758236 | Active | ZINC13994627 | Decoy | ZINC42560359 | Decoy |
| 11152613 | Active | ZINC13997466 | Decoy | ZINC42588640 | Decoy |
| 11187510 | Active | ZINC14001545 | Decoy | ZINC42625879 | Decoy |
| 11278861 | Active | ZINC14005609 | Decoy | ZINC42632806 | Decoy |
| 11279681 | Active | ZINC14015361 | Decoy | ZINC42632830 | Decoy |
| 11291836 | Active | ZINC14019244 | Decoy | ZINC42637780 | Decoy |
| 11304221 | Active | ZINC14094487 | Decoy | ZINC42649460 | Decoy |
| 11324846 | Active | ZINC14096345 | Decoy | ZINC42652487 | Decoy |
| 11345716 | Active | ZINC14098395 | Decoy | ZINC42656026 | Decoy |
| 11372022 | Active | ZINC14161961 | Decoy | ZINC42669684 | Decoy |
| 11383530 | Active | ZINC14183911 | Decoy | ZINC42675198 | Decoy |
| 11405078 | Active | ZINC14187636 | Decoy | ZINC42685266 | Decoy |
| 11452311 | Active | ZINC14196487 | Decoy | ZINC42689171 | Decoy |
| 11461951 | Active | ZINC14200149 | Decoy | ZINC42725009 | Decoy |
| 11610716 | Active | ZINC14245003 | Decoy | ZINC42739461 | Decoy |
| 11777650 | Active | ZINC14397141 | Decoy | ZINC42753415 | Decoy |
| 11857381 | Active | ZINC14429458 | Decoy | ZINC42761399 | Decoy |
| 11857382 | Active | ZINC14454624 | Decoy | ZINC42772226 | Decoy |
| 11858012 | Active | ZINC14534725 | Decoy | ZINC42862023 | Decoy |
| 11858013 | Active | ZINC14588459 | Decoy | ZINC42873099 | Decoy |
| 11858096 | Active | ZINC14614449 | Decoy | ZINC42874871 | Decoy |
| 11858098 | Active | ZINC14616985 | Decoy | ZINC42875063 | Decoy |
| 11858361 | Active | ZINC14638971 | Decoy | ZINC42875907 | Decoy |
| 15233292 | Active | ZINC14666445 | Decoy | ZINC42876010 | Decoy |
| 15233293 | Active | ZINC14737881 | Decoy | ZINC42883143 | Decoy |
| 15233294 | Active | ZINC14888364 | Decoy | ZINC42890707 | Decoy |
| 15233295 | Active | ZINC14944940 | Decoy | ZINC42905578 | Decoy |
| 15233300 | Active | ZINC14944956 | Decoy | ZINC42912469 | Decoy |
| 15233301 | Active | ZINC14947388 | Decoy | ZINC42921046 | Decoy |
| 16065132 | Active | ZINC14950416 | Decoy | ZINC42923340 | Decoy |
| 17757207 | Active | ZINC14950584 | Decoy | ZINC42928399 | Decoy |
| 17931612 | Active | ZINC14952229 | Decoy | ZINC42929423 | Decoy |
| 17962623 | Active | ZINC14953405 | Decoy | ZINC42939854 | Decoy |
| 17962624 | Active | ZINC14954644 | Decoy | ZINC42945377 | Decoy |
| 18314033 | Active | ZINC14959365 | Decoy | ZINC42954833 | Decoy |
| 18314034 | Active | ZINC14962933 | Decoy | ZINC42957057 | Decoy |
| 18314065 | Active | ZINC14964168 | Decoy | ZINC42977602 | Decoy |
| 18314087 | Active | ZINC14979857 | Decoy | ZINC42977782 | Decoy |
| 18314096 | Active | ZINC14983281 | Decoy | ZINC42985050 | Decoy |
| 18428373 | Active | ZINC14990342 | Decoy | ZINC42999322 | Decoy |
| 18428390 | Active | ZINC14991352 | Decoy | ZINC43023178 | Decoy |
| 18428396 | Active | ZINC15002303 | Decoy | ZINC43061206 | Decoy |
| 18452955 | Active | ZINC15017443 | Decoy | ZINC43062001 | Decoy |
| 18540050 | Active | ZINC15017502 | Decoy | ZINC43078913 | Decoy |
| 18540209 | Active | ZINC15067958 | Decoy | ZINC43080106 | Decoy |
| 18540224 | Active | ZINC15071464 | Decoy | ZINC43108404 | Decoy |
| 18540304 | Active | ZINC15071622 | Decoy | ZINC43114301 | Decoy |
| 19041233 | Active | ZINC15072388 | Decoy | ZINC43123550 | Decoy |
| 19593133 | Active | ZINC15076233 | Decoy | ZINC43149626 | Decoy |
| 19593144 | Active | ZINC15110156 | Decoy | ZINC43149633 | Decoy |
| 19593151 | Active | ZINC15121930 | Decoy | ZINC43162430 | Decoy |
| 19593173 | Active | ZINC15146057 | Decoy | ZINC43164492 | Decoy |
| 19593199 | Active | ZINC15173952 | Decoy | ZINC43177809 | Decoy |
| 20777079 | Active | ZINC15213320 | Decoy | ZINC43178546 | Decoy |
| 20803051 | Active | ZINC15224714 | Decoy | ZINC43186114 | Decoy |
| 20803095 | Active | ZINC15244296 | Decoy | ZINC43188530 | Decoy |
| 21036657 | Active | ZINC15247661 | Decoy | ZINC43237440 | Decoy |
| 21049034 | Active | ZINC15415217 | Decoy | ZINC43243133 | Decoy |
| 21049081 | Active | ZINC15416608 | Decoy | ZINC43259076 | Decoy |
| 21050106 | Active | ZINC15423157 | Decoy | ZINC43259629 | Decoy |
| 21050122 | Active | ZINC15423197 | Decoy | ZINC43281481 | Decoy |
| 21050315 | Active | ZINC15454458 | Decoy | ZINC43306412 | Decoy |
| 21883760 | Active | ZINC15454563 | Decoy | ZINC43307359 | Decoy |
| 21883763 | Active | ZINC15455280 | Decoy | ZINC43331207 | Decoy |
| 21964148 | Active | ZINC15455282 | Decoy | ZINC43333885 | Decoy |
| 21973858 | Active | ZINC15455514 | Decoy | ZINC43339793 | Decoy |
| 22081060 | Active | ZINC15455756 | Decoy | ZINC43341966 | Decoy |
| 22081061 | Active | ZINC15455780 | Decoy | ZINC43342725 | Decoy |
| 22081062 | Active | ZINC15455822 | Decoy | ZINC43358303 | Decoy |
| 22274410 | Active | ZINC15457909 | Decoy | ZINC43449181 | Decoy |
| 22274421 | Active | ZINC15457926 | Decoy | ZINC43450585 | Decoy |
| 22274550 | Active | ZINC15458042 | Decoy | ZINC43467435 | Decoy |
| 22274595 | Active | ZINC15503192 | Decoy | ZINC43467723 | Decoy |
| 22274683 | Active | ZINC15503470 | Decoy | ZINC43480975 | Decoy |
| 22274698 | Active | ZINC15539939 | Decoy | ZINC43485235 | Decoy |
| 22390950 | Active | ZINC15567185 | Decoy | ZINC43507733 | Decoy |
| 22450053 | Active | ZINC15581265 | Decoy | ZINC43507809 | Decoy |
| 22450062 | Active | ZINC15593240 | Decoy | ZINC43580392 | Decoy |
| 22450065 | Active | ZINC15625296 | Decoy | ZINC43598707 | Decoy |
| 22507777 | Active | ZINC15744941 | Decoy | ZINC43628152 | Decoy |
| 22507782 | Active | ZINC15780218 | Decoy | ZINC43634834 | Decoy |
| 22994521 | Active | ZINC15782714 | Decoy | ZINC43884333 | Decoy |
| 23378536 | Active | ZINC15784028 | Decoy | ZINC43885525 | Decoy |
| 23582465 | Active | ZINC15848197 | Decoy | ZINC43892448 | Decoy |
| 23582466 | Active | ZINC15937566 | Decoy | ZINC43900856 | Decoy |
| 44208899 | Active | ZINC15986101 | Decoy | ZINC43919029 | Decoy |
| 44211092 | Active | ZINC15988088 | Decoy | ZINC43936075 | Decoy |
| 44211339 | Active | ZINC16012905 | Decoy | ZINC43947477 | Decoy |
| 44211340 | Active | ZINC16043425 | Decoy | ZINC43953976 | Decoy |
| 44211341 | Active | ZINC16043736 | Decoy | ZINC43988245 | Decoy |
| 44211342 | Active | ZINC16049355 | Decoy | ZINC44000748 | Decoy |
| 44211343 | Active | ZINC16076170 | Decoy | ZINC44003461 | Decoy |
| 44211344 | Active | ZINC16102013 | Decoy | ZINC44008478 | Decoy |
| 44211345 | Active | ZINC16176696 | Decoy | ZINC44009355 | Decoy |
| 44213779 | Active | ZINC16184143 | Decoy | ZINC44022176 | Decoy |
| 44213780 | Active | ZINC16193593 | Decoy | ZINC44031947 | Decoy |
| 44214147 | Active | ZINC16211529 | Decoy | ZINC44034813 | Decoy |
| 44214148 | Active | ZINC16351915 | Decoy | ZINC44058889 | Decoy |
| 44214149 | Active | ZINC16362687 | Decoy | ZINC44062237 | Decoy |
| 44214150 | Active | ZINC16390198 | Decoy | ZINC44068097 | Decoy |
| 44214151 | Active | ZINC16578565 | Decoy | ZINC44070130 | Decoy |
| 44214152 | Active | ZINC16605957 | Decoy | ZINC44102340 | Decoy |
| 44214866 | Active | ZINC16606118 | Decoy | ZINC44102634 | Decoy |
| 44214867 | Active | ZINC16650802 | Decoy | ZINC44132900 | Decoy |
| 44215062 | Active | ZINC16695717 | Decoy | ZINC44132940 | Decoy |
| 44215079 | Active | ZINC16711540 | Decoy | ZINC44157077 | Decoy |
| 44215080 | Active | ZINC16848108 | Decoy | ZINC44158665 | Decoy |
| 44215081 | Active | ZINC16849771 | Decoy | ZINC44188981 | Decoy |
| 44215082 | Active | ZINC16967319 | Decoy | ZINC44193879 | Decoy |
| 44215174 | Active | ZINC16976243 | Decoy | ZINC44221067 | Decoy |
| 44215286 | Active | ZINC16982370 | Decoy | ZINC44228041 | Decoy |
| 44215287 | Active | ZINC16999469 | Decoy | ZINC44233474 | Decoy |
| 44215288 | Active | ZINC16999558 | Decoy | ZINC44233492 | Decoy |
| 44215289 | Active | ZINC17005188 | Decoy | ZINC44256870 | Decoy |
| 44215290 | Active | ZINC17008083 | Decoy | ZINC44264447 | Decoy |
| 44215291 | Active | ZINC17021302 | Decoy | ZINC44277388 | Decoy |
| 44215292 | Active | ZINC17021309 | Decoy | ZINC44281966 | Decoy |
| 44215409 | Active | ZINC17032263 | Decoy | ZINC44284165 | Decoy |
| 44215410 | Active | ZINC17046729 | Decoy | ZINC44293234 | Decoy |
| 44215411 | Active | ZINC17108407 | Decoy | ZINC44294262 | Decoy |
| 44215412 | Active | ZINC17121044 | Decoy | ZINC44301605 | Decoy |
| 44215477 | Active | ZINC17140333 | Decoy | ZINC44309890 | Decoy |
| 44215683 | Active | ZINC17175834 | Decoy | ZINC44386919 | Decoy |
| 44215684 | Active | ZINC17185667 | Decoy | ZINC44409327 | Decoy |
| 44215796 | Active | ZINC17191218 | Decoy | ZINC44520521 | Decoy |
| 44215798 | Active | ZINC17242628 | Decoy | ZINC44523877 | Decoy |
| 44216192 | Active | ZINC17250519 | Decoy | ZINC44524557 | Decoy |
| 44216193 | Active | ZINC17252858 | Decoy | ZINC44557057 | Decoy |
| 44216194 | Active | ZINC17300435 | Decoy | ZINC44557647 | Decoy |
| 44216195 | Active | ZINC17310647 | Decoy | ZINC44565521 | Decoy |
| 44216378 | Active | ZINC17314625 | Decoy | ZINC44590655 | Decoy |
| 44216386 | Active | ZINC17318650 | Decoy | ZINC44606670 | Decoy |
| 44216387 | Active | ZINC17322288 | Decoy | ZINC44615762 | Decoy |
| 44216396 | Active | ZINC17323063 | Decoy | ZINC44651308 | Decoy |
| 44216397 | Active | ZINC17324860 | Decoy | ZINC44656496 | Decoy |
| 44216398 | Active | ZINC17356223 | Decoy | ZINC44664146 | Decoy |
| 44216400 | Active | ZINC17378570 | Decoy | ZINC44706877 | Decoy |
| 44216401 | Active | ZINC17378585 | Decoy | ZINC44756048 | Decoy |
| ZINC00000023 | Decoy | ZINC17426791 | Decoy | ZINC44756233 | Decoy |
| ZINC00000148 | Decoy | ZINC17528870 | Decoy | ZINC44779080 | Decoy |
| ZINC00000166 | Decoy | ZINC17905722 | Decoy | ZINC44845238 | Decoy |
| ZINC00008813 | Decoy | ZINC17945958 | Decoy | ZINC44847997 | Decoy |
| ZINC00020783 | Decoy | ZINC17945988 | Decoy | ZINC44859574 | Decoy |
| ZINC00034383 | Decoy | ZINC18042650 | Decoy | ZINC44872123 | Decoy |
| ZINC00034553 | Decoy | ZINC18105909 | Decoy | ZINC44879946 | Decoy |
| ZINC00047900 | Decoy | ZINC18164313 | Decoy | ZINC44881570 | Decoy |
| ZINC00063586 | Decoy | ZINC18217634 | Decoy | ZINC44894810 | Decoy |
| ZINC00084527 | Decoy | ZINC18286724 | Decoy | ZINC44896053 | Decoy |
| ZINC00094212 | Decoy | ZINC18940224 | Decoy | ZINC44898336 | Decoy |
| ZINC00097630 | Decoy | ZINC19085011 | Decoy | ZINC45003965 | Decoy |
| ZINC00103402 | Decoy | ZINC19090251 | Decoy | ZINC45004032 | Decoy |
| ZINC00119867 | Decoy | ZINC19090436 | Decoy | ZINC45012954 | Decoy |
| ZINC00132466 | Decoy | ZINC19090972 | Decoy | ZINC45029976 | Decoy |
| ZINC00138395 | Decoy | ZINC19091065 | Decoy | ZINC45029984 | Decoy |
| ZINC00149980 | Decoy | ZINC19093491 | Decoy | ZINC45080994 | Decoy |
| ZINC00153788 | Decoy | ZINC19120558 | Decoy | ZINC45102927 | Decoy |
| ZINC00153936 | Decoy | ZINC19143320 | Decoy | ZINC45103981 | Decoy |
| ZINC00157085 | Decoy | ZINC19146093 | Decoy | ZINC45165404 | Decoy |
| ZINC00172185 | Decoy | ZINC19146919 | Decoy | ZINC45172724 | Decoy |
| ZINC00178685 | Decoy | ZINC19172867 | Decoy | ZINC45178225 | Decoy |
| ZINC00184165 | Decoy | ZINC19278260 | Decoy | ZINC45187065 | Decoy |
| ZINC00187266 | Decoy | ZINC19315504 | Decoy | ZINC45193892 | Decoy |
| ZINC00188967 | Decoy | ZINC19321759 | Decoy | ZINC45270673 | Decoy |
| ZINC00212522 | Decoy | ZINC19323336 | Decoy | ZINC45287243 | Decoy |
| ZINC00213461 | Decoy | ZINC19334714 | Decoy | ZINC45289770 | Decoy |
| ZINC00214740 | Decoy | ZINC19366098 | Decoy | ZINC45299987 | Decoy |
| ZINC00217286 | Decoy | ZINC19496377 | Decoy | ZINC45300263 | Decoy |
| ZINC00219944 | Decoy | ZINC19535638 | Decoy | ZINC45302122 | Decoy |
| ZINC00221775 | Decoy | ZINC19582941 | Decoy | ZINC45303059 | Decoy |
| ZINC00261867 | Decoy | ZINC19720668 | Decoy | ZINC45303129 | Decoy |
| ZINC00265845 | Decoy | ZINC19722424 | Decoy | ZINC45316153 | Decoy |
| ZINC00266651 | Decoy | ZINC19735517 | Decoy | ZINC45325131 | Decoy |
| ZINC00279890 | Decoy | ZINC19736888 | Decoy | ZINC45328854 | Decoy |
| ZINC00280451 | Decoy | ZINC19751533 | Decoy | ZINC45339217 | Decoy |
| ZINC00286753 | Decoy | ZINC19751540 | Decoy | ZINC45354126 | Decoy |
| ZINC00291526 | Decoy | ZINC19781722 | Decoy | ZINC45356323 | Decoy |
| ZINC00292232 | Decoy | ZINC19792143 | Decoy | ZINC45357215 | Decoy |
| ZINC00304758 | Decoy | ZINC19794942 | Decoy | ZINC45358314 | Decoy |
| ZINC00312604 | Decoy | ZINC19795292 | Decoy | ZINC45358554 | Decoy |
| ZINC00331121 | Decoy | ZINC19801351 | Decoy | ZINC45364581 | Decoy |
| ZINC00334157 | Decoy | ZINC19802513 | Decoy | ZINC45364958 | Decoy |
| ZINC00336573 | Decoy | ZINC19808986 | Decoy | ZINC45366935 | Decoy |
| ZINC00348384 | Decoy | ZINC19816838 | Decoy | ZINC45408370 | Decoy |
| ZINC00348834 | Decoy | ZINC19842329 | Decoy | ZINC45458718 | Decoy |
| ZINC00351212 | Decoy | ZINC19848413 | Decoy | ZINC45464609 | Decoy |
| ZINC00354096 | Decoy | ZINC19853818 | Decoy | ZINC45471379 | Decoy |
| ZINC00388260 | Decoy | ZINC19878417 | Decoy | ZINC45473141 | Decoy |
| ZINC00388341 | Decoy | ZINC19893281 | Decoy | ZINC45484570 | Decoy |
| ZINC00391151 | Decoy | ZINC19909514 | Decoy | ZINC45518922 | Decoy |
| ZINC00391249 | Decoy | ZINC19909801 | Decoy | ZINC45570214 | Decoy |
| ZINC00394084 | Decoy | ZINC19932295 | Decoy | ZINC45576663 | Decoy |
| ZINC00395646 | Decoy | ZINC19937432 | Decoy | ZINC45620945 | Decoy |
| ZINC00397417 | Decoy | ZINC19937474 | Decoy | ZINC45622706 | Decoy |
| ZINC00397477 | Decoy | ZINC19944590 | Decoy | ZINC45671351 | Decoy |
| ZINC00402558 | Decoy | ZINC19960630 | Decoy | ZINC45744750 | Decoy |
| ZINC00403284 | Decoy | ZINC20014790 | Decoy | ZINC45751468 | Decoy |
| ZINC00403380 | Decoy | ZINC20035362 | Decoy | ZINC45751635 | Decoy |
| ZINC00405577 | Decoy | ZINC20054851 | Decoy | ZINC45766383 | Decoy |
| ZINC00409349 | Decoy | ZINC20058135 | Decoy | ZINC45769763 | Decoy |
| ZINC00456231 | Decoy | ZINC20070407 | Decoy | ZINC45769819 | Decoy |
| ZINC00461911 | Decoy | ZINC20084805 | Decoy | ZINC45800995 | Decoy |
| ZINC00482631 | Decoy | ZINC20118551 | Decoy | ZINC45806525 | Decoy |
| ZINC00493890 | Decoy | ZINC20129580 | Decoy | ZINC45806546 | Decoy |
| ZINC00587287 | Decoy | ZINC20131159 | Decoy | ZINC45807106 | Decoy |
| ZINC00590702 | Decoy | ZINC20149992 | Decoy | ZINC45807132 | Decoy |
| ZINC00652176 | Decoy | ZINC20158471 | Decoy | ZINC45810226 | Decoy |
| ZINC00718219 | Decoy | ZINC20183209 | Decoy | ZINC45810376 | Decoy |
| ZINC00718486 | Decoy | ZINC20188797 | Decoy | ZINC45832023 | Decoy |
| ZINC00757802 | Decoy | ZINC20202927 | Decoy | ZINC45836401 | Decoy |
| ZINC00783079 | Decoy | ZINC20203480 | Decoy | ZINC45838687 | Decoy |
| ZINC00801785 | Decoy | ZINC20208296 | Decoy | ZINC45840912 | Decoy |
| ZINC00820629 | Decoy | ZINC20230859 | Decoy | ZINC45875993 | Decoy |
| ZINC00865173 | Decoy | ZINC20240223 | Decoy | ZINC45903200 | Decoy |
| ZINC00865174 | Decoy | ZINC20261246 | Decoy | ZINC45926141 | Decoy |
| ZINC00865219 | Decoy | ZINC20293496 | Decoy | ZINC45947084 | Decoy |
| ZINC00901908 | Decoy | ZINC20305929 | Decoy | ZINC45950559 | Decoy |
| ZINC00993624 | Decoy | ZINC20349556 | Decoy | ZINC45951109 | Decoy |
| ZINC01001887 | Decoy | ZINC20349558 | Decoy | ZINC45960014 | Decoy |
| ZINC01060931 | Decoy | ZINC20349714 | Decoy | ZINC45964812 | Decoy |
| ZINC01072041 | Decoy | ZINC20359232 | Decoy | ZINC45965421 | Decoy |
| ZINC01157793 | Decoy | ZINC20360858 | Decoy | ZINC45979706 | Decoy |
| ZINC01173579 | Decoy | ZINC20378264 | Decoy | ZINC45987099 | Decoy |
| ZINC01188886 | Decoy | ZINC20382763 | Decoy | ZINC45987720 | Decoy |
| ZINC01217464 | Decoy | ZINC20385670 | Decoy | ZINC45991737 | Decoy |
| ZINC01225298 | Decoy | ZINC20389446 | Decoy | ZINC46000564 | Decoy |
| ZINC01227105 | Decoy | ZINC20437328 | Decoy | ZINC46007958 | Decoy |
| ZINC01230065 | Decoy | ZINC20447656 | Decoy | ZINC46017184 | Decoy |
| ZINC01393292 | Decoy | ZINC20462769 | Decoy | ZINC46025385 | Decoy |
| ZINC01399042 | Decoy | ZINC20468557 | Decoy | ZINC46034095 | Decoy |
| ZINC01427185 | Decoy | ZINC20496247 | Decoy | ZINC46056789 | Decoy |
| ZINC01428922 | Decoy | ZINC20498352 | Decoy | ZINC46064541 | Decoy |
| ZINC01442833 | Decoy | ZINC20522765 | Decoy | ZINC46066186 | Decoy |
| ZINC01442844 | Decoy | ZINC20550835 | Decoy | ZINC46106915 | Decoy |
| ZINC01443055 | Decoy | ZINC20582663 | Decoy | ZINC46107686 | Decoy |
| ZINC01443072 | Decoy | ZINC20583109 | Decoy | ZINC46112434 | Decoy |
| ZINC01489493 | Decoy | ZINC20680810 | Decoy | ZINC46153081 | Decoy |
| ZINC01498114 | Decoy | ZINC20760944 | Decoy | ZINC46153087 | Decoy |
| ZINC01529130 | Decoy | ZINC20807346 | Decoy | ZINC46164672 | Decoy |
| ZINC01555488 | Decoy | ZINC20874262 | Decoy | ZINC46189737 | Decoy |
| ZINC01555876 | Decoy | ZINC20876169 | Decoy | ZINC46231185 | Decoy |
| ZINC01557575 | Decoy | ZINC20887759 | Decoy | ZINC46347069 | Decoy |
| ZINC01567972 | Decoy | ZINC20917771 | Decoy | ZINC46386848 | Decoy |
| ZINC01569514 | Decoy | ZINC20931049 | Decoy | ZINC46402573 | Decoy |
| ZINC01571099 | Decoy | ZINC20935486 | Decoy | ZINC46407103 | Decoy |
| ZINC01575389 | Decoy | ZINC20976089 | Decoy | ZINC46415808 | Decoy |
| ZINC01577272 | Decoy | ZINC20993526 | Decoy | ZINC46416450 | Decoy |
| ZINC01577758 | Decoy | ZINC21020010 | Decoy | ZINC46438197 | Decoy |
| ZINC01577874 | Decoy | ZINC21025791 | Decoy | ZINC46444389 | Decoy |
| ZINC01585007 | Decoy | ZINC21134220 | Decoy | ZINC46455660 | Decoy |
| ZINC01594964 | Decoy | ZINC21266503 | Decoy | ZINC46456771 | Decoy |
| ZINC01596313 | Decoy | ZINC21271909 | Decoy | ZINC46467541 | Decoy |
| ZINC01600247 | Decoy | ZINC21272498 | Decoy | ZINC46484202 | Decoy |
| ZINC01605247 | Decoy | ZINC21350957 | Decoy | ZINC46497236 | Decoy |
| ZINC01608679 | Decoy | ZINC21374076 | Decoy | ZINC46500604 | Decoy |
| ZINC01608710 | Decoy | ZINC21443671 | Decoy | ZINC46503162 | Decoy |
| ZINC01609924 | Decoy | ZINC21524704 | Decoy | ZINC46503371 | Decoy |
| ZINC01612993 | Decoy | ZINC21533567 | Decoy | ZINC46520411 | Decoy |
| ZINC01613267 | Decoy | ZINC21580602 | Decoy | ZINC46532407 | Decoy |
| ZINC01614062 | Decoy | ZINC21586880 | Decoy | ZINC46575697 | Decoy |
| ZINC01614974 | Decoy | ZINC21663595 | Decoy | ZINC46598066 | Decoy |
| ZINC01616549 | Decoy | ZINC21689771 | Decoy | ZINC46608855 | Decoy |
| ZINC01625756 | Decoy | ZINC21690148 | Decoy | ZINC46630029 | Decoy |
| ZINC01632261 | Decoy | ZINC21712244 | Decoy | ZINC46639502 | Decoy |
| ZINC01642804 | Decoy | ZINC21792267 | Decoy | ZINC46654074 | Decoy |
| ZINC01646691 | Decoy | ZINC21818937 | Decoy | ZINC46674186 | Decoy |
| ZINC01656257 | Decoy | ZINC21884994 | Decoy | ZINC46674939 | Decoy |
| ZINC01660014 | Decoy | ZINC21888913 | Decoy | ZINC46717029 | Decoy |
| ZINC01664003 | Decoy | ZINC21953239 | Decoy | ZINC46717145 | Decoy |
| ZINC01664575 | Decoy | ZINC21966192 | Decoy | ZINC46726362 | Decoy |
| ZINC01665083 | Decoy | ZINC21966286 | Decoy | ZINC46730805 | Decoy |
| ZINC01665565 | Decoy | ZINC21984899 | Decoy | ZINC46730898 | Decoy |
| ZINC01667056 | Decoy | ZINC21986328 | Decoy | ZINC46773582 | Decoy |
| ZINC01669058 | Decoy | ZINC21994542 | Decoy | ZINC46783035 | Decoy |
| ZINC01669841 | Decoy | ZINC21994545 | Decoy | ZINC46819453 | Decoy |
| ZINC01669842 | Decoy | ZINC21998527 | Decoy | ZINC46833498 | Decoy |
| ZINC01670708 | Decoy | ZINC21998596 | Decoy | ZINC46836776 | Decoy |
| ZINC01672466 | Decoy | ZINC22001613 | Decoy | ZINC46836822 | Decoy |
| ZINC01672529 | Decoy | ZINC22002586 | Decoy | ZINC46836850 | Decoy |
| ZINC01672753 | Decoy | ZINC22006423 | Decoy | ZINC46836873 | Decoy |
| ZINC01672807 | Decoy | ZINC22010950 | Decoy | ZINC46862447 | Decoy |
| ZINC01673070 | Decoy | ZINC22011278 | Decoy | ZINC46871969 | Decoy |
| ZINC01679500 | Decoy | ZINC22015037 | Decoy | ZINC46872203 | Decoy |
| ZINC01679805 | Decoy | ZINC22017282 | Decoy | ZINC46973317 | Decoy |
| ZINC01687533 | Decoy | ZINC22034656 | Decoy | ZINC47008861 | Decoy |
| ZINC01688268 | Decoy | ZINC22038503 | Decoy | ZINC47023605 | Decoy |
| ZINC01690604 | Decoy | ZINC22052510 | Decoy | ZINC47024339 | Decoy |
| ZINC01698506 | Decoy | ZINC22052629 | Decoy | ZINC47096913 | Decoy |
| ZINC01706270 | Decoy | ZINC22053222 | Decoy | ZINC47110645 | Decoy |
| ZINC01710462 | Decoy | ZINC22060336 | Decoy | ZINC47136513 | Decoy |
| ZINC01710682 | Decoy | ZINC22073641 | Decoy | ZINC47142104 | Decoy |
| ZINC01712668 | Decoy | ZINC22073767 | Decoy | ZINC47186776 | Decoy |
| ZINC01713519 | Decoy | ZINC22095735 | Decoy | ZINC47197808 | Decoy |
| ZINC01716704 | Decoy | ZINC22101997 | Decoy | ZINC47220178 | Decoy |
| ZINC01716845 | Decoy | ZINC22130849 | Decoy | ZINC47220761 | Decoy |
| ZINC01718487 | Decoy | ZINC22132028 | Decoy | ZINC47241552 | Decoy |
| ZINC01721654 | Decoy | ZINC22167243 | Decoy | ZINC47242527 | Decoy |
| ZINC01724321 | Decoy | ZINC22215392 | Decoy | ZINC47250495 | Decoy |
| ZINC01726269 | Decoy | ZINC22262387 | Decoy | ZINC47257592 | Decoy |
| ZINC01730911 | Decoy | ZINC22309735 | Decoy | ZINC47258173 | Decoy |
| ZINC01731835 | Decoy | ZINC22314046 | Decoy | ZINC47269853 | Decoy |
| ZINC01736017 | Decoy | ZINC22354640 | Decoy | ZINC47279509 | Decoy |
| ZINC01740613 | Decoy | ZINC22402483 | Decoy | ZINC47284290 | Decoy |
| ZINC01743172 | Decoy | ZINC22403644 | Decoy | ZINC47285713 | Decoy |
| ZINC01748272 | Decoy | ZINC22404591 | Decoy | ZINC47288087 | Decoy |
| ZINC01748630 | Decoy | ZINC22450200 | Decoy | ZINC47297480 | Decoy |
| ZINC01749402 | Decoy | ZINC22450677 | Decoy | ZINC47337456 | Decoy |
| ZINC01752317 | Decoy | ZINC22462859 | Decoy | ZINC47425175 | Decoy |
| ZINC01758225 | Decoy | ZINC22466970 | Decoy | ZINC47460171 | Decoy |
| ZINC01758668 | Decoy | ZINC22468849 | Decoy | ZINC47460219 | Decoy |
| ZINC01764666 | Decoy | ZINC22506878 | Decoy | ZINC47460482 | Decoy |
| ZINC01808774 | Decoy | ZINC22580491 | Decoy | ZINC47486490 | Decoy |
| ZINC01810730 | Decoy | ZINC22686330 | Decoy | ZINC47489781 | Decoy |
| ZINC01820472 | Decoy | ZINC22691349 | Decoy | ZINC47496540 | Decoy |
| ZINC01823278 | Decoy | ZINC22733839 | Decoy | ZINC47502270 | Decoy |
| ZINC01842986 | Decoy | ZINC22733887 | Decoy | ZINC47502482 | Decoy |
| ZINC01848265 | Decoy | ZINC22734132 | Decoy | ZINC47509346 | Decoy |
| ZINC01872360 | Decoy | ZINC22765573 | Decoy | ZINC47510438 | Decoy |
| ZINC01872979 | Decoy | ZINC22777335 | Decoy | ZINC47510439 | Decoy |
| ZINC01882115 | Decoy | ZINC22786844 | Decoy | ZINC47511412 | Decoy |
| ZINC01883394 | Decoy | ZINC22787524 | Decoy | ZINC47535660 | Decoy |
| ZINC01994163 | Decoy | ZINC22787560 | Decoy | ZINC47557678 | Decoy |
| ZINC02030029 | Decoy | ZINC22793306 | Decoy | ZINC47577712 | Decoy |
| ZINC02044831 | Decoy | ZINC22795160 | Decoy | ZINC47600928 | Decoy |
| ZINC02045287 | Decoy | ZINC22804242 | Decoy | ZINC47604280 | Decoy |
| ZINC02046519 | Decoy | ZINC22804427 | Decoy | ZINC47608155 | Decoy |
| ZINC02050254 | Decoy | ZINC22804841 | Decoy | ZINC47612019 | Decoy |
| ZINC02050457 | Decoy | ZINC22804866 | Decoy | ZINC47614060 | Decoy |
| ZINC02065087 | Decoy | ZINC22811268 | Decoy | ZINC47616783 | Decoy |
| ZINC02073198 | Decoy | ZINC22831292 | Decoy | ZINC47623653 | Decoy |
| ZINC02077724 | Decoy | ZINC22847542 | Decoy | ZINC47623870 | Decoy |
| ZINC02080287 | Decoy | ZINC22872519 | Decoy | ZINC47628545 | Decoy |
| ZINC02096149 | Decoy | ZINC22920890 | Decoy | ZINC47669510 | Decoy |
| ZINC02103096 | Decoy | ZINC22922527 | Decoy | ZINC47676306 | Decoy |
| ZINC02208784 | Decoy | ZINC22926666 | Decoy | ZINC47680885 | Decoy |
| ZINC02222202 | Decoy | ZINC22926752 | Decoy | ZINC47697701 | Decoy |
| ZINC02237350 | Decoy | ZINC22936268 | Decoy | ZINC47705967 | Decoy |
| ZINC02248377 | Decoy | ZINC22936379 | Decoy | ZINC47707343 | Decoy |
| ZINC02272476 | Decoy | ZINC22940925 | Decoy | ZINC47725706 | Decoy |
| ZINC02288976 | Decoy | ZINC22976361 | Decoy | ZINC47744262 | Decoy |
| ZINC02384438 | Decoy | ZINC22999921 | Decoy | ZINC47746442 | Decoy |
| ZINC02475149 | Decoy | ZINC23080870 | Decoy | ZINC47748347 | Decoy |
| ZINC02478477 | Decoy | ZINC23105935 | Decoy | ZINC47771671 | Decoy |
| ZINC02481878 | Decoy | ZINC23115569 | Decoy | ZINC47776234 | Decoy |
| ZINC02485570 | Decoy | ZINC23128944 | Decoy | ZINC47777950 | Decoy |
| ZINC02494891 | Decoy | ZINC23197194 | Decoy | ZINC47794304 | Decoy |
| ZINC02510147 | Decoy | ZINC23326219 | Decoy | ZINC47808964 | Decoy |
| ZINC02522357 | Decoy | ZINC23337589 | Decoy | ZINC47830871 | Decoy |
| ZINC02530239 | Decoy | ZINC23355141 | Decoy | ZINC47831709 | Decoy |
| ZINC02530902 | Decoy | ZINC23392079 | Decoy | ZINC47845164 | Decoy |
| ZINC02537763 | Decoy | ZINC23468160 | Decoy | ZINC47868013 | Decoy |
| ZINC02537938 | Decoy | ZINC23491684 | Decoy | ZINC47870663 | Decoy |
| ZINC02547559 | Decoy | ZINC23543949 | Decoy | ZINC47871135 | Decoy |
| ZINC02556440 | Decoy | ZINC23547578 | Decoy | ZINC47871285 | Decoy |
| ZINC02559429 | Decoy | ZINC23899438 | Decoy | ZINC47898759 | Decoy |
| ZINC02565330 | Decoy | ZINC23962334 | Decoy | ZINC47902859 | Decoy |
| ZINC02566174 | Decoy | ZINC23989445 | Decoy | ZINC47917192 | Decoy |
| ZINC02569411 | Decoy | ZINC24151668 | Decoy | ZINC47932252 | Decoy |
| ZINC02573846 | Decoy | ZINC24155615 | Decoy | ZINC47934188 | Decoy |
| ZINC02573849 | Decoy | ZINC24155711 | Decoy | ZINC47951028 | Decoy |
| ZINC02583625 | Decoy | ZINC24157628 | Decoy | ZINC47968232 | Decoy |
| ZINC02651849 | Decoy | ZINC24205778 | Decoy | ZINC47969149 | Decoy |
| ZINC02652779 | Decoy | ZINC24231297 | Decoy | ZINC47969974 | Decoy |
| ZINC02675058 | Decoy | ZINC24566613 | Decoy | ZINC47971857 | Decoy |
| ZINC02675509 | Decoy | ZINC24614920 | Decoy | ZINC47972356 | Decoy |
| ZINC02684618 | Decoy | ZINC24662145 | Decoy | ZINC47973484 | Decoy |
| ZINC02694590 | Decoy | ZINC24708383 | Decoy | ZINC47977837 | Decoy |
| ZINC02695814 | Decoy | ZINC24730234 | Decoy | ZINC47991181 | Decoy |
| ZINC02721978 | Decoy | ZINC24810877 | Decoy | ZINC48008696 | Decoy |
| ZINC02729128 | Decoy | ZINC24852382 | Decoy | ZINC48037867 | Decoy |
| ZINC02741580 | Decoy | ZINC24872172 | Decoy | ZINC48040792 | Decoy |
| ZINC02741615 | Decoy | ZINC24908443 | Decoy | ZINC48040860 | Decoy |
| ZINC02744800 | Decoy | ZINC24915942 | Decoy | ZINC48045223 | Decoy |
| ZINC02748286 | Decoy | ZINC24985705 | Decoy | ZINC48056195 | Decoy |
| ZINC02752437 | Decoy | ZINC24991083 | Decoy | ZINC48056386 | Decoy |
| ZINC02753768 | Decoy | ZINC24992548 | Decoy | ZINC48060815 | Decoy |
| ZINC02760495 | Decoy | ZINC25073913 | Decoy | ZINC48067249 | Decoy |
| ZINC02761713 | Decoy | ZINC25195831 | Decoy | ZINC48070815 | Decoy |
| ZINC02796180 | Decoy | ZINC25307306 | Decoy | ZINC48072550 | Decoy |
| ZINC02810909 | Decoy | ZINC25342435 | Decoy | ZINC48075720 | Decoy |
| ZINC02842456 | Decoy | ZINC25417254 | Decoy | ZINC48076418 | Decoy |
| ZINC02845357 | Decoy | ZINC25425479 | Decoy | ZINC48076508 | Decoy |
| ZINC02857555 | Decoy | ZINC25445819 | Decoy | ZINC48082018 | Decoy |
| ZINC02877529 | Decoy | ZINC25460293 | Decoy | ZINC48096227 | Decoy |
| ZINC02882615 | Decoy | ZINC25762295 | Decoy | ZINC48098842 | Decoy |
| ZINC02899007 | Decoy | ZINC25809881 | Decoy | ZINC48099550 | Decoy |
| ZINC02928686 | Decoy | ZINC25903893 | Decoy | ZINC48129878 | Decoy |
| ZINC02943972 | Decoy | ZINC25986437 | Decoy | ZINC48132098 | Decoy |
| ZINC02981815 | Decoy | ZINC26121156 | Decoy | ZINC48153258 | Decoy |
| ZINC02990104 | Decoy | ZINC26142556 | Decoy | ZINC48167566 | Decoy |
| ZINC03018982 | Decoy | ZINC26266935 | Decoy | ZINC48167751 | Decoy |
| ZINC03023954 | Decoy | ZINC26285052 | Decoy | ZINC48175598 | Decoy |
| ZINC03028191 | Decoy | ZINC26294803 | Decoy | ZINC48184140 | Decoy |
| ZINC03055028 | Decoy | ZINC26298761 | Decoy | ZINC48185889 | Decoy |
| ZINC03103953 | Decoy | ZINC26307611 | Decoy | ZINC48186438 | Decoy |
| ZINC03122472 | Decoy | ZINC26321779 | Decoy | ZINC48193603 | Decoy |
| ZINC03147394 | Decoy | ZINC26371458 | Decoy | ZINC48195077 | Decoy |
| ZINC03160150 | Decoy | ZINC26377161 | Decoy | ZINC48195143 | Decoy |
| ZINC03161233 | Decoy | ZINC26383778 | Decoy | ZINC48204986 | Decoy |
| ZINC03165501 | Decoy | ZINC26402222 | Decoy | ZINC48204999 | Decoy |
| ZINC03179695 | Decoy | ZINC26470666 | Decoy | ZINC48211392 | Decoy |
| ZINC03181372 | Decoy | ZINC26509097 | Decoy | ZINC48215373 | Decoy |
| ZINC03204810 | Decoy | ZINC26578140 | Decoy | ZINC48219174 | Decoy |
| ZINC03225781 | Decoy | ZINC26621696 | Decoy | ZINC48237042 | Decoy |
| ZINC03261788 | Decoy | ZINC26652489 | Decoy | ZINC48237412 | Decoy |
| ZINC03266151 | Decoy | ZINC26655396 | Decoy | ZINC48242389 | Decoy |
| ZINC03268733 | Decoy | ZINC26665008 | Decoy | ZINC48244559 | Decoy |
| ZINC03308006 | Decoy | ZINC26672802 | Decoy | ZINC48244953 | Decoy |
| ZINC03308250 | Decoy | ZINC26712266 | Decoy | ZINC48274494 | Decoy |
| ZINC03312221 | Decoy | ZINC26719574 | Decoy | ZINC48279557 | Decoy |
| ZINC03387579 | Decoy | ZINC26725960 | Decoy | ZINC48297995 | Decoy |
| ZINC03473720 | Decoy | ZINC26741344 | Decoy | ZINC48298014 | Decoy |
| ZINC03515172 | Decoy | ZINC26744163 | Decoy | ZINC48298677 | Decoy |
| ZINC03638519 | Decoy | ZINC26749760 | Decoy | ZINC48314153 | Decoy |
| ZINC03666355 | Decoy | ZINC26775757 | Decoy | ZINC48314607 | Decoy |
| ZINC03678305 | Decoy | ZINC26776085 | Decoy | ZINC48314838 | Decoy |
| ZINC03683487 | Decoy | ZINC26781314 | Decoy | ZINC48315203 | Decoy |
| ZINC03684095 | Decoy | ZINC26850663 | Decoy | ZINC48321359 | Decoy |
| ZINC03684447 | Decoy | ZINC26853650 | Decoy | ZINC48321785 | Decoy |
| ZINC03705647 | Decoy | ZINC26866121 | Decoy | ZINC48348601 | Decoy |
| ZINC03794812 | Decoy | ZINC26897771 | Decoy | ZINC48360117 | Decoy |
| ZINC03845104 | Decoy | ZINC26897905 | Decoy | ZINC48362154 | Decoy |
| ZINC03845591 | Decoy | ZINC26897963 | Decoy | ZINC48376960 | Decoy |
| ZINC03845880 | Decoy | ZINC26898079 | Decoy | ZINC48377415 | Decoy |
| ZINC03846093 | Decoy | ZINC26913500 | Decoy | ZINC48380773 | Decoy |
| ZINC03861241 | Decoy | ZINC26940305 | Decoy | ZINC48383066 | Decoy |
| ZINC03872049 | Decoy | ZINC26940373 | Decoy | ZINC48415587 | Decoy |
| ZINC03873371 | Decoy | ZINC26990470 | Decoy | ZINC48419126 | Decoy |
| ZINC03877504 | Decoy | ZINC26997676 | Decoy | ZINC48438778 | Decoy |
| ZINC03877758 | Decoy | ZINC27010626 | Decoy | ZINC48439470 | Decoy |
| ZINC03877833 | Decoy | ZINC27024551 | Decoy | ZINC48442765 | Decoy |
| ZINC03880139 | Decoy | ZINC27030140 | Decoy | ZINC48443959 | Decoy |
| ZINC03885292 | Decoy | ZINC27055217 | Decoy | ZINC48469557 | Decoy |
| ZINC03888139 | Decoy | ZINC27101372 | Decoy | ZINC48469573 | Decoy |
| ZINC03888524 | Decoy | ZINC27106308 | Decoy | ZINC48493711 | Decoy |
| ZINC03888832 | Decoy | ZINC27185277 | Decoy | ZINC48502142 | Decoy |
| ZINC03911855 | Decoy | ZINC27187515 | Decoy | ZINC48507361 | Decoy |
| ZINC03929274 | Decoy | ZINC27188057 | Decoy | ZINC48539785 | Decoy |
| ZINC03929879 | Decoy | ZINC27209383 | Decoy | ZINC48562779 | Decoy |
| ZINC03953524 | Decoy | ZINC27212320 | Decoy | ZINC48563272 | Decoy |
| ZINC03956330 | Decoy | ZINC27235091 | Decoy | ZINC48563297 | Decoy |
| ZINC03960913 | Decoy | ZINC27235139 | Decoy | ZINC48563419 | Decoy |
| ZINC04005307 | Decoy | ZINC27241461 | Decoy | ZINC48563648 | Decoy |
| ZINC04015451 | Decoy | ZINC27287392 | Decoy | ZINC48564506 | Decoy |
| ZINC04045567 | Decoy | ZINC27300516 | Decoy | ZINC48576383 | Decoy |
| ZINC04050495 | Decoy | ZINC27306883 | Decoy | ZINC48580008 | Decoy |
| ZINC04050531 | Decoy | ZINC27318561 | Decoy | ZINC48586317 | Decoy |
| ZINC04065096 | Decoy | ZINC27319374 | Decoy | ZINC48614073 | Decoy |
| ZINC04076194 | Decoy | ZINC27327839 | Decoy | ZINC48614581 | Decoy |
| ZINC04089586 | Decoy | ZINC27328238 | Decoy | ZINC48614654 | Decoy |
| ZINC04090524 | Decoy | ZINC27330665 | Decoy | ZINC48615613 | Decoy |
| ZINC04107140 | Decoy | ZINC27367720 | Decoy | ZINC48617504 | Decoy |
| ZINC04107222 | Decoy | ZINC27378842 | Decoy | ZINC48618578 | Decoy |
| ZINC04131211 | Decoy | ZINC27517993 | Decoy | ZINC48618606 | Decoy |
| ZINC04142586 | Decoy | ZINC27518111 | Decoy | ZINC48620826 | Decoy |
| ZINC04144116 | Decoy | ZINC27518628 | Decoy | ZINC48623571 | Decoy |
| ZINC04170372 | Decoy | ZINC27519643 | Decoy | ZINC48628591 | Decoy |
| ZINC04178403 | Decoy | ZINC27526162 | Decoy | ZINC48652581 | Decoy |
| ZINC04180621 | Decoy | ZINC27526809 | Decoy | ZINC48653525 | Decoy |
| ZINC04183230 | Decoy | ZINC27550372 | Decoy | ZINC48667248 | Decoy |
| ZINC04192231 | Decoy | ZINC27556030 | Decoy | ZINC48672752 | Decoy |
| ZINC04200113 | Decoy | ZINC27556758 | Decoy | ZINC48680804 | Decoy |
| ZINC04203200 | Decoy | ZINC27558295 | Decoy | ZINC48682350 | Decoy |
| ZINC04203518 | Decoy | ZINC27563870 | Decoy | ZINC48683512 | Decoy |
| ZINC04203583 | Decoy | ZINC27600424 | Decoy | ZINC48684536 | Decoy |
| ZINC04203854 | Decoy | ZINC27630204 | Decoy | ZINC48684660 | Decoy |
| ZINC04230513 | Decoy | ZINC27642069 | Decoy | ZINC48686226 | Decoy |
| ZINC04230940 | Decoy | ZINC27643868 | Decoy | ZINC48687675 | Decoy |
| ZINC04237325 | Decoy | ZINC27656111 | Decoy | ZINC48688652 | Decoy |
| ZINC04237620 | Decoy | ZINC27781081 | Decoy | ZINC48693897 | Decoy |
| ZINC04247090 | Decoy | ZINC27867903 | Decoy | ZINC48693898 | Decoy |
| ZINC04262061 | Decoy | ZINC27899162 | Decoy | ZINC48695472 | Decoy |
| ZINC04262062 | Decoy | ZINC27926623 | Decoy | ZINC48695696 | Decoy |
| ZINC04262682 | Decoy | ZINC27995935 | Decoy | ZINC48702614 | Decoy |
| ZINC04284292 | Decoy | ZINC28019255 | Decoy | ZINC48707779 | Decoy |
| ZINC04286309 | Decoy | ZINC28021799 | Decoy | ZINC48707812 | Decoy |
| ZINC04286614 | Decoy | ZINC28086146 | Decoy | ZINC48709533 | Decoy |
| ZINC04290973 | Decoy | ZINC28109578 | Decoy | ZINC48710193 | Decoy |
| ZINC04306612 | Decoy | ZINC28115607 | Decoy | ZINC48710239 | Decoy |
| ZINC04312900 | Decoy | ZINC28123369 | Decoy | ZINC48710956 | Decoy |
| ZINC04319919 | Decoy | ZINC28173635 | Decoy | ZINC48711279 | Decoy |
| ZINC04324701 | Decoy | ZINC28220100 | Decoy | ZINC48711589 | Decoy |
| ZINC04333581 | Decoy | ZINC28222301 | Decoy | ZINC48712673 | Decoy |
| ZINC04338253 | Decoy | ZINC28234343 | Decoy | ZINC48713377 | Decoy |
| ZINC04347635 | Decoy | ZINC28246294 | Decoy | ZINC48714031 | Decoy |
| ZINC04383012 | Decoy | ZINC28259040 | Decoy | ZINC48714078 | Decoy |
| ZINC04386894 | Decoy | ZINC28284819 | Decoy | ZINC48714199 | Decoy |
| ZINC04395071 | Decoy | ZINC28343837 | Decoy | ZINC48714225 | Decoy |
| ZINC04402445 | Decoy | ZINC28388885 | Decoy | ZINC48714230 | Decoy |
| ZINC04406306 | Decoy | ZINC28399010 | Decoy | ZINC48717109 | Decoy |
| ZINC04420404 | Decoy | ZINC28428315 | Decoy | ZINC48734848 | Decoy |
| ZINC04458700 | Decoy | ZINC28506108 | Decoy | ZINC48741466 | Decoy |
| ZINC04498691 | Decoy | ZINC28527423 | Decoy | ZINC48745430 | Decoy |
| ZINC04499382 | Decoy | ZINC28527452 | Decoy | ZINC48747575 | Decoy |
| ZINC04548040 | Decoy | ZINC28528046 | Decoy | ZINC48747959 | Decoy |
| ZINC04559556 | Decoy | ZINC28573153 | Decoy | ZINC48748888 | Decoy |
| ZINC04562390 | Decoy | ZINC28604105 | Decoy | ZINC48748896 | Decoy |
| ZINC04568379 | Decoy | ZINC28635866 | Decoy | ZINC48753336 | Decoy |
| ZINC04576157 | Decoy | ZINC28642663 | Decoy | ZINC48761454 | Decoy |
| ZINC04600498 | Decoy | ZINC28653510 | Decoy | ZINC48772910 | Decoy |
| ZINC04614977 | Decoy | ZINC28663447 | Decoy | ZINC48776426 | Decoy |
| ZINC04616539 | Decoy | ZINC28709135 | Decoy | ZINC48777623 | Decoy |
| ZINC04656483 | Decoy | ZINC28713795 | Decoy | ZINC48779541 | Decoy |
| ZINC04660496 | Decoy | ZINC28714190 | Decoy | ZINC48780459 | Decoy |
| ZINC04661708 | Decoy | ZINC28714563 | Decoy | ZINC48780673 | Decoy |
| ZINC04670550 | Decoy | ZINC28715391 | Decoy | ZINC48792129 | Decoy |
| ZINC04675340 | Decoy | ZINC28715412 | Decoy | ZINC48801218 | Decoy |
| ZINC04684471 | Decoy | ZINC28766870 | Decoy | ZINC48805715 | Decoy |
| ZINC04687525 | Decoy | ZINC28768124 | Decoy | ZINC48808752 | Decoy |
| ZINC04688215 | Decoy | ZINC28790433 | Decoy | ZINC48809750 | Decoy |
| ZINC04690453 | Decoy | ZINC28821259 | Decoy | ZINC48811196 | Decoy |
| ZINC04691548 | Decoy | ZINC28823877 | Decoy | ZINC48813033 | Decoy |
| ZINC04693861 | Decoy | ZINC28865261 | Decoy | ZINC48815623 | Decoy |
| ZINC04703373 | Decoy | ZINC28889554 | Decoy | ZINC48816007 | Decoy |
| ZINC04712141 | Decoy | ZINC28897304 | Decoy | ZINC48825293 | Decoy |
| ZINC04724459 | Decoy | ZINC28903064 | Decoy | ZINC48835557 | Decoy |
| ZINC04737420 | Decoy | ZINC28907272 | Decoy | ZINC48841520 | Decoy |
| ZINC04738319 | Decoy | ZINC28909033 | Decoy | ZINC48845285 | Decoy |
| ZINC04744256 | Decoy | ZINC28927852 | Decoy | ZINC48907626 | Decoy |
| ZINC04745705 | Decoy | ZINC29024777 | Decoy | ZINC48918803 | Decoy |
| ZINC04749957 | Decoy | ZINC29039927 | Decoy | ZINC48940832 | Decoy |
| ZINC04754491 | Decoy | ZINC29042317 | Decoy | ZINC48954386 | Decoy |
| ZINC04759330 | Decoy | ZINC29046093 | Decoy | ZINC48962601 | Decoy |
| ZINC04778108 | Decoy | ZINC29053968 | Decoy | ZINC48963859 | Decoy |
| ZINC04787131 | Decoy | ZINC29058469 | Decoy | ZINC48963882 | Decoy |
| ZINC04791856 | Decoy | ZINC29074758 | Decoy | ZINC48968157 | Decoy |
| ZINC04805734 | Decoy | ZINC29124703 | Decoy | ZINC48969036 | Decoy |
| ZINC04814480 | Decoy | ZINC29130319 | Decoy | ZINC48982344 | Decoy |
| ZINC04818955 | Decoy | ZINC29131501 | Decoy | ZINC48998305 | Decoy |
| ZINC04819321 | Decoy | ZINC29132077 | Decoy | ZINC48998839 | Decoy |
| ZINC04826688 | Decoy | ZINC29136123 | Decoy | ZINC49028472 | Decoy |
| ZINC04830706 | Decoy | ZINC29189880 | Decoy | ZINC49033661 | Decoy |
| ZINC04834371 | Decoy | ZINC29210546 | Decoy | ZINC49055702 | Decoy |
| ZINC04841939 | Decoy | ZINC29218162 | Decoy | ZINC49068791 | Decoy |
| ZINC04868984 | Decoy | ZINC29218771 | Decoy | ZINC49071495 | Decoy |
| ZINC04881155 | Decoy | ZINC29250548 | Decoy | ZINC49071801 | Decoy |
| ZINC04891664 | Decoy | ZINC29252932 | Decoy | ZINC49075775 | Decoy |
| ZINC04893087 | Decoy | ZINC29253807 | Decoy | ZINC49077183 | Decoy |
| ZINC04896783 | Decoy | ZINC29282014 | Decoy | ZINC49084476 | Decoy |
| ZINC04906993 | Decoy | ZINC29301484 | Decoy | ZINC49089509 | Decoy |
| ZINC04907213 | Decoy | ZINC29318341 | Decoy | ZINC49091751 | Decoy |
| ZINC04918848 | Decoy | ZINC29318763 | Decoy | ZINC49113470 | Decoy |
| ZINC04920470 | Decoy | ZINC29320029 | Decoy | ZINC49115232 | Decoy |
| ZINC04925116 | Decoy | ZINC29320324 | Decoy | ZINC49120132 | Decoy |
| ZINC04933880 | Decoy | ZINC29320396 | Decoy | ZINC49122773 | Decoy |
| ZINC04937197 | Decoy | ZINC29335431 | Decoy | ZINC49125744 | Decoy |
| ZINC04942932 | Decoy | ZINC29351944 | Decoy | ZINC49126307 | Decoy |
| ZINC04952485 | Decoy | ZINC29378732 | Decoy | ZINC49138629 | Decoy |
| ZINC04952531 | Decoy | ZINC29380355 | Decoy | ZINC49142189 | Decoy |
| ZINC04954813 | Decoy | ZINC29388903 | Decoy | ZINC49144221 | Decoy |
| ZINC04955231 | Decoy | ZINC29394240 | Decoy | ZINC49152111 | Decoy |
| ZINC04962511 | Decoy | ZINC29398791 | Decoy | ZINC49153139 | Decoy |
| ZINC04967342 | Decoy | ZINC29403088 | Decoy | ZINC49157879 | Decoy |
| ZINC04968534 | Decoy | ZINC29405991 | Decoy | ZINC49176790 | Decoy |
| ZINC04969070 | Decoy | ZINC29411418 | Decoy | ZINC49177291 | Decoy |
| ZINC04976426 | Decoy | ZINC29412279 | Decoy | ZINC49198267 | Decoy |
| ZINC04981393 | Decoy | ZINC29413051 | Decoy | ZINC49220096 | Decoy |
| ZINC04988988 | Decoy | ZINC29413703 | Decoy | ZINC49228067 | Decoy |
| ZINC04990104 | Decoy | ZINC29459596 | Decoy | ZINC49231880 | Decoy |
| ZINC05003900 | Decoy | ZINC29460303 | Decoy | ZINC49233999 | Decoy |
| ZINC05005629 | Decoy | ZINC29460427 | Decoy | ZINC49240981 | Decoy |
| ZINC05006153 | Decoy | ZINC29460436 | Decoy | ZINC49241009 | Decoy |
| ZINC05010352 | Decoy | ZINC29462044 | Decoy | ZINC49241295 | Decoy |
| ZINC05011795 | Decoy | ZINC29463132 | Decoy | ZINC49251752 | Decoy |
| ZINC05016183 | Decoy | ZINC29499564 | Decoy | ZINC49254914 | Decoy |
| ZINC05019191 | Decoy | ZINC29507186 | Decoy | ZINC49268092 | Decoy |
| ZINC05023080 | Decoy | ZINC29526573 | Decoy | ZINC49276086 | Decoy |
| ZINC05027393 | Decoy | ZINC29530010 | Decoy | ZINC49281233 | Decoy |
| ZINC05030118 | Decoy | ZINC29554062 | Decoy | ZINC49286881 | Decoy |
| ZINC05031156 | Decoy | ZINC29554801 | Decoy | ZINC49289216 | Decoy |
| ZINC05032639 | Decoy | ZINC29578835 | Decoy | ZINC49289359 | Decoy |
| ZINC05033922 | Decoy | ZINC29579490 | Decoy | ZINC49290025 | Decoy |
| ZINC05037202 | Decoy | ZINC29579688 | Decoy | ZINC49297369 | Decoy |
| ZINC05050089 | Decoy | ZINC29580816 | Decoy | ZINC49297542 | Decoy |
| ZINC05050350 | Decoy | ZINC29584557 | Decoy | ZINC49301547 | Decoy |
| ZINC05062319 | Decoy | ZINC29586983 | Decoy | ZINC49354648 | Decoy |
| ZINC05076116 | Decoy | ZINC29654968 | Decoy | ZINC49357425 | Decoy |
| ZINC05078339 | Decoy | ZINC29656761 | Decoy | ZINC49379675 | Decoy |
| ZINC05103407 | Decoy | ZINC29713466 | Decoy | ZINC49397191 | Decoy |
| ZINC05114112 | Decoy | ZINC29751172 | Decoy | ZINC49406337 | Decoy |
| ZINC05127587 | Decoy | ZINC29795333 | Decoy | ZINC49407122 | Decoy |
| ZINC05171899 | Decoy | ZINC29799903 | Decoy | ZINC49409699 | Decoy |
| ZINC05175422 | Decoy | ZINC29839015 | Decoy | ZINC49413780 | Decoy |
| ZINC05186782 | Decoy | ZINC29854491 | Decoy | ZINC49414072 | Decoy |
| ZINC05228094 | Decoy | ZINC29859095 | Decoy | ZINC49418897 | Decoy |
| ZINC05234193 | Decoy | ZINC29952858 | Decoy | ZINC49423217 | Decoy |
| ZINC05239743 | Decoy | ZINC30000647 | Decoy | ZINC49427801 | Decoy |
| ZINC05250059 | Decoy | ZINC30117618 | Decoy | ZINC49442767 | Decoy |
| ZINC05250103 | Decoy | ZINC30516110 | Decoy | ZINC49449137 | Decoy |
| ZINC05251501 | Decoy | ZINC30544496 | Decoy | ZINC49453837 | Decoy |
| ZINC05278930 | Decoy | ZINC30631263 | Decoy | ZINC49462398 | Decoy |
| ZINC05283195 | Decoy | ZINC30638424 | Decoy | ZINC49471241 | Decoy |
| ZINC05328773 | Decoy | ZINC30645234 | Decoy | ZINC49471784 | Decoy |
| ZINC05330608 | Decoy | ZINC30655934 | Decoy | ZINC49472534 | Decoy |
| ZINC05331643 | Decoy | ZINC30670232 | Decoy | ZINC49473446 | Decoy |
| ZINC05337307 | Decoy | ZINC30670239 | Decoy | ZINC49476379 | Decoy |
| ZINC05339773 | Decoy | ZINC30822897 | Decoy | ZINC49478521 | Decoy |
| ZINC05377914 | Decoy | ZINC30873004 | Decoy | ZINC49478679 | Decoy |
| ZINC05386774 | Decoy | ZINC30902706 | Decoy | ZINC49487992 | Decoy |
| ZINC05393976 | Decoy | ZINC30902729 | Decoy | ZINC49491688 | Decoy |
| ZINC05394859 | Decoy | ZINC30908023 | Decoy | ZINC49496077 | Decoy |
| ZINC05418606 | Decoy | ZINC30921025 | Decoy | ZINC49519466 | Decoy |
| ZINC05425251 | Decoy | ZINC30953545 | Decoy | ZINC49522249 | Decoy |
| ZINC05438022 | Decoy | ZINC30985250 | Decoy | ZINC49523959 | Decoy |
| ZINC05453991 | Decoy | ZINC30985366 | Decoy | ZINC49524672 | Decoy |
| ZINC05458828 | Decoy | ZINC30989492 | Decoy | ZINC49626539 | Decoy |
| ZINC05472616 | Decoy | ZINC31032294 | Decoy | ZINC49766156 | Decoy |
| ZINC05491120 | Decoy | ZINC31033764 | Decoy | ZINC49784259 | Decoy |
| ZINC05492803 | Decoy | ZINC31048228 | Decoy | ZINC49784757 | Decoy |
| ZINC05521114 | Decoy | ZINC31116271 | Decoy | ZINC49793820 | Decoy |
| ZINC05534882 | Decoy | ZINC31141338 | Decoy | ZINC49802929 | Decoy |
| ZINC05541837 | Decoy | ZINC31146212 | Decoy | ZINC49808483 | Decoy |
| ZINC05551296 | Decoy | ZINC31208661 | Decoy | ZINC49844070 | Decoy |
| ZINC05566603 | Decoy | ZINC31306272 | Decoy | ZINC49850478 | Decoy |
| ZINC05584064 | Decoy | ZINC31311317 | Decoy | ZINC49909183 | Decoy |
| ZINC05606573 | Decoy | ZINC31311345 | Decoy | ZINC49925093 | Decoy |
| ZINC05642512 | Decoy | ZINC31320347 | Decoy | ZINC49951583 | Decoy |
| ZINC05673328 | Decoy | ZINC31343408 | Decoy | ZINC49968109 | Decoy |
| ZINC05677754 | Decoy | ZINC31451205 | Decoy | ZINC49973554 | Decoy |
| ZINC05699162 | Decoy | ZINC31453818 | Decoy | ZINC49983987 | Decoy |
| ZINC05703830 | Decoy | ZINC31551298 | Decoy | ZINC50003685 | Decoy |
| ZINC05717093 | Decoy | ZINC31559662 | Decoy | ZINC50003826 | Decoy |
| ZINC05719560 | Decoy | ZINC31607199 | Decoy | ZINC50008220 | Decoy |
| ZINC05768916 | Decoy | ZINC31724879 | Decoy | ZINC50025150 | Decoy |
| ZINC05777285 | Decoy | ZINC31728739 | Decoy | ZINC50031629 | Decoy |
| ZINC05792767 | Decoy | ZINC31776901 | Decoy | ZINC50134667 | Decoy |
| ZINC05811400 | Decoy | ZINC31870554 | Decoy | ZINC50140031 | Decoy |
| ZINC05812031 | Decoy | ZINC32003747 | Decoy | ZINC50178628 | Decoy |
| ZINC05817293 | Decoy | ZINC32124749 | Decoy | ZINC50273375 | Decoy |
| ZINC05846729 | Decoy | ZINC32336116 | Decoy | ZINC50273760 | Decoy |
| ZINC05886073 | Decoy | ZINC32445535 | Decoy | ZINC50378582 | Decoy |
| ZINC05888089 | Decoy | ZINC32458237 | Decoy | ZINC50378696 | Decoy |
| ZINC05907439 | Decoy | ZINC32474517 | Decoy | ZINC50383854 | Decoy |
| ZINC05908625 | Decoy | ZINC32477835 | Decoy | ZINC50402129 | Decoy |
| ZINC05919660 | Decoy | ZINC32504101 | Decoy | ZINC50469492 | Decoy |
| ZINC05928781 | Decoy | ZINC32518071 | Decoy | ZINC50498856 | Decoy |
| ZINC05937311 | Decoy | ZINC32540500 | Decoy | ZINC50508147 | Decoy |
| ZINC05940541 | Decoy | ZINC32561085 | Decoy | ZINC50509976 | Decoy |
| ZINC05958696 | Decoy | ZINC32573257 | Decoy | ZINC50549914 | Decoy |
| ZINC05962685 | Decoy | ZINC32577654 | Decoy | ZINC50561026 | Decoy |
| ZINC05962878 | Decoy | ZINC32578376 | Decoy | ZINC50586563 | Decoy |
| ZINC05964836 | Decoy | ZINC32598420 | Decoy | ZINC50602953 | Decoy |
| ZINC05976926 | Decoy | ZINC32626888 | Decoy | ZINC50610385 | Decoy |
| ZINC05978063 | Decoy | ZINC32652242 | Decoy | ZINC50613355 | Decoy |
| ZINC06026609 | Decoy | ZINC32665608 | Decoy | ZINC50662813 | Decoy |
| ZINC06042327 | Decoy | ZINC32695842 | Decoy | ZINC50665898 | Decoy |
| ZINC06069493 | Decoy | ZINC32699317 | Decoy | ZINC50665909 | Decoy |
| ZINC06082892 | Decoy | ZINC32729823 | Decoy | ZINC50666528 | Decoy |
| ZINC06127216 | Decoy | ZINC32753223 | Decoy | ZINC50668878 | Decoy |
| ZINC06184664 | Decoy | ZINC32753566 | Decoy | ZINC50669135 | Decoy |
| ZINC06196622 | Decoy | ZINC32760756 | Decoy | ZINC50680431 | Decoy |
| ZINC06241939 | Decoy | ZINC32764885 | Decoy | ZINC50684487 | Decoy |
| ZINC06270362 | Decoy | ZINC32765628 | Decoy | ZINC50731765 | Decoy |
| ZINC06383886 | Decoy | ZINC32768536 | Decoy | ZINC50765396 | Decoy |
| ZINC06384153 | Decoy | ZINC32776727 | Decoy | ZINC50765860 | Decoy |
| ZINC06411270 | Decoy | ZINC32784322 | Decoy | ZINC50766536 | Decoy |
| ZINC06431179 | Decoy | ZINC32806431 | Decoy | ZINC50766970 | Decoy |
| ZINC06450801 | Decoy | ZINC32819089 | Decoy | ZINC50767069 | Decoy |
| ZINC06459335 | Decoy | ZINC32825413 | Decoy | ZINC50767119 | Decoy |
| ZINC06471343 | Decoy | ZINC32828598 | Decoy | ZINC50767193 | Decoy |
| ZINC06471986 | Decoy | ZINC32855777 | Decoy | ZINC50770997 | Decoy |
| ZINC06508059 | Decoy | ZINC32907051 | Decoy | ZINC50782701 | Decoy |
| ZINC06535656 | Decoy | ZINC32907910 | Decoy | ZINC50782838 | Decoy |
| ZINC06548566 | Decoy | ZINC32919694 | Decoy | ZINC50797421 | Decoy |
| ZINC06560829 | Decoy | ZINC32962531 | Decoy | ZINC50797779 | Decoy |
| ZINC06583482 | Decoy | ZINC32986570 | Decoy | ZINC50799456 | Decoy |
| ZINC06604385 | Decoy | ZINC32999815 | Decoy | ZINC50799853 | Decoy |
| ZINC06643330 | Decoy | ZINC33018740 | Decoy | ZINC50802241 | Decoy |
| ZINC06646295 | Decoy | ZINC33084960 | Decoy | ZINC50825251 | Decoy |
| ZINC06677800 | Decoy | ZINC33087265 | Decoy | ZINC50826902 | Decoy |
| ZINC06681867 | Decoy | ZINC33135296 | Decoy | ZINC50862405 | Decoy |
| ZINC06682022 | Decoy | ZINC33145600 | Decoy | ZINC50898015 | Decoy |
| ZINC06697810 | Decoy | ZINC33191197 | Decoy | ZINC50916008 | Decoy |
| ZINC06701617 | Decoy | ZINC33275343 | Decoy | ZINC50931607 | Decoy |
| ZINC06703450 | Decoy | ZINC33465092 | Decoy | ZINC50932721 | Decoy |
| ZINC06725571 | Decoy | ZINC33885406 | Decoy | ZINC50932736 | Decoy |
| ZINC06730877 | Decoy | ZINC34201940 | Decoy | ZINC50954370 | Decoy |
| ZINC06742363 | Decoy | ZINC34259677 | Decoy | ZINC50954379 | Decoy |
| ZINC06745970 | Decoy | ZINC34308671 | Decoy | ZINC50954386 | Decoy |
| ZINC06761714 | Decoy | ZINC34426381 | Decoy | ZINC50954609 | Decoy |
| ZINC06815721 | Decoy | ZINC34545680 | Decoy | ZINC50958181 | Decoy |
| ZINC06857373 | Decoy | ZINC34790483 | Decoy | ZINC50988792 | Decoy |
| ZINC06869655 | Decoy | ZINC34882115 | Decoy | ZINC50989048 | Decoy |
| ZINC06870735 | Decoy | ZINC34924964 | Decoy | ZINC50989162 | Decoy |
| ZINC06875778 | Decoy | ZINC34956771 | Decoy | ZINC50994460 | Decoy |
| ZINC06895939 | Decoy | ZINC35048183 | Decoy | ZINC50996899 | Decoy |
| ZINC06981321 | Decoy | ZINC35056183 | Decoy | ZINC50997346 | Decoy |
| ZINC07007799 | Decoy | ZINC35132808 | Decoy | ZINC50998067 | Decoy |
| ZINC07032570 | Decoy | ZINC35186444 | Decoy | ZINC50998694 | Decoy |
| ZINC07107855 | Decoy | ZINC35186952 | Decoy | ZINC50998910 | Decoy |
| ZINC07138992 | Decoy | ZINC35191181 | Decoy | ZINC50999075 | Decoy |
| ZINC07157650 | Decoy | ZINC35247364 | Decoy | ZINC51000525 | Decoy |
| ZINC07261310 | Decoy | ZINC35464065 | Decoy | ZINC51027050 | Decoy |
| ZINC07261724 | Decoy | ZINC35562017 | Decoy | ZINC51034680 | Decoy |
| ZINC07272702 | Decoy | ZINC35617845 | Decoy | ZINC51049147 | Decoy |
| ZINC07273236 | Decoy | ZINC35739268 | Decoy | ZINC51086923 | Decoy |
| ZINC07276439 | Decoy | ZINC35758959 | Decoy | ZINC51092483 | Decoy |
| ZINC07276466 | Decoy | ZINC35973997 | Decoy | ZINC51121015 | Decoy |
| ZINC07381994 | Decoy | ZINC35999449 | Decoy | ZINC51171744 | Decoy |
| ZINC07386655 | Decoy | ZINC36070299 | Decoy | ZINC51181006 | Decoy |
| ZINC07492988 | Decoy | ZINC36076991 | Decoy | ZINC51188919 | Decoy |
| ZINC07524906 | Decoy | ZINC36077520 | Decoy | ZINC51219895 | Decoy |
| ZINC07525166 | Decoy | ZINC36082780 | Decoy | ZINC51220405 | Decoy |
| ZINC07538620 | Decoy | ZINC36090050 | Decoy | ZINC51279825 | Decoy |
| ZINC07539364 | Decoy | ZINC36093155 | Decoy | ZINC51283069 | Decoy |
| ZINC07559772 | Decoy | ZINC36093302 | Decoy | ZINC51285979 | Decoy |
| ZINC07559804 | Decoy | ZINC36094357 | Decoy | ZINC51314736 | Decoy |
| ZINC07572464 | Decoy | ZINC36105370 | Decoy | ZINC51323007 | Decoy |
| ZINC07578372 | Decoy | ZINC36109178 | Decoy | ZINC51347720 | Decoy |
| ZINC07607939 | Decoy | ZINC36119332 | Decoy | ZINC51352743 | Decoy |
| ZINC07612817 | Decoy | ZINC36136645 | Decoy | ZINC51375429 | Decoy |
| ZINC07613262 | Decoy | ZINC36164496 | Decoy | ZINC51375888 | Decoy |
| ZINC07637264 | Decoy | ZINC36187784 | Decoy | ZINC51377608 | Decoy |
| ZINC07666236 | Decoy | ZINC36193325 | Decoy | ZINC51383031 | Decoy |
| ZINC07676161 | Decoy | ZINC36221600 | Decoy | ZINC51410753 | Decoy |
| ZINC07689588 | Decoy | ZINC36221620 | Decoy | ZINC51411127 | Decoy |
| ZINC07714261 | Decoy | ZINC36297641 | Decoy | ZINC51411172 | Decoy |
| ZINC07714359 | Decoy | ZINC36305545 | Decoy | ZINC51412109 | Decoy |
| ZINC07736218 | Decoy | ZINC36318196 | Decoy | ZINC51419316 | Decoy |
| ZINC07743353 | Decoy | ZINC36322074 | Decoy | ZINC51428063 | Decoy |
| ZINC07743747 | Decoy | ZINC36332066 | Decoy | ZINC51453681 | Decoy |
| ZINC07744382 | Decoy | ZINC36376573 | Decoy | ZINC51454557 | Decoy |
| ZINC07744907 | Decoy | ZINC36377255 | Decoy | ZINC51471123 | Decoy |
| ZINC07746676 | Decoy | ZINC36378060 | Decoy | ZINC51472568 | Decoy |
| ZINC07760179 | Decoy | ZINC36378068 | Decoy | ZINC51476338 | Decoy |
| ZINC07760505 | Decoy | ZINC36382481 | Decoy | ZINC51502349 | Decoy |
| ZINC07760657 | Decoy | ZINC36386714 | Decoy | ZINC51506507 | Decoy |
| ZINC07760738 | Decoy | ZINC36387682 | Decoy | ZINC51531314 | Decoy |
| ZINC07762914 | Decoy | ZINC36408636 | Decoy | ZINC51533154 | Decoy |
| ZINC07771041 | Decoy | ZINC36417647 | Decoy | ZINC51538529 | Decoy |
| ZINC07777626 | Decoy | ZINC36466000 | Decoy | ZINC51555749 | Decoy |
| ZINC07779087 | Decoy | ZINC36476389 | Decoy | ZINC51559864 | Decoy |
| ZINC07794333 | Decoy | ZINC36493259 | Decoy | ZINC51589684 | Decoy |
| ZINC07842751 | Decoy | ZINC36533300 | Decoy | ZINC51648618 | Decoy |
| ZINC07923415 | Decoy | ZINC36576220 | Decoy | ZINC51739637 | Decoy |
| ZINC07972812 | Decoy | ZINC36618152 | Decoy | ZINC51753730 | Decoy |
| ZINC07977039 | Decoy | ZINC36629948 | Decoy | ZINC51754143 | Decoy |
| ZINC07979787 | Decoy | ZINC36638051 | Decoy | ZINC51758385 | Decoy |
| ZINC07995452 | Decoy | ZINC36647428 | Decoy | ZINC51759874 | Decoy |
| ZINC07996815 | Decoy | ZINC36656223 | Decoy | ZINC51792216 | Decoy |
| ZINC08032427 | Decoy | ZINC36694802 | Decoy | ZINC51800932 | Decoy |
| ZINC08145581 | Decoy | ZINC36742319 | Decoy | ZINC51832650 | Decoy |
| ZINC08148743 | Decoy | ZINC36752965 | Decoy | ZINC51846690 | Decoy |
| ZINC08153199 | Decoy | ZINC36758346 | Decoy | ZINC51855681 | Decoy |
| ZINC08165544 | Decoy | ZINC36874349 | Decoy | ZINC51908009 | Decoy |
| ZINC08247467 | Decoy | ZINC37164048 | Decoy | ZINC51925257 | Decoy |
| ZINC08253374 | Decoy | ZINC37176717 | Decoy | ZINC51960029 | Decoy |
| ZINC08254454 | Decoy | ZINC37390949 | Decoy | ZINC51964492 | Decoy |
| ZINC08315030 | Decoy | ZINC37439919 | Decoy | ZINC51980323 | Decoy |
| ZINC08325899 | Decoy | ZINC37474906 | Decoy | ZINC51999572 | Decoy |
| ZINC08325978 | Decoy | ZINC37499381 | Decoy | ZINC52004738 | Decoy |
| ZINC08337985 | Decoy | ZINC37506205 | Decoy | ZINC52005954 | Decoy |
| ZINC08346063 | Decoy | ZINC37599986 | Decoy | ZINC52006137 | Decoy |
| ZINC08368910 | Decoy | ZINC37638091 | Decoy | ZINC52039200 | Decoy |
| ZINC08395581 | Decoy | ZINC37751745 | Decoy | ZINC52049402 | Decoy |
| ZINC08411436 | Decoy | ZINC37790825 | Decoy | ZINC52049469 | Decoy |
| ZINC08414138 | Decoy | ZINC37842016 | Decoy | ZINC52049569 | Decoy |
| ZINC08433431 | Decoy | ZINC37867742 | Decoy | ZINC52050014 | Decoy |
| ZINC08529708 | Decoy | ZINC37990054 | Decoy | ZINC52050015 | Decoy |
| ZINC08530375 | Decoy | ZINC37990259 | Decoy | ZINC52070298 | Decoy |
| ZINC08535098 | Decoy | ZINC37990326 | Decoy | ZINC52070926 | Decoy |
| ZINC08558183 | Decoy | ZINC37990452 | Decoy | ZINC52073996 | Decoy |
| ZINC08584309 | Decoy | ZINC37990525 | Decoy | ZINC52074340 | Decoy |
| ZINC08613692 | Decoy | ZINC38032217 | Decoy | ZINC52075429 | Decoy |
| ZINC08615521 | Decoy | ZINC38084337 | Decoy | ZINC52077026 | Decoy |
| ZINC08635610 | Decoy | ZINC38153405 | Decoy | ZINC52077884 | Decoy |
| ZINC08635611 | Decoy | ZINC38189932 | Decoy | ZINC52087173 | Decoy |
| ZINC08638144 | Decoy | ZINC38283080 | Decoy | ZINC52087532 | Decoy |
| ZINC08672854 | Decoy | ZINC38338755 | Decoy | ZINC52101362 | Decoy |
| ZINC08699504 | Decoy | ZINC38343307 | Decoy | ZINC52105463 | Decoy |
| ZINC08715595 | Decoy | ZINC38415504 | Decoy | ZINC52105501 | Decoy |
| ZINC08718499 | Decoy | ZINC38488399 | Decoy | ZINC52119733 | Decoy |
| ZINC08728108 | Decoy | ZINC38511647 | Decoy | ZINC52120212 | Decoy |
| ZINC08737820 | Decoy | ZINC38573949 | Decoy | ZINC52128157 | Decoy |
| ZINC08743818 | Decoy | ZINC38579441 | Decoy | ZINC52129005 | Decoy |
| ZINC08750473 | Decoy | ZINC38857479 | Decoy | ZINC52132444 | Decoy |
| ZINC08774587 | Decoy | ZINC38960837 | Decoy | ZINC52163525 | Decoy |
| ZINC08775644 | Decoy | ZINC39187955 | Decoy | ZINC52163554 | Decoy |
| ZINC08778668 | Decoy | ZINC39365200 | Decoy | ZINC52163973 | Decoy |
| ZINC08851296 | Decoy | ZINC39398457 | Decoy | ZINC52164128 | Decoy |
| ZINC08907593 | Decoy | ZINC39439236 | Decoy | ZINC52183986 | Decoy |
| ZINC08940495 | Decoy | ZINC39560699 | Decoy | ZINC52183988 | Decoy |
| ZINC08965405 | Decoy | ZINC39979583 | Decoy | ZINC52184163 | Decoy |
| ZINC09052174 | Decoy | ZINC40012043 | Decoy | ZINC52184376 | Decoy |
| ZINC09142058 | Decoy | ZINC40023982 | Decoy | ZINC52189761 | Decoy |
| ZINC09231655 | Decoy | ZINC40058865 | Decoy | ZINC52206668 | Decoy |
| ZINC09256060 | Decoy | ZINC40070125 | Decoy | ZINC52208483 | Decoy |
| ZINC09276399 | Decoy | ZINC40072798 | Decoy | ZINC52208570 | Decoy |
| ZINC09344799 | Decoy | ZINC40095286 | Decoy | ZINC52208665 | Decoy |
| ZINC09434842 | Decoy | ZINC40106480 | Decoy | ZINC52208700 | Decoy |
| ZINC09444000 | Decoy | ZINC40156760 | Decoy | ZINC52209467 | Decoy |
| ZINC09465469 | Decoy | ZINC40331218 | Decoy | ZINC52218525 | Decoy |
| ZINC09479406 | Decoy | ZINC40358994 | Decoy | ZINC52220008 | Decoy |
| ZINC09493618 | Decoy | ZINC40373205 | Decoy | ZINC52245039 | Decoy |
| ZINC09501342 | Decoy | ZINC40393362 | Decoy | ZINC52245923 | Decoy |
| ZINC09502043 | Decoy | ZINC40395826 | Decoy | ZINC52331068 | Decoy |
| ZINC09513576 | Decoy | ZINC40406621 | Decoy | ZINC52357412 | Decoy |
| ZINC09730174 | Decoy | ZINC40412725 | Decoy | ZINC52405793 | Decoy |
| ZINC09743511 | Decoy | ZINC40422783 | Decoy | ZINC52426262 | Decoy |
| ZINC09752943 | Decoy | ZINC40423448 | Decoy | ZINC52446300 | Decoy |
| ZINC09836686 | Decoy | ZINC40424230 | Decoy | ZINC52467739 | Decoy |
| ZINC09841058 | Decoy | ZINC40430765 | Decoy | ZINC52469392 | Decoy |
| ZINC09870848 | Decoy | ZINC40436615 | Decoy | ZINC52473207 | Decoy |
| ZINC09888788 | Decoy | ZINC40436643 | Decoy | ZINC52473590 | Decoy |
| ZINC10035335 | Decoy | ZINC40443187 | Decoy | ZINC52502560 | Decoy |
| ZINC10058118 | Decoy | ZINC40444847 | Decoy | ZINC52522298 | Decoy |
| ZINC10213171 | Decoy | ZINC40445033 | Decoy | ZINC52530425 | Decoy |
| ZINC10214554 | Decoy | ZINC40460530 | Decoy | ZINC52550583 | Decoy |
| ZINC10315517 | Decoy | ZINC40465171 | Decoy | ZINC52551106 | Decoy |
| ZINC10315519 | Decoy | ZINC40466462 | Decoy | ZINC52554425 | Decoy |
| ZINC10325356 | Decoy | ZINC40488676 | Decoy | ZINC52565787 | Decoy |
| ZINC10360155 | Decoy | ZINC40500254 | Decoy | ZINC52566249 | Decoy |
| ZINC10365364 | Decoy | ZINC40504085 | Decoy | ZINC52569921 | Decoy |
| ZINC10434743 | Decoy | ZINC40556669 | Decoy | ZINC52569952 | Decoy |
| ZINC10435332 | Decoy | ZINC40565801 | Decoy | ZINC52569954 | Decoy |
| ZINC10436050 | Decoy | ZINC40614842 | Decoy | ZINC52569966 | Decoy |
| ZINC10436529 | Decoy | ZINC40629241 | Decoy | ZINC52572152 | Decoy |
| ZINC10472096 | Decoy | ZINC40644744 | Decoy | ZINC52584604 | Decoy |
| ZINC10669355 | Decoy | ZINC40667603 | Decoy | ZINC52606532 | Decoy |
| ZINC10959352 | Decoy | ZINC40667712 | Decoy | ZINC52609706 | Decoy |
| ZINC11104174 | Decoy | ZINC40667896 | Decoy | ZINC52618702 | Decoy |
| ZINC11122358 | Decoy | ZINC40714629 | Decoy | ZINC52627727 | Decoy |
| ZINC11153931 | Decoy | ZINC40719086 | Decoy | ZINC52627743 | Decoy |
| ZINC11157087 | Decoy | ZINC40719349 | Decoy | ZINC52634377 | Decoy |
| ZINC11162129 | Decoy | ZINC40719586 | Decoy | ZINC52635041 | Decoy |
| ZINC11169644 | Decoy | ZINC40719772 | Decoy | ZINC52635086 | Decoy |
| ZINC11170271 | Decoy | ZINC40721113 | Decoy | ZINC52636980 | Decoy |
| ZINC11234300 | Decoy | ZINC40721136 | Decoy | ZINC52660932 | Decoy |
| ZINC11289538 | Decoy | ZINC40722051 | Decoy | ZINC52666306 | Decoy |
| ZINC11376985 | Decoy | ZINC40722270 | Decoy | ZINC52713518 | Decoy |
| ZINC11479769 | Decoy | ZINC40723896 | Decoy | ZINC52713757 | Decoy |
| ZINC11486419 | Decoy | ZINC40728573 | Decoy | ZINC52713777 | Decoy |
| ZINC11486444 | Decoy | ZINC40728711 | Decoy | ZINC52719914 | Decoy |
| ZINC11486520 | Decoy | ZINC40729215 | Decoy | ZINC52742254 | Decoy |
| ZINC11618647 | Decoy | ZINC40730685 | Decoy | ZINC52807627 | Decoy |
| ZINC11708539 | Decoy | ZINC40731142 | Decoy | ZINC52819050 | Decoy |
| ZINC11735692 | Decoy | ZINC40731372 | Decoy | ZINC52826708 | Decoy |
| ZINC11749747 | Decoy | ZINC40739070 | Decoy | ZINC52831584 | Decoy |
| ZINC11783274 | Decoy | ZINC40741963 | Decoy | ZINC05812031 | Decoy |

**2S Table. Detailed list of ligands from GLL and GDD datasets with activity tags.**
